# Maximizing Efficacy of Cancer Nanovaccines and Immune Cells Landscape in Responders and Non‐Responders to Immunotherapy

**DOI:** 10.1002/advs.202416756

**Published:** 2025-06-26

**Authors:** Xiangxiang Xu, Lu Diao, Jin Wang, Ao Zhu, Xianlan Chen, Yan Zheng, Yuhan Liu, Kang Hu, Jiashan Zhu, Cheng Ding, Chang Li, Yunzhi Pan, Jun Zhao, Mi Liu

**Affiliations:** ^1^ Department of Pharmaceutics College of Pharmaceutical Sciences Soochow University Suzhou Jiangsu 215123 P. R. China; ^2^ Institute of Minimally Invasive Thoracic Cancer Therapy and Translational Research Soochow University Suzhou Jiangsu 215123 P. R. China; ^3^ Suzhou Ersheng Biopharmaceutical Co., Ltd Suzhou Jiangsu 215000 P. R. China; ^4^ Wuxi Boston Biopharmaceutical Co., Ltd Wuxi Jiangsu 214125 P. R. China; ^5^ Institute of Thoracic Surgery The First Affiliated Hospital of Soochow University Soochow University Suzhou Jiangsu 215123 P. R. China; ^6^ Department of Thoracic Surgery The First Affiliated Hospital of Soochow University Soochow University Suzhou Jiangsu 215123 P. R. China; ^7^ Department of Pharmacy The Affiliated Infectious Diseases Hospital of Soochow University Suzhou Jiangsu 215000 P. R. China; ^8^ Jiangsu Province Engineering Research Center of Precision Diagnostics and Therapeutics Development Soochow University Suzhou Jiangsu 215123 P. R. China

**Keywords:** biomarker, cancer vaccine, immunotherapy, predictor, T cells

## Abstract

Adjuvants, formulations, and processing of tumor antigens (collection, lysis, purification, and oxidation) can affect the therapeutic efficacy of cancer vaccines. To maximize their efficacy, adjuvants and their combinations are investigated to prepare vaccines with whole tumor antigens. By comparing different nano/micro‐vaccines, ≤ 400 nm and 2.5 µm, respectively, are identified as their optimal sizes. When used alone, the optimal cancer vaccine cures all or most tumor‐bearing mice with melanoma, lung cancer, pancreatic cancer, and melanoma lung metastasis. The landscape of immune cells in the blood, splenocytes, and draining lymph nodes of vaccine‐treated mice is systematically investigated using single‐cell sequencing. The diversity of CD8^+^ T cell receptors (TCR), CD4^+^ TCR, and B cell receptors (BCR) in cured mice is higher than that in uncured mice. By comparing 21 samples, several biomarkers, including KLRG1, S100A4, S1PR5, IL2Ra, and IKZF2, were identified to distinguish responders and non‐responders to immunotherapy. Moreover, S100A4, S1PR5 and KLRG1, are biomarkers of therapeutic efficacy in mouse cancer models and patients with cancer. Hence, this study presents an optimized cancer vaccine that cures most tumor‐bearing mouse models and the landscape of immune cells in non‐responders and responders to immunotherapy. The results of this study will help to develop better cancer vaccines.

## Graphical abstract

To maximize the efficacy of cancer vaccines, adjuvants, collection, lysis, purification, oxidation, loading sites, and sizes, etc., are investigated. The optimized cancer nanovaccines loading whole tumor antigens can cure all or most tumor‐bearing mice in multiple mouse cancer models. Moreover, the landscape of immune cells in the blood, splenocytes, and draining lymph nodes of vaccine‐treated mice is studied and some biomarkers of non‐responders to immunotherapy are identified.

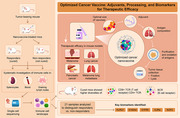

## 1 Introduction

With the development of an aging society, the number of cancer patients is increasing annually. Cancer is a malignant disease with a high mortality rate and strong unmet clinical need. Cancer drug treatments can be broadly divided into immunotherapy and non‐immunotherapy. Non‐immunotherapies or conventional treatments for cancer include surgical resection, chemotherapy, targeted therapy, high temperature therapy or cryoablation, and radiotherapy etc. Cancer immunotherapy includes immune‐checkpoint inhibitors (including PD‐1, PD‐L1, and CTLA‐4 antibodies), T‐cell‐based therapy, (such as CAR‐T, TILs, TCR‐T, and in situ editing of T cells, etc.), therapy based on the innate immune system (including NK cells, γδ T cell therapy, NK‐T cells, CAR‐NK, and cytokine therapy, etc), oncolytic virus, oncolytic bacteria, and cancer vaccines, including different vaccine forms such as new antigen and dendritic cells (DC) vaccines, etc. Each of these methods has its advantages and disadvantages.

One aim of cancer therapy is to transform cancer from a lethal to a chronic, non‐lethal disease. The critical step in achieving this goal is the activation of tumor‐specific T cells to control the proliferation of cancer cells. Cancer vaccines are essential for activating tumor‐specific immunity to kill cancer cells. The main types of current cancer vaccines include peptides, mRNA, DNA, viral vectors, in vitro antigen‐loaded DC, and nanovaccines.^[^ ^1^, ^2^, ^3^, ^4^, ^5^, ^6^ ^]^ Provenge, an antigen‐loaded DC vaccine, is the only therapeutic cancer vaccine approved by the FDA.^[^ ^7^, ^8^ ^]^ Although approved for marketing, its efficacy is poor and can only prolong the lives of patients with prostate cancer by 3–4 months.^[^ ^7^, ^9^, ^10^ ^]^ To achieve better efficacy, many efforts have been made in recent years to develop neoantigen peptides and mRNA vaccines in recent years.^[^ ^8^, ^11^, ^12^, ^13^, ^14^, ^15^ ^]^ Neoantigen‐based cancer vaccines have some limitations, such as a time‐consuming and laborious preparation process (preparation takes 2–4 months), inability to overcome the high heterogeneity of cancer cells and tumor antigens, high costs, and relatively limited therapeutic efficacy.^[^ ^9^, ^10^, ^14^, ^16^ ^]^ Therefore, alternative cancer vaccines are urgently needed to improve the efficacy of cancer treatment.^[^ ^17^, ^18^, ^19^ ^]^ Cancer nanovaccine or micronvaccine loaded with whole tumor cell/tissue lysate is a promising cancer vaccine type, owing to the advantages of overcoming the high heterogeneity of cancer cells,^[^ ^17^, ^18^, ^19^, ^20^ ^]^ overcoming MHC (HLA) limitation,^[^ ^3^ ^]^ possessing a simpler preparation process (1–4 days to get the ready‐to‐use vaccine), lower cost, and affordability, especially by low‐income patients.^[^ ^7^, ^8^, ^10^, ^14^, ^21^ ^]^

In previous studies, we reported that nanovaccines loaded with whole tumor tissue/cell lysates (containing both tumor‐specific antigens (TSA) and tumor‐associated antigens (TAA)) could be utilized alone to treat melanoma and breast cancer, with a cure rate of ≈25%.^[^ ^3^ ^]^ However, the cure rate in our previous study was not good enough, and there is a significant untapped opportunity for such cancer vaccines. In preliminary studies, many critical parameters of cancer vaccines were not investigated or optimized, such as the collection methods of tumor tissues, their fixation, and purification of their lysates. Therefore, the therapeutic efficacy of preliminary cancer nanovacification is not satisfactory, and better cancer vaccines need to be explored to maximize the therapeutic efficacy of cancer vaccines.

In this study, the parameters that affect the efficacy of cancer vaccines were systematically optimized to maximize the efficacy of cancer nanovaccines (cure rate). These parameters include adjuvants, antigens (with their diversity and methods of collecting tumor tissues, lysis, fixation, purification, and oxidation of lysates), and formulation (size of vaccines and loading sites of antigens in particles). Among them, tumor antigens are the most important parameter.^[^ ^20^, ^22^, ^23^, ^24^ ^]^ These parameters are crucial for unleashing the potential of cancer vaccines, and thus, many conditions within these parameters have been explored to maximize the efficacy of cancer vaccines.^[^ ^25^, ^26^, ^27^, ^28^ ^]^

**Figure 1:**
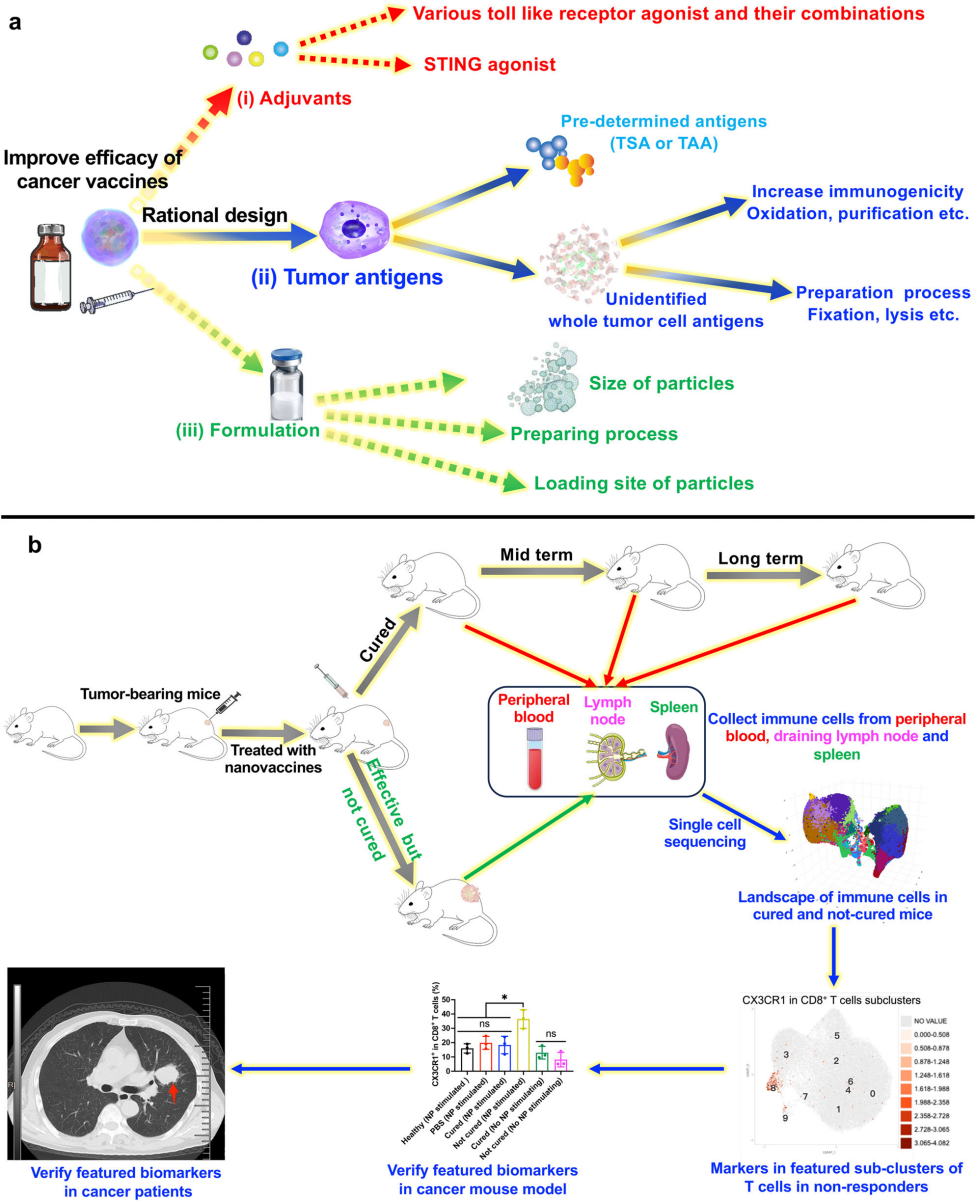
Schematic illustration of maximizing the efficacy of cancer vaccines by rational design and investigating the immune cell landscape of treated mice to explore featured markers of non‐responders to immunotherapy. a) Illustration of maximizing the efficacy of cancer vaccines by rational design and adjusting parameters to obtain optimal cancer vaccines loading whole tumor antigens. b) Analyzing immune cell profiles in the peripheral blood, lymph node, and spleen of tumor‐bearing mice treated with nanovaccines (cured vs uncured mice) to explore featured biomarkers in non‐responders to immunotherapy.

Herein, through rational design and exploration, the adjuvants, the best methods for collecting tumor tissues, the methods of lysing tumor tissues, the methods of fixating tumor tissues, the methods of purifying tumor tissue lysates (salting‐out, heating, and ethanol precipitation), oxidation methods of lysates (H_2_O_2_ and HClO), size of vaccines, and loading sites of antigens in particles were systematically explored and identified. In mouse models of melanoma, lung cancer, melanoma lung metastasis, and pancreatic cancer, optimized nanovaccines alone could cure 70–100% (completely no tumor) of tumor‐bearing mice (**Figure** **1**). Hence, utilizing appropriate processing methods for tumor tissues can dramatically improve the efficacy of cancer vaccines.

Understanding the differences between responders and non‐responders to immunotherapy is crucial.^[^ ^29^ ^]^ Therefore, the landscape of immune cell profiles of cured mice (freshly cured or post‐cured for 3, and 8 months and rechallenged with tumor inoculation) and uncured mice was systematically investigated and compared using single‐cell sequencing (Figure 1). The analyzed 21 samples from the blood, splenocytes, or draining lymph nodes (DLN) of cured or uncured mice had 28 clusters of different cell types. The diversity of CD8^+^ TCR, CD4^+^ TCR, and BCR in cured mice was much higher than that in uncured mice. In addition, CD8^+^ and CD4^+^ T cells were subjected to sub‐cluster analysis, and 10 CD8^+^ and 11 CD4^+^ T cell sub‐clusters were identified. In these clustering and sub‐clustering analyses, several sub‐clusters that existed only in non‐responders or responders to cancer vaccine treatment were identified. Interestingly, some markers of these critical T cell subtypes that are essential for distinguishing non‐responders to immunotherapy have been discovered. These markers included CX3CR1, A100A4, KLRG1, S1PR5, and S100A8 and are potentially promising biomarkers for distinguishing responders from non‐responders to immunotherapy. Therefore, several selected markers such as CX3CR1, A100A4, KLRG1, S1PR5, and S100A8 were investigated in cancer mouse models and patients with lung cancer to verify the feasibility of utilizing these markers to identify non‐responders and predict immunotherapy efficacy. The results demonstrated that changes in the expression of these identified markers could be used to distinguish non‐responders to immunotherapy.

## 2 Results

### 2.1 Preparation and Characterization of Nanovaccines (NVs) and Micron‐Vaccines (MVs)

**Figure 2:**
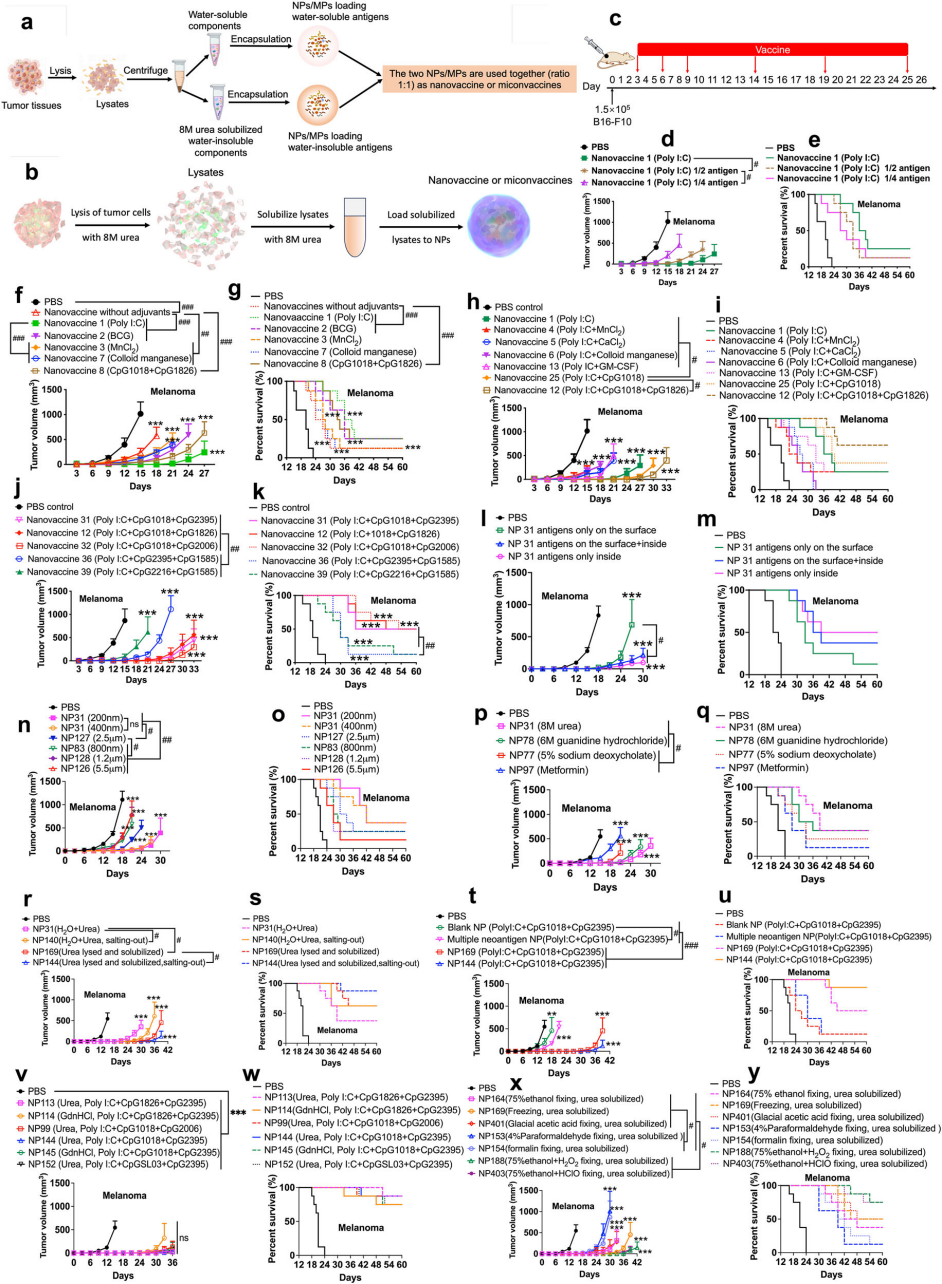
Optimization of cancer vaccines loaded with whole tumor antigens by altering adjuvants, antigen loading sites, sizes of vaccines, solubilization agents of water‐insoluble components, lysis methods of tumor tissues, antigen compositions, antigen purity, collecting methods of tumor tissues and oxidizing of antigens (*n*≥8). a) Lysis of tumor tissue by using repeated freezing–thawing in pure water, followed by loading both water‐soluble components and urea‐solubilized water‐insoluble components into nanovaccines or micron‐vaccines. b) Lysis of tumor tissue by using 8 m urea, followed by loading urea‐solubilized whole tissue components into nanovaccine or micron‐vaccines. c) Tumor inoculation of melanoma cells and treatment scheme of cancer vaccines. d,e) Tumor growth and survival curves in melanoma‐bearing mice treated with nanovaccines loading different amounts of antigens. f–k) Tumor growth and survival curves in melanoma‐bearing mice treated with nanovaccines loading different adjuvants. l,m) Tumor growth and survival curves in melanoma‐bearing mice treated with nanovaccines loading antigens at different sites. n,o) Tumor growth and survival curves in melanoma‐bearing mice treated with nanovaccines/micronvaccines with different sizes. p,q) Tumor growth and survival curves in melanoma‐bearing mice treated with nanovaccines loading different solubilizer solubilized antigens. r,s) Tumor growth and survival curves in melanoma‐bearing mice treated with nanovaccines loading purified tumor lysates. t,u) Tumor growth and survival curves in melanoma‐bearing mice treated with nanovaccines loading multiple neoantigens or tumor lysates. v,w) Tumor growth and survival curves in melanoma‐bearing mice treated with nanovaccines loading different adjuvants. x,y) Tumor growth and survival curves in melanoma‐bearing mice treated with nanovaccines loading tumor tissue lysates fixed with different fixation solutions. Data are presented as Mean ± SEM, Tumor growth over time was compared by two‐way ANOVA. Differences in survival were determined for each group by the Kaplan–Meier method, and the overall *p* value was calculated by the log‐rank test. *p*‐values < 0.05 were considered significant: ^**^ *p* < 0.01, ^***^ *p* < 0.005, ^#^ *p* < 0.05, ^##^ *p* < 0.01, ^###^ *p* < 0.005.

To re‐assemble tumor tissues into nano‐/micron‐sized vaccines, two different strategies were applied: 1) tumor tissues were lysed using repeated freezing–thawing in pure water, followed by loading the water‐soluble components or 8 m urea (or 6 m guanidine hydrochloride) solubilized water‐insoluble components into PLGA nanoparticles (NPs) or micron‐particles (MPs) respectively;^[^ ^13^, ^30^, ^31^, ^32^ ^]^ 2) tumor tissues were lysed with 8 m urea (or 6 m guanidine hydrochloride) and whole lysates were solubilized with 8 m urea (or 6 m guanidine hydrochloride), followed by loading solubilized lysates to PLGA NPs/MPs (**Figure** **2**a,b). Different sizes of NVs and MVs were prepared to compare the effect of size on efficacy. Furthermore, nanovaccines loaded with antigens at different sites were prepared to investigate the impact of the loading sites. In addition, to explore the effects of freezing, fixation, oxidation, salting‐out, ethanol precipitation, and heating, many NVs/MVs with different parameters were prepared. Detailed information on these NVs/MVs is listed in Table S1 (Supporting Information).

The sizes of the NVs were ≈200–800 nm and those of the MVs were ≈1.0, 2.5, and 5.0 µm (Figure S1, Supporting Information). Most of the NVs had sizes ranged from 200 to 300 nm. Both NVs and MVs were negatively charged (Figure S1, Supporting Information). The loading capacity of proteins/peptides in these NVs/MVs was ≈100 µg /1 mg PLGA. In morphological studies, both NV and MV had spherical structures, as observed by transmission electron microscopy (TEM) and confocal microscopy (Figure S2, Supporting Information).^[^ ^19^, ^20^, ^22^ ^]^ Both NV and MV can be efficiently taken up by APCs and release payloads in APCs (Figures S3–S5, Supporting Information).^[^ ^10^, ^18^, ^19^, ^33^, ^34^, ^35^, ^36^, ^37^ ^]^

### 2.2 Adjuvants can Affect the Therapeutic Efficacy of Nanovaccines

Adjuvants can amplify specific immune responses induced by tumor antigens in cancer vaccines; thus, adjuvants are an important factor affecting the efficacy of cancer vaccines. In order to activate tumor antigen‐specific T cells, 3 signals are needed in antigen‐presenting cells (APCs), especially dendritic cells (DCs). The first signal is a tumor antigen; the second signal is a co‐stimulating factor, such as CD28; and the third signal is a proinflammatory cytokine. The first signal is from tumor antigens, and the second and third signals are from adjuvants. Stronger signal 2 and signal 3 during the interaction between antigen peptides and T cell receptors (TCR) can bring better activation of tumor antigen‐specific T cells. Therefore, adjuvants can affect the efficacy of vaccines through providing the second signal and the third signal. Good adjuvants can activate signal 2 and signal 3 and thus assist tumor antigens to efficiently activate antigen‐specific T cells. In addition, ideal adjuvants should have receptors or targets located only or mostly in APCs (such as DCs and B cells), and this could minimize potential side effects. Based on these, several selected adjuvants were compared in our studies.

Co‐delivery of immune adjuvants and tumor antigens to APCs can activate tumor‐specific immune responses more efficiently than the delivery of tumor antigens alone.^[^ ^15^, ^25^ ^]^ Once NVs/MVs are taken up by APCs, adjuvants and antigens are released into the APCs. Antigens can provide signal one (peptide epitopes) and adjuvants can provide signals 2 (co‐stimulatory signals) and 3 (cytokines in the microenvironment) to activate tumor antigen‐specific T cells.

First, different amounts of tumor antigens in cancer vaccines can affect their therapeutic efficacy of cancer vaccines. Studies have shown that increasing the amount of whole tumor antigens in cancer vaccines can improve their efficacy of cancer vaccines (Figure 2a–c).

Next, poly(I:C) (which can stimulate TLR3), different CpG ODNs (types A, B, and C), GM‐CSF, Bacillus Calmette–Guérin (BCG), and STING agonists, or their combinations, were co‐encapsulated with tumor antigens into NVs/MVs. The therapeutic efficacy of NVs/MVs loaded with different adjuvants or their combinations was systematically compared in the treatment of melanoma‐bearing mice (Figure 2a). Theoretically, adjuvants targeting intracellular receptors perform better than those targeting extracellular receptors. This is because NVs/MVs are taken up by APCs, and adjuvants are released in APCs together with antigens; therefore, adjuvants can interact better with their corresponding intracellular than extracellular receptors.

A comparison of different single adjuvants showed that poly(I:C) and CpG ODNs performed better than STING agonists and BCG (Figure 2d,e). Combining poly(I:C) with GM‐CSF or STING agonists did not improve the efficacy of the vaccines compared to the use of poly(I:C) alone (Figure 2f,g). In addition, the combined application of poly(I:C) and CpG ODNs significantly improved the efficacy of vaccines compared to the use of a single adjuvant (Figure 2f,g). Moreover, the proper types of CpG ODNs in combined adjuvants are critical. CpG1018, CpG1826, and CpG2006 belong to type B CpGs; CpG2216 and CpG1585 belong to type A CpGs; and CpG 2395 and CpGSL03 belong to type C CpGs. The results demonstrated that one type B CpG + one type C CpC or one type B CpG + one type B CpC performed better than one type A CpG + one type A CpC or one type C CpG + one type A CpC (Figure 2h,i). This indicates that type C and B CpG ODNs are needed in combination with adjuvants. Altogether, these data indicate that different adjuvants can significantly affect the efficacy of cancer nanovaccines loaded with whole‐tumor antigens, and the combination of adjuvants poly(I:C) + two proper CpG ODNs performed best in the optimized formulations.

### 2.3 The Formulation and Size of Nanovaccines or Micronvaccines can Affect the Efficacy

We further analyzed the impact of vaccine formulation on the therapeutic efficacy of vaccines loaded with whole tumor antigens. The formulation can affect the uptake of cancer vaccines and the release of antigens from vaccines. For instance, loading antigens inside nanovaccines or on the surface of nanovaccines can significantly affect the release of antigens from nanovaccines. The size of nanovaccines or micronvaccines can also affect the uptake of cancer vaccines by APCs and the trafficking to LNs. For example, in our previous studies, we demonstrated that smaller nanoparticles can enter LNs directly by themselves. Whereas, micronparticles can not enter into LNs by themselves and need to be taken up by APCs, followed by APCs homing back to LNs. Therefore, size can affect the efficacy of cancer vaccines.

Tumor tissue lysates loaded on different nanovaccine sites were studied: coated on the surface alone, encapsulated inside the nanoparticles alone, and simultaneously coated on the surface and encapsulated inside the nanoparticles. The results demonstrated that encapsulation inside nanoparticles alone and simultaneous surface coating + encapsulation inside nanoparticles showed similar therapeutic efficacy (Figure 2j,k; Figure S6, Supporting Information). In addition, they are better than coating the surface alone.

Furthermore, the sizes of the nanovaccines and microvaccines were investigated (Figure 2l,m). 200 nm nanovaccines, 400 nm vaccines, 800 nm vaccines, 1.2 µm micronvaccines, 2.5 µm micronvaccines, and 5.5 µm micronvaccines were systematically investigated. The results indicated that 200 nm nanovaccines, 400 nm vaccines, and 2.5 µm micronvaccines showed similar therapeutic efficacy, and they are better than other‐sized vaccines. This result implied that <400 nm are optimal sizes for nanovaccines and ≈2.5 µm are the optimal sizes of micronvaccines to achieve ideal therapeutic efficacy.

### 2.4 Lysis Methods of Tumor Tissues can Affect Efficacy of Cancer Vaccines

Antigens are the most important factors affecting the efficacy of cancer vaccines, as tumor‐specific immune responses are induced by the antigens in vaccines. The processing, formation, and immunogenicity of antigens can significantly affect the efficacy of nanovaccines. In order to activate antigen‐specific T cells, tumor antigens need to be taken up by APCs, be processed in APCs, and then be presented on the surface of APCs. Different lysis methods can affect the confirmation of tumor antigens and thus affect the processing and presentation of tumor antigens in APCs. The processing and presentation of tumor antigens can affect the activation of antigen‐specific T cells, and thus affect the efficacy of cancer vaccines.

The processing of antigens can affect their formation and immunogenicity. Therefore, the procedure for preparing whole‐tumor antigens was investigated.

First, two different lysis methods for tumor tissues were compared, because the lysis of tumor tissues can affect the antigen composition and immunogenicity of tumor antigens. One lysis method utilizes ultrapure water to swell and burst tumor tissue cells, followed by repeated freeze‐thawing of the tumor tissue cells, together with ultrasonication to thoroughly lyse the cells. Tumor tissue lysates were centrifuged to collect the supernatant and precipitate. The supernatant was used as the water‐soluble component, and the precipitate was solubilized in solubilization solutions (8 m urea, or 6 m guanidine hydrochloride, sodium deoxycholate, or metformin) and utilized as solubilized water‐insoluble components. The second method involved lysing the tumor tissues with solubilization solutions (8 m urea or 6 m guanidine hydrochloride) and directly solubilizing the tumor tissues directly with 8 m urea or 6 m guanidine hydrochloride (GdnHCl).

Tumor antigen processing can significantly affect the antigen composition and immunogenicity of tumor antigens. Therefore, the efficacy of cancer nanovaccines prepared from tumor tissue lysates obtained by the above two methods was investigated in terms of their therapeutic efficacy (Figure 2n–p). The results indicated that 8 m urea and 6 m guanidine hydrochloride were better than sodium deoxycholate and metformin in terms of the therapeutic efficacy of the prepared vaccines after solubilizing water‐insoluble components (Figure 2n,o). In addition, lysing tumor tissues with 8 m urea or 6 m guanidine hydrochloride was slightly better than lysing tumor tissues with ultrapure water and repeated freeze‐thaw cycles (Figure 2p,q). This is probably because the lysis and solubilization of tumor tissues with 8 m urea or 6 m guanidine hydrochloride can more rapidly denature all proteins and thus reduce the interactions between different proteins, which decreases the chances of degradation of antigen proteins.

### 2.5 Antigen Compositions and Antigen Purity Can Affect Efficacy of Cancer Vaccines

Antigen composition and purity can significantly affect the efficacy of cancer vaccines. Both more diverse tumor antigens and purer tumor antigens can improve the efficacy of cancer vaccines. Therefore, antigen composition, including only adjuvants, multiple melanoma neoantigens, whole tumor lysates, and purified whole tumor lysates, was systematically investigated (Figure 2r,s).

As shown in Figure 2, NPs loaded with whole‐tumor lysates and purified whole‐tumor lysates showed better therapeutic efficacy than blank NPs loaded with only adjuvants and NPs loaded with multiple neoantigens. These results demonstrated that, owing to including a broad range of tumor antigens, whole tumor antigens showed better efficacy than multiple neoantigens. Moreover, including more diverse tumor antigens in cancer vaccines is better for overcoming the high heterogeneity of cancer cells and tumor antigens.

Furthermore, NPs loaded with purified whole‐tumor lysates showed better therapeutic efficacy than those loaded with non‐purified whole‐tumor lysates. This is because utilizing salting‐out to purify whole tumor antigens can remove lipids and sugars to concentrate protein/peptide antigens.

To further verify whether the combination of type B and C CpGs can achieve similar efficacy, different combinations of poly (I:C)+CpGs were studied. As shown in Figure 2t,u, all adjuvant combinations showed potent therapeutic efficacy, and most melanoma‐bearing mice were cured. In addition, urea and GdnHCl showed similar efficacies in lysing and solubilizing tumor tissues for the preparation of cancer vaccines.

### 2.6 Collecting Methods of Tumor Tissues Can Affect Immunogenicity of Tumor Antigens

The collection of tumor tissues can affect the degradation of antigen proteins, thus affecting the amount of antigen proteins and immunogenicity. When we collect tumor tissues, tumor cells are still alive and tumor cells may secrete proteins/enzymes. For instance, if the tumor tissues are put at room temperature for too long time, the autophagy and the degradation of some proteins will happen. The autophagy and the degradation of some proteins can affect the amount of tumor antigens in tumor tissues, and thus affect the efficacy of cancer vaccines. Freezing or fixing tumor tissue immediately after the tumor tissues are removed from tumor‐bearing bodies can help to keep the original state and protein profiles in tumor tissues, and thus affect the efficacy of cancer vaccines.

Therefore, different methods for collecting tumor tissues were compared, including freezing tumor tissue directly after surgery, fixing tumor tissue with different types of fixation solutions, and fixing tumor tissue with fixation solutions plus oxidizing agents (HClO or H_2_O_2_) (Figure 2v,w). Fixation solutions included formalin, 4% paraformaldehyde, glacial acetic acid, and 75% ethanol. The fixation solutions and oxidizing agents included 75% ethanol with hypochlorous acid (200 nm HClO) and 75% ethanol with hydrogen peroxide (H_2_O_2_, 1%). The results indicated that antigen processing can significantly affect efficacy, and freezing tumor tissue directly after surgery or fixing tumor tissue with 75% ethanol showed better therapeutic efficacy than fixing the tumor tissue with formalin, glacial acetic acid, or 4% paraformaldehyde. In addition, oxidizing tumor antigens with HClO or H_2_O_2_ during collection can improve the therapeutic efficacy of prepared cancer vaccines. Moreover, HClO and H_2_O_2_ have similar effects in improving therapeutic efficacy. These data illustrate that freezing tumors immediately after surgery or fixing tumor tissues with a proper fixation solution can protect antigen proteins from degradation, and thus improve therapeutic efficacy by preserving more antigen proteins during the processing of tumor tissues. Oxidizing tumor antigens with HClO or H_2_O_2_ during the collection process improved therapeutic efficacy, probably because oxidizing antigen proteins elevated the immunogenicity of antigen epitopes in such proteins.

### 2.7 Purifications of Whole Tumor Lysates Can Affect Efficacy of Cancer Vaccines

**Figure 3:**
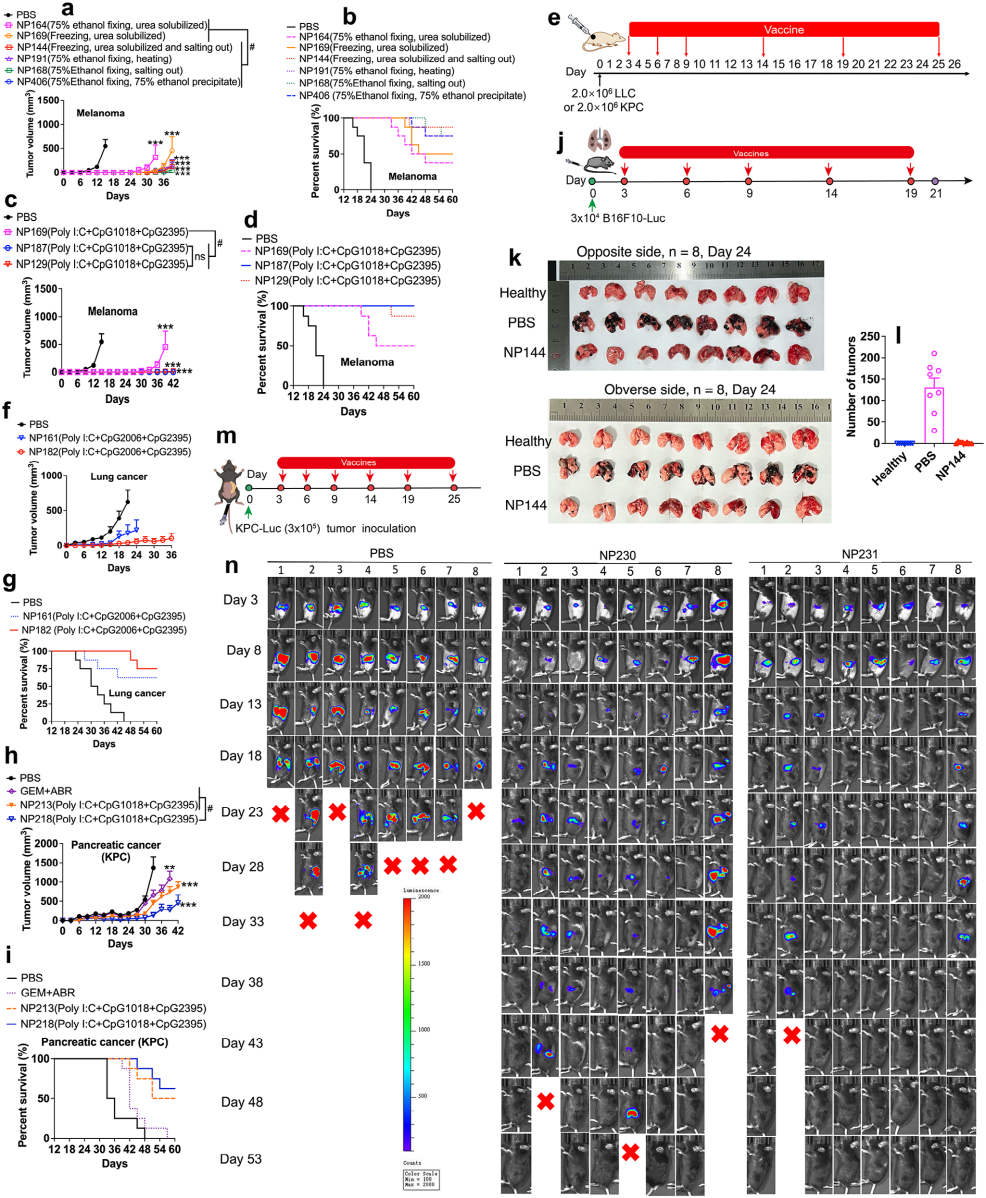
Optimized cancer nanovaccines can efficiently treat cancers in multiple mouse models. a,b) Tumor growth curves and survival curves in melanoma‐bearing mice treated with nanovaccines loading tumor tissue lysates purified with different methods. c,d) Tumor growth curves and survival curves in melanoma‐bearing mice treated with nanovaccines loading oxidized tumor tissue lysates(187: H_2_O_2_; 129:HClO). e) Tumor inoculation of lung cancer cells or pancreatic cancer cells and the treatment scheme. f,g) Tumor growth curves and survival curves in lung cancer‐bearing mice treated with nanovaccines. h,i) Tumor growth curves and survival curves in pancreatic cancer‐bearing mice treated with nanovaccines. j) Tumor inoculation of melanoma lung metastasis and treatment scheme. k,l) Results of melanoma lung metastasis treated with nanovaccines (mice were sacrificed on day 24). m) Tumor inoculation of orthotopic pancreatic cancer cells and treatment scheme. n) Monitoring tumor growth in orthotopic pancreatic cancer model treated with nanovaccine 230 and nanovaccine 231 using in vivo imaging. Data are presented as Mean ± SEM. Tumor growth over time was compared by two‐way ANOVA. Differences in survival were determined for each group by the Kaplan–Meier method, and the overall *p* value was calculated by the log‐rank test. *p*‐values < 0.05 were considered significant: ^**^ *p* < 0.01, ^***^ *p* < 0.005, ^#^ *p* < 0.05.

As demonstrated in the above experiments, improving the purity of tumor antigens can improve the efficacy of vaccines. Therefore, different purification methods for whole tumor antigens were compared during the preparation of cancer nanovaccines (**Figure** **3**a,b). Whole tumor tissue lysates, whole tumor lysates purified by salting‐out, whole tumor lysates purified by salting‐out + heating, and whole tumor lysates purified by 75% ethanol precipitation were loaded into nanovaccines to evaluate their therapeutic efficacy. The results indicated that purifying whole tumor lysates with salting‐out, salting‐out + heating, and 75% ethanol precipitation can improve the therapeutic efficacy of nanovaccines owing to the removal of sugars and lipids and the enrichment of antigen proteins by such a purification process.

### 2.8 Oxidation of Whole Tumor Lysates Can Affect Efficacy of Cancer Vaccines

As demonstrated by the collection methods, oxidizing tumor antigens during the collection process can improve their immunogenicity of tumor antigens. To further verify that oxidizing tumor antigens after collection can also improve the therapeutic efficacy of vaccines, HClO and H_2_O_2_ were used to oxidize tumor antigens after collecting the tumor tissues (Figure 3c,d). These results verified that oxidizing tumor antigens with HClO (NP129) or H_2_O_2_ (NP187) after collecting tumor tissues could improve the therapeutic efficacy of nanovaccines.

### 2.9 Optimized Nanovaccines Can Efficiently Treat Lung Cancer and Subcutaneous Pancreatic Cancer

As shown above, the optimized nanovaccines cured all melanoma‐bearing mice. To verify the efficacy of optimized nanovaccines in multiple cancer models, nanovaccines were further tested in mouse models of lung cancer (LLC), subcutaneous pancreatic cancer (KPC), melanoma lung metastasis (B16F10), and orthotopic pancreatic cancer (KPC).

As shown in Figure 3e–g, such nanovaccines could efficiently treat lung cancer, and most of the tumor‐bearing mice were cured. In addition, nanovaccines (NP 182) loaded with purified whole tumor antigens performed better than nanovaccine‐loaded non‐purified whole tumor lysates (NP 161).

A similar trend was observed in subcutaneous pancreatic cancers (NP218 and NP 213), which is one of the most malignant and aggressive cancers. More importantly, the nanovaccines (NP218 and NP 213) showed much better therapeutic efficacy than the clinically used chemotherapy method (Figure 3e,h,i). These results demonstrated that, in mouse models, optimized cancer nanovaccines alone can cure most pancreatic cancer‐bearing mice.

### 2.10 Optimized Nanovaccines Can Efficiently Treat Melanoma Lung Metastasis

Clinically, most patients with cancer die because of metastasis or recurrence. Therefore, the efficacy of nanovaccines in treating melanoma metastasis was investigated using a mouse model. Studies conducted on melanoma lung metastasis showed that nanovaccines (NP144) could efficiently treat melanoma metastasis in the lungs and cure most mice (Figure 3j,l). These results confirmed that our optimized cancer nanovaccines can efficiently treat cancer metastasis.

### 2.11 Optimized Nanovaccines Can Efficiently Treat Orthotopic Pancreatic Cancer

Pancreatic cancer is one of the most malignant and aggressive cancers, and the efficacy of nanovaccines in orthotopic pancreatic cancer is investigated in our studies (Figure 3m,n). The results demonstrate that nanovaccines (NP230 or NP231) could cure most mice with orthotopic pancreatic cancer. In addition, oxidizing whole tumor antigens with H_2_O_2_ (NP231) improved the efficacy of whole tumor antigens (NP230).

Rechallenge studies showed that all these cured mice are resistant to re‐inoculation of tumor cells, and all mice become tumor‐free after being rechallenged with pancreatic cancer cells on day 60.

Taken together, these therapeutic studies conducted on multiple cancer models further confirmed that optimized cancer vaccines could cure most tumor‐bearing bodies, even orthotopic pancreatic cancer.

### 2.12 No Toxicity was Observed in Mice Treated with NVs

In all the above therapeutic studies conducted with mice, the body weights of mice in both the MVs‐ and NV‐treated groups did not decrease significantly (Figure S7, Supporting Information). In addition, neither MVs and NVs NV‐immunized mice showed any abnormality in the H&E study conducted on samples of the heart, liver, spleen, lung, and kidney from vaccine‐treated mice (Figure S8, Supporting Information). These results indicated that the NVs and MVs did not cause any toxicity to the major organs.

More importantly, critical inflammatory factors in the blood were investigated using ELISA to evaluate their potential for inducing a cytokine storm. The results indicated that no significant increase in inflammatory factors was observed in the blood of vaccine‐treated mice. These data further confirm that there is no potential risk of cytokine storm syndrome (Figure S9, Supporting Information).

Analysis of biochemical indices in the blood of vaccine‐treated mice indicated that no significant changes were observed in the nanovaccine‐treated mice, verifying that no toxicity was induced by the nanovaccine treatment (Figure S10, Supporting Information).

All these toxicity studies demonstrated that these nanovaccines are safe, and treating mice with the presented nanovaccines showed no toxicity.

### 2.13 T Cell Receptors (TCR) and B Cell Receptors (BCR) Diversities are Higher in Cured Mice

One advantage of nanovaccines loaded with whole tumor antigens is the induction of a broad range of tumor antigen‐specific T cell responses. Therefore, the diversity of TCR and BCR in different cured mouse groups (different periods after curing from tumor inoculation) and uncured mice (tumor‐bearing) were systematically investigated. The un‐cured group was the non‐responder group, and the cured groups were the responders. In total, 21 different immune cell samples from the peripheral blood, DLN, and splenocytes from seven different groups were analyzed using TCR and BCR sequencing. These seven different groups include: 1), healthy mice (healthy group, samples 1, 8, 15); 2), B16F10 tumor‐bearing mice treated with PBS (PBS group, samples 2, 9, 16); 3), B16F10 tumor‐bearing mice treated with nanovaccines but not cured (treated but not cured group, samples 3, 10, 17); 4), B16F10 tumor‐bearing mice treated with nanovaccines and freshly cured (treated freshly cured group, samples 4, 11, 18); 5), B16F10 tumor‐bearing mice treated with nanovaccines and cured for 3 months (treated cured 4 months group, samples 5, 12, 19); 6) B16F10 tumor‐bearing mice treated with nanovaccines and cured for 7 months (treated cured 7 months and vaccine administered group, samples 6, 13, 20); 7) B16F10 tumor‐bearing mice cured for 7 months and rechallenged with B16F10 tumor inoculation 3 days ahead of sacrifice (treated cured 7 months and cancer rechallenged group, samples 7, 14, 21).

Because many different gene sequences can encode the same peptide sequence, TCR or BCR gene sequences were correlated to the corresponding peptide sequences to avoid incorrect calculations. Therefore, the diversities obtained in this study are the diversities of peptide sequences, not those of gene sequences, which are much more accurate.

**Figure 4:**
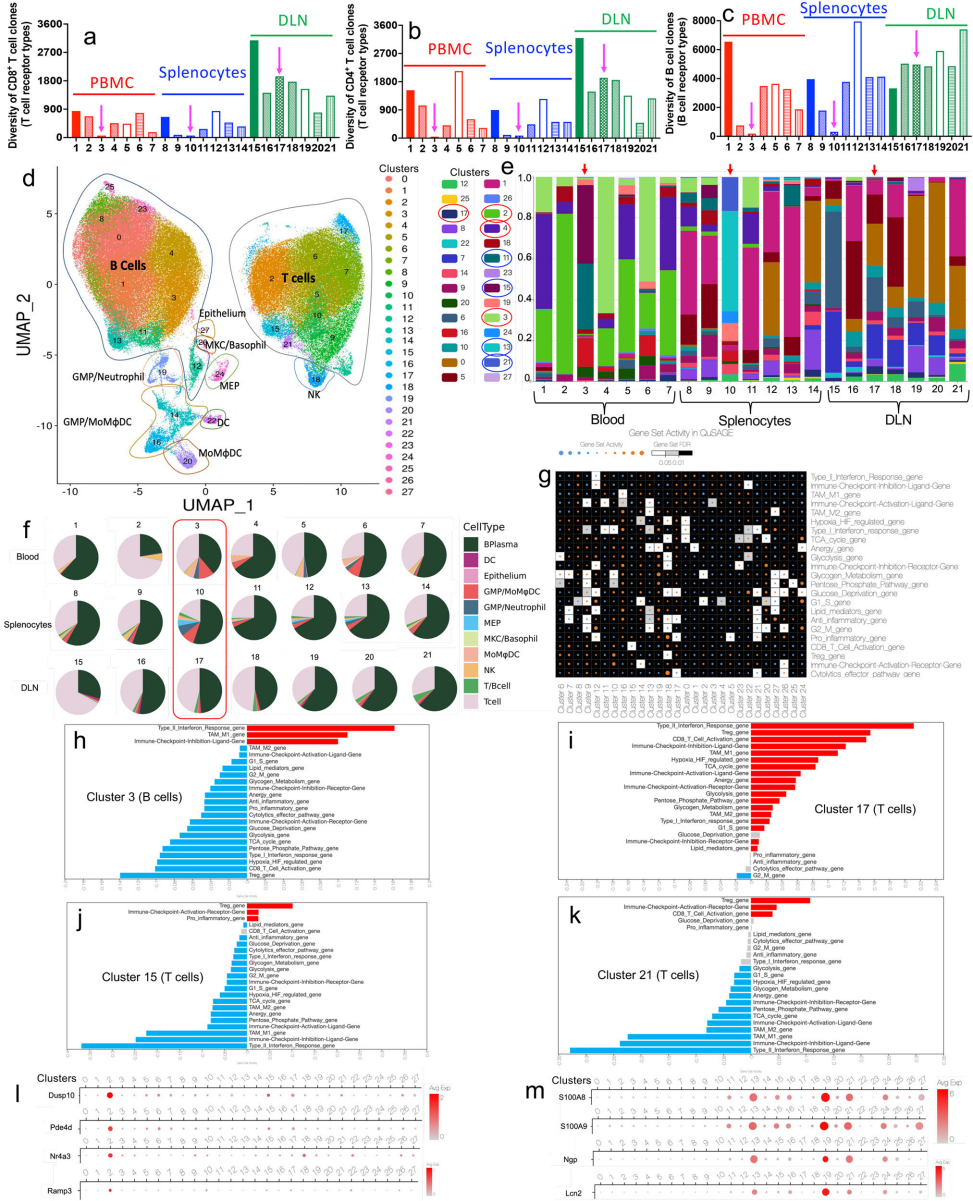
Landscape of TCR/BCR diversities and immune cell clusters in the blood (samples 1–7), splenocytes (samples 8–14) and DLN (samples 15–21) samples from healthy (samples 1, 8, 15), PBS treated tumor‐bearing (samples 2, 9, 16), uncured (samples 3, 10, 17), (tumor‐bearing), and different cured (cured after different periods) mice. a) Analysis of diversities of CD8^+^ T cell clones in 21 groups by single‐cell TCR sequencing. b) Analysis of diversities of CD4^+^ T cell clones in 21 groups by single‐cell TCR sequencing. c) Analysis of the diversities of B cell clones in 21 groups by single cell BCR sequencing. d) 28 clusters in immune cells samples from the blood, splenocytes, and DLN. e) The proportions of clusters in 21 groups from the blood, splenocytes, and DLN. f) Proportions of different cell types in 21 groups from the blood, splenocytes, and DLN. g) Gene set activity of 28 clusters. h–k) Highly expressed gene sets in clusters 3, 17, 15 and 21. l,m) Expressions of several selected featured markers in 28 clusters.

Compared with peripheral blood mononuclear cells (PBMC), the DLN and splenocyte samples from the healthy mice group, the diversity of CD8^+^ TCR, CD4^+^ TCR, and BCR decreased in the PBS, un‐cured, and differently cured groups (**Figure** **4**a–c). This is due to the burden on tumor cells that stimulate and expand tumor‐specific T cell clones in mice. In addition, mice from the PBS group had more diverse CD8^+^ TCR, CD4^+^ TCR, and BCR than those from the most cured and un‐cured groups. This was due to the increase in some tumor‐specific T cell clones induced by tumor cells and cancer vaccines, causing a decrease in detectable diversity in these samples. These results proved that the vaccine treatment induced the expansion of tumor‐specific T cell clones.

More interestingly, the results demonstrated that the diversity of CD8^+^ TCR, CD4^+^ TCR, and BCR in PBMC and splenocytes of different cured groups was much higher than that in the un‐cured group. In the DLN samples, no significant differences were observed between cured and un‐cured mice in the diversity of CD8^+^ TCR, CD4^+^ TCR, and BCR. These results indicate that un‐cured mice have fewer types of TCR and BCR in the blood and spleen, and this decrease in diversity is probably due to the increase in only a few dominant TCR, which limits the capacity to recognize more diverse antigens in tumor cells.

These results illustrate that cancer vaccines induced more tumor antigen‐specific T/B cell clones in cured mice. This phenomenon correlates with the therapeutic results: more diverse tumor antigen‐specific T cell responses are stimulated in the responders, thus inducing the curing of such tumor‐bearing bodies.

### 2.14 General Immune Cell Differences Between Cured and Un‐Cured Mice

Currently, only some patients with cancer are responsive to immunotherapy (such as αPD‐1 antibody), and most patients are non‐responsive to immunotherapy. Thus, it is critical to explore the differences between responders and non‐responders and to determine the underlying mechanisms. Therefore, we systematically investigated the differences between the cured and uncured groups after immunotherapy. As described above, the healthy PBS group, treated but not cured, and four different cured groups were compared. In total, 21 different immune cell samples from the peripheral blood, DLN, and splenocytes of mice in different groups were analyzed using single‐cell sequencing.

The preliminary clustering analysis results showed that there were 28 cell subpopulations in the PBMC, DLN, and splenocytes (Figure 4d; Figures S11 and S12, Supporting Information). Clusters 0, 1, 3, 4, 8, 11, 13, 23, and 25 were B cells, whereas clusters 2, 5, 6, 7, 9, 10, 15, 17, and 21 were T cells. Cluster 12 belonged to proliferative B/T cells, cluster 18 belonged to NK cells, and clusters 14, 16, 19, 20, 22, 24, 26, and 27 belonged to dendritic cells (DC), macrophages, and monocytes.

The landscape of immune cell profiles in PBMC, splenocytes, and DLN is shown in Figure 4e. The results indicated that immune cells from the healthy, freshly cured, cured for a period of time 3 months, cured for a long time 7 months, and cured for a long time 7 months with cancer cell rechallenge groups had similar profiles, either in B cells or in T cells (Figure 4e,f). This indicated that the immune profiles of nanovaccine‐cured mice can last for a long time. The immune cell profiles in the treated but un‐cured group or in the PBS group are significantly different from those in the healthy group and in various cured groups. Additionally, immune cells from PBMC and splenocytes showed similar patterns of cell subtype distribution. Immune cells from cured and un‐cured groups showed significant differences in T cells.

Given that cured groups indicate responders to immunotherapy and un‐cured groups indicate non‐responders to immunotherapy, features in cured or un‐cured groups could be utilized to differentiate responders and non‐responders to immunotherapy. Taking immune cells from PBMC as an example, in lymphocytes, the healthy group or different cured groups had more B cells than the PBS group or the treated but un‐cured group (Figure 4e,f).

In terms of B cells, the healthy group and different cured groups had more B cells in clusters 3 and 4 than the PBS group and the treated but un‐cured group (Figure 4e). The PBS group and the treated but un‐cured group had more B cells in clusters 13 and 11 than the healthy group and different cured groups. These results indicated that cluster 11 (B cells) and cluster 13 (B cells) are feature clusters in the treated but not cured group, due to cluster 13 only existing in this group, and cluster 11 in this group is ten times higher than that in other groups. In addition, cluster 4 (B cells) and cluster 8 (B cells) are the featured clusters in the healthy and cured groups, due to cluster 8 only existed in these groups, and the amount of cluster 4 is much higher in these groups. Similar cell features and differences were also observed in splenocyte samples, showing that similar immune cell profiles were present in PBMC and splenocytes.

In terms of T cells, the healthy group, different cured groups, and the PBS group had more T cells in clusters 2, 10, and 17 than the treated but not cured group (Figure 4e). Meanwhile, the treated but uncured group had more T cells in clusters 15 and 21 than the other groups. Cluster 15 (T cells) and cluster 21 (T cells) were feature clusters in the treated but not cured group, due to cluster 21 only existing in this group, and cluster 15 in this group is ten times higher than other groups. Cluster 17 (T cells) is the featured cluster in the healthy and cured groups, duo to cluster 17 only existed in these groups. Similar differences in clusters were also observed in splenocyte samples, demonstrating that similar immune cell profiles were present in PBMC and splenocytes.

Gene expression and function analysis of featured clusters demonstrated that the featured clusters of the un‐cured group (clusters 11, 13, 15, and 21) showed higher expression of the type 1 interferon response, anergy, T_reg_, and checkpoint activation genes, which is adverse to killing cancer cells (Figure 4g–k; Figures S13–S16, Supporting Information). The featured clusters of the cured groups showed higher expression of type 2 interferon response, checkpoint inhibition, and CD8^+^ T cell activation genes, which is beneficial for killing cancer cells (Figure 4g–k; Figures S13 and S14, Supporting Information).

The unique characteristic cell markers in subgroups 2, 3, 4, 8, 10, 17, 11, 13, 15, and 21 can be used to distinguish between the cured (responders) and un‐cured groups (non‐responders) (**Table** **1**). Figure 4l,m showed some genes that were highly expressed in clusters 2, 11, 13, and 21.

**Table 1:** Featured genes in clusters of T cells or B cells.

Clusters in T cells or B cells	Featured genes
Cluster 3 (B cells)	CD22, CD79b, CD24a, EIF2AK3, BLK, POU2af1, MEF2C, BMP2K, SNX30, FCRLA, DMXL1, TNFRSF1, FCMR, LGHD, FCER2A H2‐DMB2, MS4A1, and LITAF
Cluster 8 (B cells)	CD22, CD79b, FCRL1, H2‐Oa, SWAp70, CiiTa, PXDC1, Pold4, SNN, CXCR5, and zfp318
Cluster 2 (T cells)	SATB1, NR4A3, RAMP3, PLCXD2, PDE4D, DUSP10, EMB, RNF19A, DBF4, PJA1, and TIPRL
Cluster 10 (T cells)	TNFRSF4, ICOS, IL2RA (CD25), CTLA4, LKZF2, RORA, and CISH. TNFRSF4, and ICOS
Cluster 17 (T cells)	CXCR5, CD79b, CD22, CD24a, CD55 (decay accelerating factor, DAF), CD72, CD38, CiiTa, BANK1, CR2, TNFRSF13C, SWAP70, BC11a, SPiB, FCRLA, FCRL1, GGA2, H2‐Oa, BLK, SNN, PAX5, Ly6D, SCD1, PML, ZFP38, PJA1, TECPR, GALNT6, EXT1, EXT1 (exosin glycyltransfer 1), and GM20186
Clusters 11 (B cells), 13 (B cells), 15 (T cells), and 21 (T cells)	S100A8, S100A9, RETnLG, Lyz2, and Camk2d
Clusters 11 and 15	CXCL2
Clusters 13 and 21	NGP, Ltf, and Chil3
Clusters 11, 13 and 15	IL 1b
Clusters 13,15,21	LCN2

As shown in Table 1, highly expressed genes in cluster 3 (B cells) or cluster 8 (B cells) included CD22, CD79b, and EIF2AK3, etc. CD22 is a regulatory molecule that prevents the overactivation of the immune system and the development of autoimmune diseases.^[^ ^38^ ^]^ CD79b and CD24a can function in activating B cells. EIF2AK3 plays a role in responding to ER stress.^[^ ^39^ ^]^

Genes that were highly expressed in cluster 2 (T cells) included SATB1 and NR4A3, etc., which indicates the functions related to cytotoxicity and cell proliferation. SATB1 plays a role in T cell development, and the high expression of SATB1 can increase the amount of CD8^+^ T cells and decrease the amount of T_reg_ subsets.^[^ ^40^ ^]^ NR4A3 may act as a transcriptional activator and plays a central regulatory role in cell proliferation, differentiation, mitochondrial respiration, and metabolism. In cluster 10 (T cells), TNFRSF4 and ICOS, etc., are highly expressed and they are markers of activated T cells, indicating these T cells are activated by antigens and can play cytotoxic functions.^[^ ^41^ ^]^ In cluster 17 (T cells), the expressions of markers such as CXCR5 are elevated. CXCR5 is often defined as a marker for T Follicular Helper (Tfh) cells.^[^ ^42^ ^]^ The markers in these clusters illustrated the enhancement of cytotoxic T cell functions.

Genes S100A8 and S100A9, etc., increased in clusters 11, 13, 15, and 21. The proteins S100A8 and S100A9 form a heterodimer called calprotectin, that is elevated in persons with chronic inflammation.^[^ ^43^ ^]^ The prothrombotic pathway initiated by interaction of S100A8/A9 with GPIbα induces the formation of procoagulant platelets and fibrin (CD36 has a supporting role).^[^ ^44^ ^]^ The characteristic gene in clusters 11 and 15 was CXCL2, which is involved in many immune responses including wound healing, cancer metastasis, and angiogenesis.^[^ ^45^, ^46^ ^]^ CXCL2 in combination with the CXCR4 inhibitor plerixafor rapidly mobilizes hematopoietic stem cells into the peripheral blood.^[^ ^46^ ^]^ Clusters 13 and 21 have characteristic genes NGP, Ltf, and Chil3, which play important roles in inflammation and allergy. The characteristic gene in clusters 11, 13, and 15 was IL1b, which is an important mediator of the inflammatory response and a variety of cellular activities. Increased production of IL‐1b causes a number of different autoinflammatory syndromes.^[^ ^47^ ^]^ The characteristic gene in clusters 13, 15, and 21 was LCN2, which is involved in innate immunity.^[^ ^48^ ^]^

These characteristic genes in PBMC or splenocytes could be potential markers for distinguishing between responsive and non‐responsive groups after immunotherapy.

### 2.15 Exploring Featured CD8^+^ and CD4^+^ T Cell Subtypes using Sub‐Clustering Analysis

Further sub‐cluster analyses were performed on B and T cells. There were 15 sub‐clusters of B cells. T cells can be classified into two categories: CD8^+^ and CD4^+^ T cells. Sub‐cluster analysis was performed on CD8^+^ and CD4^+^ T cells, resulting in 10 and 11 sub‐clusters of CD8^+^ and CD4^+^ T cells, respectively.

By investigating 15 sub‐clusters in B cells, no featured sub‐clusters were found in these B cell sub‐clusters between the un‐cured and all cured groups.

**Figure 5:**
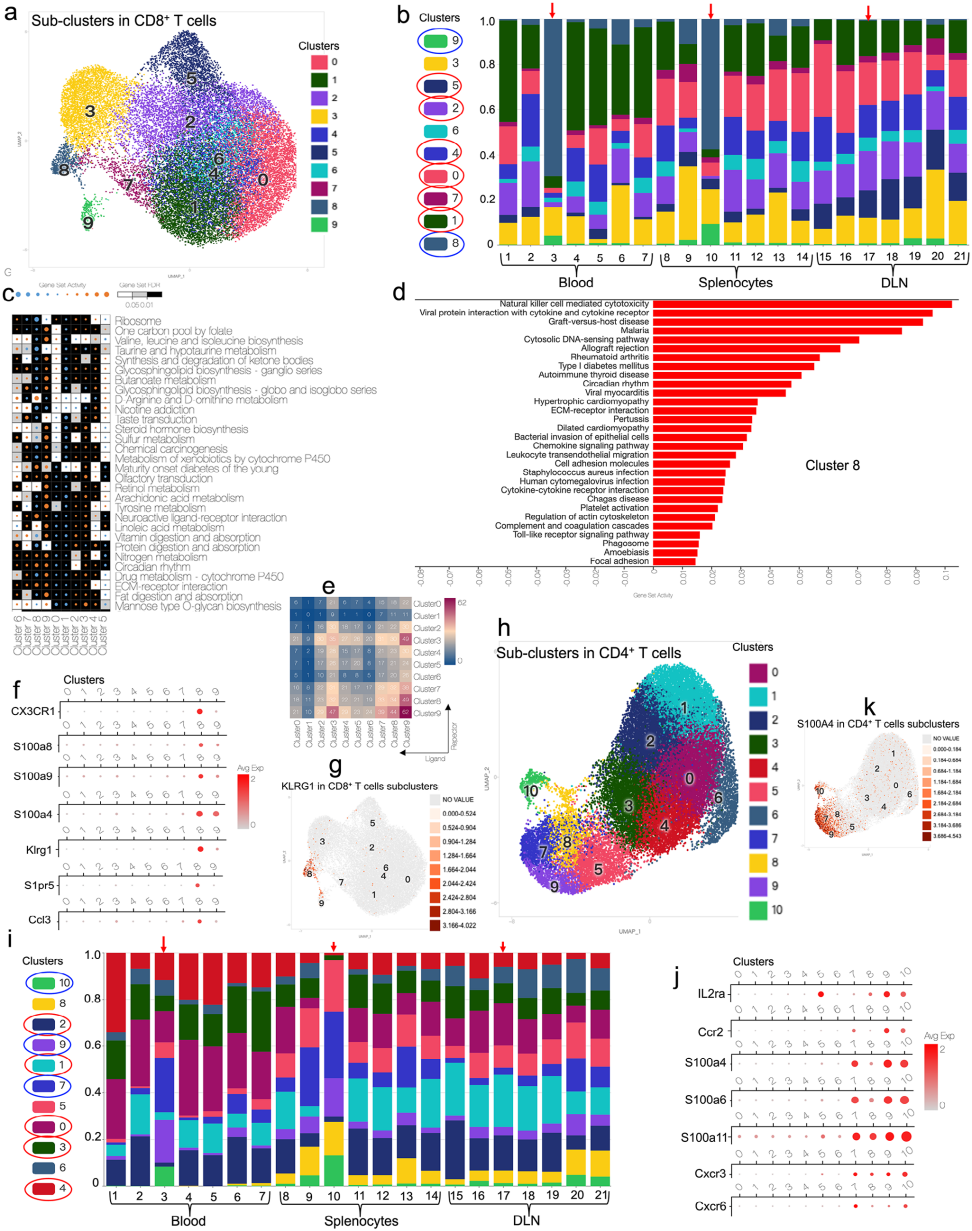
Landscape of sub‐clusters in CD8^+^ and CD4^+^ T cells in responders and non‐responders. a) Ten sub‐clusters in CD8^+^ T cells. b) Proportions of CD8^+^ T cells sub‐clusters in 21 groups from the blood, splenocytes, and DLN. c) Gene set activity of 10 CD8^+^ T cells sub‐clusters. d) Highly expressed gene set in cluster 8 of CD8^+^ T cells. e) Cell communications among 10 sub‐clusters in CD8^+^ T cells. f) Expression of several selected featured markers in 10 sub‐clusters of CD8^+^ T cells. g) Expression of CX3CR1 in 10 sub‐clusters of CD8^+^ T cells. h) Eleven sub‐clusters in CD4^+^ T cells. i) The proportions of CD4^+^ T cells sub‐clusters in 21 groups from the blood, splenocytes, and DLN. j) Expressions of several selected featured markers in 11 sub‐clusters of CD4^+^ T cells. k) Expression of S100A4 in 11 sub‐clusters of CD4^+^ T cells.

The analysis of 10 sub‐clusters of CD8^+^ T cells in the peripheral blood and splenocytes revealed significant differences between the four cured groups (responders) and the un‐cured group (non‐responders) (**Figure** **5**a). Taking CD8^+^ T cells in the peripheral blood as an example, the number of CD8^+^ T cells in sub‐clusters 0, 1, 2, 4, 5, and 7 are higher in the healthy and cured groups; the number of CD8^+^ T cells in sub‐clusters 8 and 9 significantly increased in the treated but not cured group (Figure 5b). In peripheral splenocytes, the number of CD8^+^ T cells in sub‐clusters 0, 1, 2, 4, and 6 increased in the healthy and cured groups, and the number of CD8^+^ T cells in sub‐clusters 8 and 9 significantly increased in the un‐cured group. Therefore, the above‐mentioned sub‐clusters in PBMC and splenocytes can be used to evaluate the efficacy of immunotherapy.

Gene expression and function analyses indicated that featured CD8^+^ T cell sub‐clusters in the un‐cured group had higher expression of NK cell cytotoxicity and autoimmune disease‐related genes (Figure 5c–e; Figures S15 and S16, Supporting Information). Whereas, featured CD8^+^ T cell sub‐clusters in the cured groups showed higher expression of genes related to ribosomes and T‐cell receptor signaling. Sub‐clusters 8 and 9 are regulatory T cells (T_reg_) and exhausted T cells. Sub‐clusters 2, 4 and 5 are cytotoxic T cells. These indicated that CD8^+^ T cells in the healthy and cured groups have more cytotoxic T cells, while CD8^+^ T cells in un‐cured group have more regulatory T cells and exhausted T cells.

The characteristic markers in cluster 8 included KLRG1, S100A4, CX3CR1, S1PR5, S100A8, and S100A9 etc. (Figure 5f,g; Figure S17, Supporting Information). KLRG1 is a lymphocyte co‐inhibitory, or immune checkpoint, receptor expressed predominantly on late‐differentiated effector and memory CD8^+^ T and NK cells. Besides as a co‐inhibitory marker, KLRG1 could be also a “senescent” marker or “exhaustion” markers for T cells.^[^ ^49^, ^50^, ^51^, ^52^ ^]^ S100A4 is potentially associated with angiogenesis and cancer metastasis.^[^ ^53^, ^54^, ^55^ ^]^ CX3CR1 is expressed in inflammatory response in T cells and NK etc.,^[^ ^56^ ^]^ and it is associated with the metabotropic function and modulating cell activity toward higher active state.^[^ ^57^ ^]^ S1PR5 is a migratory receptor with an uncharted function in T cells. S1PR5 plays a critical role in T cell infiltration and emigration from peripheral organs, as well as being specifically downregulated in tissue‐resident memory T cells (T_RM_). T_RM_ development was selectively impaired upon ectopic expression of S1PR5, whereas loss of S1PR5 can enhance the formation of T_RM_ by promoting peripheral T cell sequestration.^[^ ^58^ ^]^ The increased expression of these above markers indicated the inhibition of cytotoxic function of T cells.

Characteristic markers in sub‐cluster 9 included CDCA8, RRM1, KIF5, PIK1, PCLAF, CDK2a, CENPa, and RBBp8 etc. Elevated genes in both sub‐clusters 8 and 9 included KLRA7, KLRA1, KLRE1, Myb, CCL5, CTLA2a, and CCR5, etc. Increased genes in sub‐clusters 7 and 9 included CTLA4 and Myb. The characteristic markers in sub‐cluster 5 included EIF2AK2, DDX60, RSAD2, and LFIH1, etc. Detailed information is provided in **Table** **2**. The characteristic genes (or their encoded proteins) listed above can potentially serve as biomarkers to distinguish responders from non‐responders to immunotherapy.

**Table 2:** Featured genes in sub‐clusters of CD8^+^ T cells or CD4^+^ T cells.

Sub‐clusters in CD8^+^ or CD4^+^ T cells	Featured genes
Cluster 5 in CD8^+^ T cells	EIF2AK2, DDX60, RSAD2, LFIT3b, OAS2, GBP10, OASL1, ZBP1, Gbp6, LFIT1, LFIT3, RTP4, LSG15, LIGP1, OASL2, USP18, LSG20, CMPK2, and LFIH1
Cluster 8 in CD8^+^ T cells	S100A4, KLRG1, CX3CR1, S1PR5, KLRK1, itgax, fgl2, S100A8, S100A9, CCL3, and KLRC1
Cluster 9 in CD8^+^ T cells	Ki67 CDCA8, CDK1, UHRF1, KIF11, RRM1, KIF5, CCNB2, TACC3, UBE2C, SMC2, RRM2, TPX2, NCAPD2, CKS1b, CCNA2, CENPE, NCAPG, MCM5, SPC24, CENPF, NUSAP1, TYMS, NSD2, NCAPH, MAD2L1, ASF1b, CKAP2L、MCM7、NRM, H2AX、DUT、PRC1, CBX5, HMMR, KIF23, BUB1, KNL1, CDCA3, FBXO5, CLSPN, KIF22, DLGAP5, GMNN, NDC80, MIS18NP1, NUF2, RAD51, KIF4, AURKb, TK1, PRIM1、CIT, LOCKD, CHAF1A, TFDP1, CIP2a, CDC20, NCAPG2, ARHGAP11a, PIK1, HELLS, ASPM, CEP55, CKAP2, KIF20A, RFC5, TCF19, DIAPH3, BUB1b, ESCO2, GM10282, CIP2a, CENPH, KNSTRN, ATAD5, CDCA2, DTL, POLA1, NEK2, SGO1, CENPW, MXD3, SPAG5, PSAT1, CCDC34, ORC6, SGO2a, SHCBP1, KIF2C, CDK2, RFC4, DHFR, STIL, FOXM1, CCNB1, ESPL1, PRR11, TIPIN, MCM10, PPIL1, FIGNL1, E2F8, RAD51AP1, DEPDC1a, Hirip3, RFC3, CDCA7, KIF18b, POLE, CDCA5, CENPM, CDKN2c, RCC1, CHAF1b, H1F5, CENPL, CENPN, KPNA2, ANLN, H3C3, H2AC8, KIF14, KNTC1, HMGB3, TRIP13, MELK, BARD1, ARHGAP19, SPDL1, CDC6, BRCA1, CI1 SD1, PBDC1, BRCA2, PBK, CDKN3, GPSM2, CDC7, SKA1, RMI2, CCNF, CHEK1, CENPI, PIF1, CHTF18, PCLAF, Birc5, PLP2, EZH2, Lig1, CDK2a, CENPa, and RBBp8
Cluster 7 and 9 in CD8^+^ T cells	CTLA4 and Myb
Cluster 8 and 9 in CD8^+^ T cells	KLRA7, KLRA1, KLRE1, Myb, EOMES, PTMS, SLAMF7, ITGB1, CCL5, CTLA2a, CCR5, and CD44
Clusters 10 in CD4^+^ T cells	Ki67, TOP2a, PCLAF, BIRC5, CCNA2, UBE2C, RRM2, CENPF, CENPE, TPX2, NUSAP1, KIF11, and CCNB2
Clusters 0, 1, 2, 3, 4, and 6 in CD4^+^ T cells	IFNGr2, DUSP10, eif2ak3, PDE4d, and Nr4a3
Clusters 7 and 10 in CD4^+^ T cells	CXCR6
Clusters 8 and 10 in CD4^+^ T cells	CXCR5
Clusters 7, 9 and 10 in CD4^+^ T cells	CXCR3 and CCR2
Clusters 5, 8, 9 and 10 in CD4^+^ T cells	lkzf2, CTLA4, IL2rb, CD44, ICOS, IL2ra, and FOXP3
Clusters 7, 8, 9 and 10 in CD4^+^ T cells	lkzf2, CTLA4, IL2rb, CD44, ICOS, IL2ra, FOXP3, S100A4, S100A6, S100A11, S100A13, and CXCR3

The analysis of 11 sub‐clusters of CD4^+^ T cells in PBMC revealed that cells in sub‐clusters 0, 1, 3, and 4 increased in the healthy group and four different cured groups, with almost no sub‐cluster 10. Cells in sub‐clusters 2, 5, 6, 7, 9, and 10 were significantly increased in the un‐cured group (Figure 5h,i). In splenocytes, cells in sub‐clusters 0, 1, 2, 3, 4, and 6 increased in the healthy and cured groups, whereas cells in sub‐clusters 5, 7, 8, 9, and 10 significantly increased in the un‐cured group. However, no significant differences were observed in the DLN samples from un‐cured mice and cured mice.

Elevated genes in sub‐cluster 10 in CD4^+^ T cells included TOP2a, PCLAF, BIRC5, CCNA2, CENPF, TPX2, NUSAP1, KIF11, and CCNB2 (Figure 5j,k; Figures S18–S20, Supporting Information). The featured markers for sub‐clusters 5, 8, 9, and 10 included lkzf2, CTLA4, IL2rb, IL2ra, and FOXP3. The featured markers for sub‐clusters 7, 9, and 10 were CXCR3 and CCR2. The featured markers for sub‐clusters 7, 8, 9, and 10 included IL2ra, lkzf2, CTLA4, FOXP3, S100A4, S100A6, S100A11, S100A13, and CXCR3. IL2Ra (CD25), IKZF2, CTLA4 and FOXP3 have been connected to exhausted T cells^[^ ^59^, ^60^, ^61^ ^]^ and regulatory T cells (T_reg_).^[^ ^62^, ^63^ ^]^ T_regs_ that highly express IKZF2 have higher expression of Foxp3 and decrease the ability to produce IFN‐γ and TNF‐α.^[^ ^64^ ^]^ Increased genes in subclusters 7 and 10 was CXCR6. The feature marker for sub‐clusters 8 and 10 was CXCR5. The featured markers for sub‐clusters 0, 1, 2, 3, 4, and 6 included IFNGr2, DUSP10, eif2ak3, PDE4d, and Nr4a3. IFNGr2 is a marker of cytotoxic T cells indicated that these sub‐clusters possess cytotoxic functions. The sub‐clusters 5, 7, 8, 9, and 10 of CD4^+^ T cells are T_reg_ and exhausted T cells. Sub‐clusters 0, 1, 2, 3, 4, and 6 of CD4^+^ T cells are cytotoxic T cells. These indicated that CD4^+^ T cells in the healthy and cured groups have more cytotoxic T cells, while CD4^+^ T cells in un‐cured group have more regulatory T cells and exhausted T cells. The characteristic genes or their encoded proteins listed above can potentially be used as biomarkers to distinguish immunotherapy responders from non‐responders.

### 2.16 Identified Biomarkers Can Distinguish Cured Mice and Un‐Cured Mice

To verify the feasibility of applying featured markers in specific sub‐clusters as biomarkers to predict or distinguish responders and non‐responders to immunotherapy (αPD‐1 antibody + cisplatin), the levels of some featured markers in the splenocytes of cured and uncured mice (LLC) after immunotherapy were detected by using flow cytometry. Before labeling the splenocytes with featured markers and performing flow cytometry, the splenocytes were co‐incubated with NPs loaded with LLC tumor tissue lysates. The selected markers were S100A4, KLRG1, IL2Ra (CD25), CX3CR1, IKZF2, and S1PR5. As described above, S100A4,^[^ ^53^, ^54^, ^55^ ^]^ KLRG1,^[^ ^49^, ^50^, ^51^, ^52^ ^]^ IL2Ra (CD25),^[^ ^59^, ^60^, ^61^ ^]^ CX3CR1,^[^ ^56^, ^57^, ^65^ ^]^ IKZF2,^[^ ^62^, ^63^, ^64^ ^]^ and S1PR5^[^ ^58^ ^]^ are all markers that related with inhibiting or damaging the function of cytotoxic T cells.

**Figure 6:**
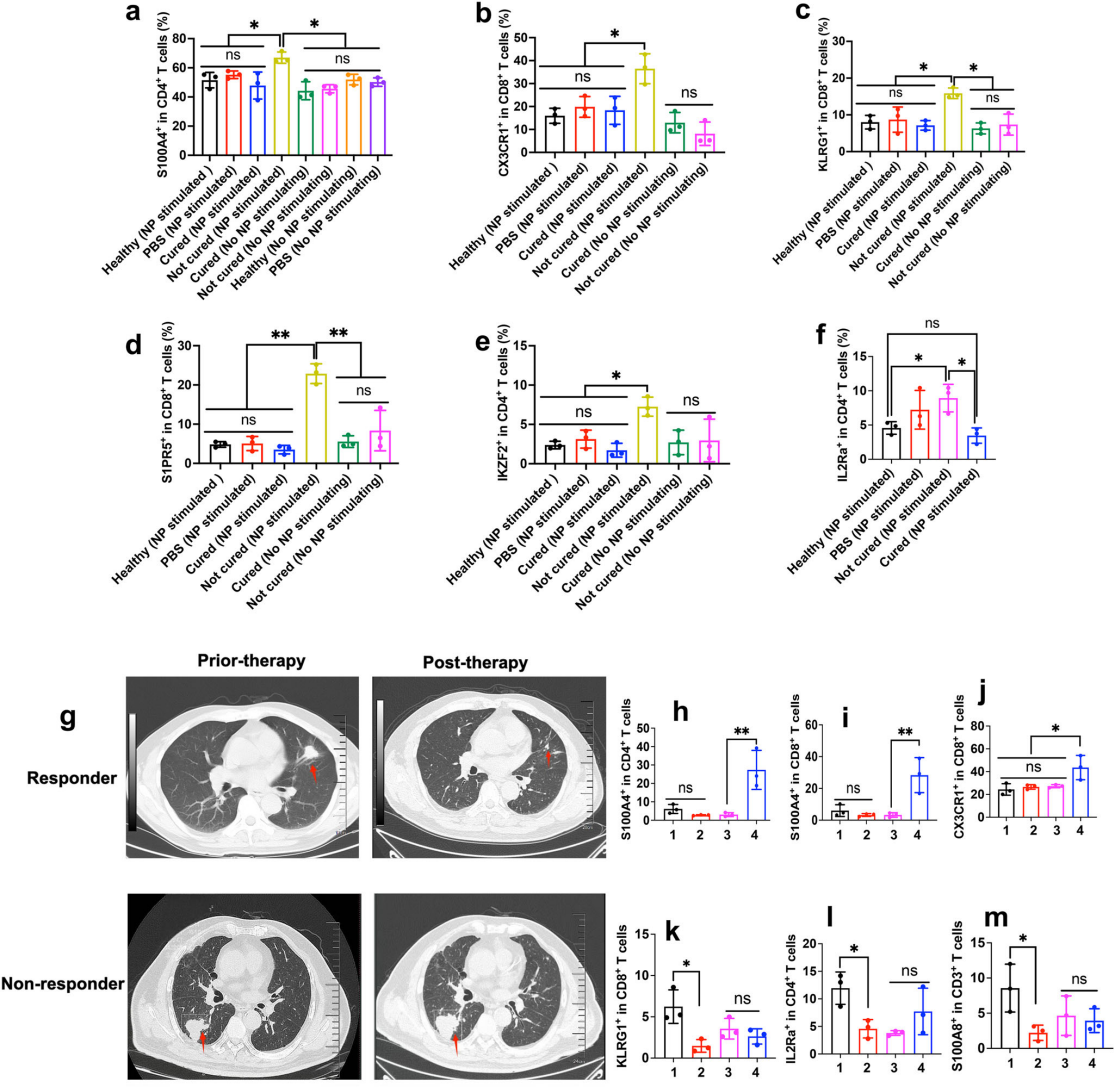
Identified markers expressed differently in responders and non‐responders to immunotherapy in the mouse model and patients with lung cancer. a–f) analysis of various featured markers of T cells using flow cytometry in cured and uncured tumor‐bearing mice (LLC lung cancer, n ≥ 3). g) Prior‐ and post‐therapy CT images (patients with non‐small cell lung cancer) of responder and non‐responder to αPD‐1 antibody therapy (a red arrow labels the site of tumor tissue). h–m) analysis of various featured markers of T cells using flow cytometry in responders and non‐responders to αPD‐1 antibody in patients with lung cancer (*n* = 3). **1**, Responder, pre‐therapy; **2**, Responder, ongoing‐therapy (42 days); **3**, non‐responder, pre‐therapy; **4**, non‐responder, ongoing‐therapy (42 days). Each data point represents one mouse or one patient with cancer. Data are presented as Mean ± SD. *p*‐values <0.05 were considered significant: ^*^ *p* < 0.05, ^**^ *p*<0.01.

According to the results showed in **Figure** **6**a–f, Figures S21 and S22 (Supporting Information), S100A4^+^ in CD4^+^ T cells, CX3CR1^+^ in CD8^+^ T cells, KLRG1^+^ in CD8^+^ T cells, SIPR5^+^ in CD8^+^ T cells, IL2Ra^+^ in CD4^+^ T cells, and IKZF2^+^ in CD4^+^ T cells were significantly increased in post‐therapy samples from uncured mice compared to prior‐therapy, whereas samples from cured mice did not. These results were consistent with those obtained from single‐cell sequencing, illustrating the feasibility of using these biomarkers to predict the therapeutic efficacy of cancer immunotherapy.

### 2.17 Biomarkers Can Predict Immunotherapy Efficacy in Patients with Lung Cancer

To further verify the feasibility of utilizing selected featured markers as biomarkers to predict responders and non‐responders to immunotherapy, the number of several featured markers in the PBMC of patients with lung cancer and prior and ongoing immunotherapy was detected using flow cytometry.

Before detecting the selected markers, immune cells were co‐incubated with NPs loaded with whole tumor antigens and labeled with various flow cytometry antibodies. To explore a universal whole‐tumor antigen source, the feasibility of utilizing multiple cancer cell lines to replace autologous tumor tissues was tested and verified. As shown in Figure S23 (Supporting Information), lysates of mixed multiple cancer cell lines could achieve the same efficacy as lysates of autologous non‐small lung cancer tumor tissues in preparing NPs for activating T cells, whereas lysates of allogeneic non‐small lung cancer tumor tissues could not. These results proved that NPs loading lysates of multiple mixed cancer cell lines could be applied as universal NPs to activate T cells for the following detection.

These markers are positively correlated with the efficacy of immunotherapy in mouse models. These markers were further investigated in patients with lung cancer to confirm their correlation with immunotherapy efficacy, and NPs loading lysates of mixed multiple cancer cell lines were utilized as activating NPs. As shown in Figure 6g–m, Figures S24 and S25 (Supporting Information), S100A4^+^ in CD4^+^ T cells, S100A4^+^ in CD8^+^ T cells, and CX3CR1^+^ in CD8^+^ T cells significantly increased in ongoing therapy blood samples from non‐responders compared to prior‐therapy; KLRG1^+^ in CD8^+^ T cells, IL2Ra^+^ in CD4^+^ T cells, and S100A8^+^ in CD3^+^ T cells significantly decreased in ongoing therapy blood samples from responders compared to prior therapy. These results of featured biomarkers are consistent with the PET‐CT images of patients with non‐small lung cancer taken before immunotherapy and 6 months post‐immunotherapy (Figure 6g, tumor size decreased in responders after treatment but did not decrease in non‐responders). These data in patients with lung cancer are consistent with the results obtained from single‐cell sequencing and mouse models, demonstrating that utilizing the biomarkers identified in this study to predict the therapeutic efficacy of cancer immunotherapy is a feasible strategy.

## 3 Discussion

Cancer vaccines are critical for translating cancer from a lethal to a chronic, non‐lethal disease. Currently, cancer vaccines have limited therapeutic efficacy when used alone. To maximize the efficacy of cancer vaccines, several parameters can be altered, including adjuvants, formulations, and tumor antigens.^[^ ^1^, ^2^, ^3^ ^]^ In this study, several conditions related to these three parameters were systematically explored to identify optimal cancer vaccines loaded with whole tumor antigens.^[^ ^21^ ^]^ To the best of our knowledge, for the first time, many crucial conditions for maximizing vaccine efficacy were explored in this study, including single adjuvants, combinations of adjuvants, antigen loading sites, sizes of cancer vaccines, methods of collecting, fixing, and lysing tumor tissues, antigen composition, antigen purity, methods of purifying antigens, and methods of oxidizing lysates. The therapeutic efficacy of cancer vaccines can be maximized by exploring the optimal parameters for these conditions.

Most cells in tumor tissues are tumor cells. Besides, some other cells, such as fibroblasts and immune cells, also exist in tumor tissues. Our bodies have central immune tolerance and peripheral immune tolerance to the components of normal cells, such as fibroblasts. Therefore, only tumor antigens in tumor tissues can efficiently activate antigen‐specific T cells. In our previous stusies, nanovaccines loaded with whole tumor cell lysates and nanovaccines loaded with whole tumor tissue lysates showed similar therapeutic efficacy in B16F10 mouse model^3^. In addition, in clinic, getting tumor tissues is more convenient than getting pure tumor cells from patients with cancer. Therefore, we used the lysates of tumor tissues to prepare cancer nanovaccines/micronvaccines in this study.

Ideal adjuvants should efficiently activate signal 2 and signal 3 in APCs for T cell activation. In addition, to keep minimum potential side effects, the receptors or targets of adjuvants should only or mainly be located in APCs. Based on these principles, several different adjuvants and their combinations have been systematically investigated in terms of therapeutic efficacy to identify optimized adjuvants. The results revealed that poly(I:C) combined with specific types of CpG ODN (type B or C) performed best at amplifying the tumor‐specific immune response.

Nanovaccines and micronvaccines with different sizes were compared in treating cancer and the results illustrated that <400 nm could be the optimum size of cancer nanovaccines and 2.5µm could be the optimum size of cancer micronvaccines. The injected particles can enter into draining lymph node (DLNs) in two different ways: one way is entering into DLN by themselves; the second way is by the homing effect of APCs that uptake particles at the injection site. Smaller nanoparticles mainly enter into DLNs by themselves. However, larger particles especially micronparticles mainly rely on recruiting APCs to the injection site and being uptake by APCs at the injection site. Some studies reported that nanovaccines with size < 100 nm showed some advantages.^[^ ^66^, ^67^, ^68^, ^69^, ^70^ ^]^ Depending on the surface properties and the material properties, the optimal size for different cancer nanovaccines could be variant.^[^ ^28^, ^66^, ^67^, ^68^, ^69^, ^70^, ^71^ ^]^

Tumor cells and tumor antigens are highly heterogeneous, and thus, more diverse tumor antigen‐specific T cells are needed to recognize various tumor antigens and kill tumor cells containing such tumor antigens. Therefore, cancer vaccines containing multitudinous tumor antigens have advantages. Tumor cell/tissue lysates contain various tumor antigens, including both TSAs and TAAs. Therefore, tumor cell/tissue lysates are good tumor antigens to prepare cancer vaccines. The processing of these whole tumor antigens in tumor cell/tissue lysates can affect the therapeutic efficacy of cancer vaccines. Our studies on the processing of whole tumor antigens have demonstrated that freezing, fixation, lysis, oxidation, heating, and purification can significantly affect the immunogenicity of tumor antigens. Processing tumor antigens using appropriate methods (such as oxidation and purification) can dramatically improve the efficacy of cancer vaccines.

Optimized cancer vaccines can cure most tumor‐bearing bodies in melanoma, lung cancer, subcutaneous pancreatic, orthotopic pancreatic, and melanoma lung metastasis models. This represents a significant improvement in the therapeutic efficacy of cancer nanovaccines. In addition, no potential toxicity was observed with these cancer vaccines.

Understanding why specific individual responses to immunotherapy can help us optimize therapeutic strategies. Therefore, 21 samples from the blood, splenocytes, and DLN of immunotherapy‐cured and un‐cured mice were systematically investigated by using single‐cell sequencing. It was discovered that cured mice (responders) have much higher diversities in CD8^+^ TCR, CD4^+^ TCR, and BCR in the blood and spleen than those of uncured mice (non‐responders). These results confirmed the necessity of activating more diverse tumor antigen‐specific T cells to get better therapeutic efficacy because more diverse tumor antigen‐specific T cells can recognize more tumor cells. Therefore, cancer vaccines that can induce more diverse antigen‐specific T cells could have better therapeutic efficacy.

Furthermore, immune cell profiles in the blood and splenocytes in the four different cured groups were similar, and they were significantly different from those in the un‐cured group. In addition, immune cell profiles in the blood and splenocytes in the different cured groups (freshly cured, cured for a period of time, or cured for a long time) were similar to those in the healthy group. In contrast, the immune cell profiles in the blood or splenocytes in the un‐cured group were different from those in the cured and healthy groups. This phenomenon indicates that immune cell profiles in the peripheral blood, particularly T cell profiles, can be used to characterize therapeutic efficacy.

By analyzing the landscape of immune cell clusters and sub‐clusters in CD8^+^ T cells or sub‐clusters in CD4^+^ T cells, clusters that specifically existed in responders and non‐responders were discovered. Such clusters can be used to differentiate between individuals responsive and non‐responsive to immunotherapy. Gene and function analysis revealed that responders of immunotherapy have more T cells in clusters possessing cytotoxic functions and non‐responders of immunotherapy have more clusters with T_regs_ or exhausted T cells. These clusters were identified with featured markers that included KLRG1, SIPR5, S100A4, CXCR3, IL2Ra, IKZF2, EMB, NR4A3, RAMP3, CX3CR1, S100A9, EIF2AK3, and DUSP10 etc. Thus, our study demonstrated that these markers have the potential to be used as biomarkers for distinguishing non‐responders to immunotherapy. Moreover, some of these markers were selected to verify the feasibility of their use as biomarkers to identify non‐responders to immunotherapy, both in mouse cancer models and in patients with lung cancer. The results demonstrated that the selected markers (including KLRG1 and S100A4) were differentially expressed in T cells from responders and non‐responders, indicating the practicability of utilizing featured markers to predict therapeutic efficacy. We propose and prove why our biomarkers, such as S100A4^+^ in CD4^+^ T cells, S100A4^+^ in CD8^+^ T cells, KLRG1^+^ in CD8^+^ T cells, S1PR5^+^ in CD8^+^ T cells, IL2Ra^+^ in CD4^+^ T cells, S100A8^+^ in CD3^+^ T cells, and IKZF2^+^ in CD4^+^ T cells, can be utilized to predict immunotherapy efficacy upon stimulation with NPs loaded with whole tumor antigens.

Furthermore, the results demonstrated that the diversity of CD8^+^ TCR, CD4^+^ TCR, and BCR in PBMC and splenocytes of different cured groups was much higher than that in the un‐cured group. These data illustrated that more diverse tumor antigen‐specific T cells are induced in the responders of immunotherapy.

This study has some potential limitations. The detection of biomarkers was conducted in a small number of patients with lung cancer, and more data from patients with different cancer types could further verify the results. The other biomarkers listed in Table 2 should be further investigated in future studies.

In summary, this study presents a highly efficient cancer nanovaccine for loading whole tumor antigens by optimizing adjuvants, vaccine formulations, and tumor antigens. These optimized cancer vaccines cured all or most tumor‐bearing bodies in mouse models of melanoma, lung cancer, pancreatic cancer, and melanoma lung metastasis. In addition, the landscape and differences in immune cells in the blood, splenocytes, and DLN of responders and non‐responders were determined by single‐cell sequencing to explore the featured biomarkers of non‐responders to immunotherapy. Importantly, several biomarkers (such as CX3CR1 and S100A4) were identified and verified to distinguish between responders and non‐responders to immunotherapy in both cancer mouse models and patients with cancer. In future studies, biomarkers will be investigated for different types of cancers to determine their versatility. In addition, clinical trials to verify the efficacy of optimal cancer vaccines for patients with cancer who will undergo tumor resection surgery could be conducted.

## 4 Experimental Section

### Reagents

PLGA (503H, RESOMER RG, 503530) was purchased from EVONIK; PVA (360627, MW: 9000–10000 Da); Ficoll reagent, PVA and Urea were purchased from Sigma–Aldrich; Collagenase /hyaluronidase and fetal bovine serum (FBS) were purchased from HyClone; Dulbecco's modified eagle medium (DMEM)/High Glucose, RPMI 1640, Penicillin–Streptomycin Solution (P/S), Trypsin Cell Digestion Solution, and Red Blood Cell Lysis Buffer were purchased from Pricella; AIM V serum‐free medium was purchased from Thermo Fisher; DC2.4 cell line, B16F10 mouse melanoma cell line, LLC mouse lung cancer cell lines, KPC moue pancreatic cancer cell line, A549 human lung cancer cell line, H1299 human lung cancer cell line, H1650 human lung cancer cell line, PC9 human lung cancer cell line, H226 human lung cancer cell line, H520 human lung cancer cell line, and SK‐MES‐1 human lung cancer cell line were purchased from the Shanghai Cell Bank, Chinese Academy of Sciences; IL‐4, IL‐2, and GM‐CSF were all purchased from Perprotech; 4% Paraformaldehyde, CCK8 and anti‐fluorescence quenching agent were purchased from Beijing Solaibao Technology Co., Ltd; All antibodies for flow cytometry were purchased from Biolegend or BD.

### Ethics of Animal Studies

All animal procedures were approved (Approved ethics number 202310A0713) and monitored by the Animal Care and Use Committee of the Soochow University. The mice were housed in a specific pathogen‐free (SPF) animal room at the School of Pharmacy, Soochow University, with a constant temperature of 22 ± 1 °C, relative humidity of 50 ± 10%, 12‐h artificial light cycle, and automatic ventilation, adhering to the guidelines outlined in the Guide for the Care and Use of Laboratory Animals.

### Ethics of Studies Applied Human Blood Samples

This study was a prospective, non‐interventional investigation registered prospectively in February 2022 on Clinical Trials (https://classic.clinicaltrials.gov/) with registration number NCT05789498. This study was approved by the Ethics Committee of the First Affiliated Hospital of Soochow University (approval number 2022181). All experiments adhered to the principles of the Declaration of Helsinki and all patients provided informed consent.

### Preparation of Whole Tumor Tissue Lysates

Two different methods were utilized to prepare whole tumor tissue lysates: 1) The first method involves adding ultrapure water to tumor tissues and lysing the tumor tissue by repeating freezing–thawing of tumor tissue in pure water five times (with sonication for thawing frozen tumor tissue). Whole tumor tissue lysates were centrifuged at 12000 RPM for 20 min. The supernatant was defined as water‐soluble components, the precipitate was solubilized with 8 m urea (or 6 m hydrochloride, 5% sodium deoxycholate, or 1 m metformin hydrochloride), and the solubilized precipitate was defined as solubilized water‐insoluble components. 2) The second method was using 8 m urea (or 6 m hydrochloride) to lyse tumor tissues (frozen or fixed), and then using 8 m urea (or 6 m hydrochloride) was used to solubilize whole tumor tissue lysates.

### Fixation of Whole Tumor Tissue

Different fixation solutions were used to fix the tumor tissues overnight. The cells were fixed with 75% ethanol, formalin, paraformaldehyde, glacial acetic acid, 75% ethanol + 3%H_2_O_2_, or 75% ethanol + 200nm HClO.

### Purification of Whole Tumor Tissue Lysates

Salting‐out, ethanol precipitation, and heating were performed to purify the tumor tissue lysates. During the salting‐out process, 10% ammonium sulfate was added to the tumor tissue lysates and precipitated overnight, followed by centrifuging at 12 000 RPM for 30 min to collect the precipitated protein peptides and resolubilize them with 8 m urea or 6 m hydrochloride. During the ethanol precipitation process, 75% ethanol was added to the tumor tissue lysates and precipitated overnight, followed by centrifuging at 12 000 RPM for 30 min to collect the precipitated protein peptides and resolubilize them with 8 m urea or 6 m hydrochloride. In the heating process, tumor tissue was heated at 95 °C for 30 min, followed by centrifuging at 12 000 RPM for 30 min to collect the precipitated proteins–peptides and re‐solubilizing the precipitated protein/peptides with 8 m urea or 6 m hydrochloride.

### Oxidation of Whole Tumor Tissue Lysates

Two oxidation solutions, 3%H_2_O_2_ and 200 nm HClO, were used in the study. The oxidation process involved incubating the tumor tissue with an oxidation solution for more than 2 h.

### Preparation of Nanoparticles Loaded with Water‐Soluble Components in Tumor Tissue Lysates

The methods used to prepare whole tumor tissue lysates (including both water‐soluble components and 8 m urea solubilized water‐insoluble components) were as previously reported. The double emulsion method was used to prepare nanoparticles. The procedure was as follows: First, 300 µL of the water‐soluble fraction dissolved in endotoxin‐free water (80 mg mL^−1^) was added to 1 mL of dichloromethane solution (containing 100 mg mL^−1^ PLGA) and sonicated for 1 min. The amount of total TLR adjuvant applied was 6 mg (If applied Poly I: C+C pG, 4 mg poly (I:C), and 2 mg). Then, 2.5 mL of PVA solution (20 mg mL was added, followed by 45 s of sonication. The mixture was then dripped into a 50 mL solution containing 5 mg mL^−1^ of PVA and stirred at room temperature for 4 h to facilitate nanoparticle solidification. Finally, the supernatant was removed by centrifugation at 13680 × g and the precipitate was resuspended in 10 mL of 4% trehalose solution. After freeze‐drying for 48 h, the nanoparticles were stored at −20 °C for future use. Throughout the preparation process, strict endotoxin‐free procedures were performed to ensure the quality of the nanoparticle vaccine. These nanoparticles were characterized according to the methods described in the previous publications.

### Preparation of Nanoparticles Loaded with Water‐Insoluble Components in Tumor Tissue Lysates

The precipitate of the centrifuged tumor tissue lysates was solubilized in 8 m urea, wherein 80 mg mL^−1^ of water‐insoluble components was dissolved in 300 µL of 8 m urea (or 6 m hydrochloride, or 5% sodium deoxycholate, or 1 m metformin hydrochloride), followed by the addition of 1 mL of 100 mg mL^−1^ PLGA in dichloromethane solution and sonication for 1 min. The subsequent steps were the same as those used for the preparation of nanoparticles loaded with water‐soluble cellular components. These nanoparticles were characterized according to the methods described in the previous publications.

### Preparation of Nanoparticles Loaded with Tumor Tissue Lysates Lysed and Solubilized Directly with Solubilizers

Tumor tissues were frozen (or fixed with 75% ethanol, formalin, paraformaldehyde, glacial acetic acid, 75% ethanol + 3%H_2_O_2_, or 75% ethanol + 200nm HClO), and then the treated tumor tissue was lysed with 8 m urea or 6 m hydrochloride (or centrifuge at 5000 RPM 10 min to remove the fixation solution and solubilized the precipitate with 8 m urea or 6 m hydrochloride). The solubilized tumor tissue lysates were then loaded into nanovaccines or microvaccines. The methods used to prepare the nanovaccines and microvaccines were the same as those previously reported using the double‐emulsion method. The procedure was as follows: First, 300 µL of solubilized tumor tissue lysates (80 mg mL^−1^) was added to 1 mL of dichloromethane solution (containing 100 mg mL^−1^ PLGA) and sonicated for 1 min. Then, 2.5 mL of PVA solution at a concentration of 20 mg mL^−1^ was added, followed by 45 s of sonication. The mixture was then dripped into a 50 mL solution containing 5 mg mL^−1^ of PVA and stirred at room temperature for 4 h to facilitate nanoparticle solidification. Finally, the supernatant was removed by centrifugation at 13680 × g and the precipitate was resuspended in 10 mL of 4% trehalose solution. After freeze‐drying for 48 h, the nanoparticles were stored at −20 °C for future use. Throughout the preparation process, strict endotoxin‐free procedures were performed to ensure the quality of the nanoparticle vaccine. These nanoparticles were characterized according to the methods described in the previous publications.

### Preparation of Nanoparticles Loaded with Multiple Neoantigen Peptides

The double‐emulsion method was used to prepare nanoparticles loaded with multiple neoantigen peptides (B16F10‐M36, B16F10‐M05, B16F10‐M27, B16F10‐M20, B16F10‐M24, gp100:44‐59, and TRP2:180‐188). The peptides were solubilized in 8 m urea at a concentration of 2 mg mL^−1^. The total peptide concentration was 14 mg mL^−1^. Peptide solution of 300 µL was added to 1 mL of dichloromethane solution (containing 100 mg mL^−1^ PLGA) and sonicated for 1 min. Then, 2.5 mL of PVA solution at a concentration of 20 mg mL^−1^ was added, followed by 45 s of sonication. The mixture was then dripped into a 50 mL solution containing 5 mg mL^−1^ of PVA and stirred at room temperature for 4 h to facilitate nanoparticle solidification. Finally, the supernatant was removed by centrifugation at 13680 × g and the precipitate was resuspended in 10 mL of 4% trehalose solution. After freeze‐drying for 48 h, the nanoparticles were stored at −20 °C for future use. Throughout the preparation process, strict endotoxin‐free procedures were performed to ensure the quality of the nanoparticle vaccine.

### Preparation of Nanoparticles Loading Lysates of Tumor Tissues from Patients with Lung Cancer

Autologous or allogeneic tumor tissues from patients with non‐small lung cancer were lysed and solubilized in 8 m urea. Additionally, seven human lung cancer cell lines (A549, H1299, H1650, PC9, H226, H520, and SK‐MES‐1) were lysed and solubilized in 8 m urea, followed by mixing at a ratio of 1:1. These NPs all have the size of ≈250 nm, and have the zeta potential ≈−25.0 MV. The loading capacity of proteins/peptides was ≈100 µg/1 mg PLGA.

### Characterization of Nanoparticles Loading Whole Tumor Tissue Lysates

The size and zeta potential of the NPs were determined by dynamic light scattering (DLS; Zetasizer Nano‐ZS, Malvern Instruments, Worcestershire, UK). The amount of proteins/peptides loaded within the NPs was determined using a microBCA assay or a Nanodrop.

The morphology of the NPs was investigated using transmission electron microscopy (TEM; HT7700). In total, 20 µL of 0.1 mg mL^−1^ PLGA particles was dropped onto a lacey copper grid coated with a continuous carbon film. The samples were then dried and observed by TEM.

### Cellular Uptake and Endosomal Escape of Particles

DC2.4 cells were seeded in six‐well plates (5.0 × 10^5^ cells per well) and incubated overnight in DMEM containing 10% fetal bovine serum. NPs or MPs (rhodamine labeled nanoparticles, 0.5 mg mL^−1^) were incubated with cells for 6, 12, and 24 h. Then, the cells were washed thrice using PBS and incubated with 0.5 mL of trypsin for 3 min, followed by cell counting. The cells were collected using a similar procedure and measured using a FACSCaliber system (BD Biosciences). In the endosome escapes studies, DC2.4 cells (2.0 × 10^5^ cells per well) were treated with NPs or MPs (Rhodamine labeled Nanoparticles) for 24 h at 37 °C. After 30 min, cells were stained with DAPI and LysoTracker Green DND‐26 (5.0 ug mL^−1^, 1.0 mL) for 20 min. Finally, the cells were washed three times with PBS and observed using CLSM (Zeiss LSM710).

### Therapeutic Efficacy Studies Performed on Melanoma and Lung Cancer Mouse Model

All C57BL/6J mice used to establish the tumor mouse models were obtained from Shanghai Jihui Experimental Animal Co., Ltd., aged 6–8 weeks. A subcutaneous tumor model was established by injecting 1.5 × 10^5^ B16F10 melanoma cancer cells or 2.0 × 10^6^ LLC lung cancer into the right back of mice. Tumor cell inoculation was set at day 0, and the tumor volume and body weight of the mice were measured every 3 days from day 0. In the treated groups, 200 µL of PBS or NPs/MPs (2 mg/mice/time) were injected subcutaneously into the back of mice on days 3, 6, 9, 14, 19, and 26. The tumor volume was calculated using the formula: V (tumor volume) = 0.52 × a × b^2^, where a and b represent the long and short diameters of the tumor measured using a Vernier caliper, respectively. If the weight of the mouse decreased by more than 10% from the initial value, or if the tumor volume exceeded 2000 mm^3^, the body weight and tumor volume were monitored and the mice were euthanized.

### Therapeutic Efficacy of Nanovaccines on Subcutaneous KPC Pancreatic Cancer

In a subcutaneous pancreatic cancer mouse model, 2.0 × 10^6^ KPC‐Fluc cells were subcutaneously injected into the right back of C57BL/6J mice on day 0. In the treated groups, 200 µL of PBS or NPs/MPs (2 mg/mice/time) were injected subcutaneously into the back of mice on days 3, 6, 9, 14, 19, and 26. Tumor volume was recorded every 3 days, beginning on day 3. In the chemotherapy control group (Gemcitabine/ABR), Gemcitabine (GEM, 35.0 mg kg^−1^) and ABRAXANE (ABR, Paclitaxel‐albumin, 8.0 mg kg^−1^) were intravenously injected on day 3 and 11. The tumor volume and body weight of the mice were recorded every 3 days beginning on day 0.

### Therapeutic Efficacy of Nanovaccine on Melanoma Metastasis in Lung

Lung metastasis from a melanoma mouse model was established by injecting 3.0 × 10^4^ B16F10 cells intravenously into each C57BL/6L mouse on day 0. On days 3, 6, 9, 14, and 19, the mice were treated with 2 mg of nanovaccines (NP 144). On day 24, the mice were sacrificed, and the lungs were isolated to record the tumor numbers.

### Therapeutic Efficacy of Nanovaccines on Orthotopic KPC Pancreatic Cancer

Approximately 3 × 10^5^ KPC‐Luc cells were injected into the tail of the pancreas of female C57BL/6J mice (6‐8 weeks) to establish an orthotopic pancreatic cancer model. Mice bearing orthotopic tumors were randomly divided into different groups (*n* = 8) and received various treatments, including PBS, NP230, and NP231. PBS or NPs were administered subcutaneously on Days 3, 6, 9, 14, 19, and 26. Bioluminescence images of mice were acquired every 5 days after the injection of NPs using an IVIS Spectrum Lumina III (Perkin Elmer). Survival was monitored for 60 days after tumor inoculation.

### Evaluating Potential Toxicities by Measuring Serum Cytokines after Treatment with NPs

C57BL/6J mice (6–8 weeks) were divided into different groups and received six times subcutaneous treatment with PBS or NPs six times. The dose was 2 mg/mice/time NPs. The mice were sacrificed on day 27 and blood samples were collected. The serum levels of IL‐2, IL‐6, TNF‐α, and IFN‐γ were measured using corresponding ELISA kit.

### Evaluating Potential Toxicities by Testing Blood Biochemistry Analysis after Treatment

C57BL/6J mice (6–8 weeks) were divided into different groups and received six times subcutaneous treatment with PBS or NPs six times. The dose used was 2 mg/mice/time NPs. Mice were sacrificed on day 27, and blood was collected for biochemical examination.

### Evaluating Potential Toxicities in Major Organs using H&E Staining

The heart, liver, spleen, lungs, and kidneys of the mice were isolated, soaked, and fixed in paraformaldehyde solution. The organs were then stained with hematoxylin and eosin (H&E), followed by observation of tissue morphology using a microscope.

### Treatment of Tumor‐Bearing Mice with **α**PD‐1 Antibody and Cisplatin

To establish a lung cancer mouse model, 2.0 × 10^6^ LLC lung cancer cells were subcutaneously injected into the right back of C57BL/6J mice on day 0. αPD‐1 antibody (10 mg kg^−1^) was intraperitoneally administered, and cisplatin (1 mg kg^−1^) was administered intraperitoneally on days 6, 9, 12, 15, and 18. The mice were sacrificed on day 30 to isolate splenocytes and measure the marker features of T cells.

### Preparation of Single‐Cell Suspension of Splenocytes or Lymph Node Cells

Four C57BL/6 mice from each group were euthanized, and tissues (the spleen or DLN) were collected. Briefly, samples were washed with PBS, minced into small pieces (≈1mm^3^), and sieved through a 70 µm cell strainer. Cells from four mice in the same group were mixed for single‐cell sequencing. Dissociated single cells were stained for viability assessment using calcein‐AM (Thermo Fisher Scientific) and Draq7 (BD Biosciences).

There were total seven groups: 1) healthy mice, 2) PBS group, 1.5 × 10^5^ B16F10 subcutaneously injected on day 0, treated with 200 L PBS on days 3, 6, 9, 14 and 19, with mice sacrificed on day 21; 3) uncured group, 1.5 × 10^5^ B16F10 subcutaneously injected on day 0, treated with 200 L PBS on days 3, 6, 9, 14, 19 and 25, with mice sacrificed on day 30; 4) freshly cured group, 1.5 × 10^5^ B16F10 subcutaneously injected on day 0, treated with 2 mg NP 31 (1 mg NPs loading water‐soluble components+1 mg NPs loading 8 m urea solubilized water‐insoluble components) on days 3, 6, 9, 14, 19 and 25, mice were sacrificed on day 30; 5) cured after medium period group, 1.5 × 10^5^ B16F10 subcutaneously injected on day 0, treated with 2 mg NP 31 (1 mg NPs loading water‐soluble components+1 mg NPs loading 8 m urea solubilized water‐insoluble components) on days 3, 6, 9, 14, 19, 25 and 117, with mice sacrificed on day 120; 6) cured after long period group, 1.5 × 10^5^ B16F10 subcutaneously injected on day 0, treated with 2 mg NP 31 (1 mg NPs loading water‐soluble components+1 mg NPs loading 8 m urea solubilized water‐insoluble components) on days 3, 6, 9, 14, 19, 25 and 237, with mice sacrificed on day 240; 7) cured after long period and rechallenged with cancer cells group, 1.5 × 10^5^ B16F10 subcutaneously injected on day 0, treated with 2 mg NP 31 (1 mg NPs loading water‐soluble components+1 mg NPs loading 8 m urea solubilized water‐insoluble components) on days 3, 6, 9, 14, 19, and 25, with mice rechallenged with 1.5 × 10^5^ B16F10 on day 237 and sacrificed on day 240.

### Peripheral Blood Mononuclear Cells (PBMC) Isolation

Four C57BL/6 mice from each group were sacrificed and fresh blood samples were collected in EDTA anticoagulant tubes. Blood samples were immediately subjected to PBMCs isolation using standard density gradient centrifugation. After washing with PBS containing 0.04% BSA, the cell pellets were re‐suspended in PBS containing 0.04% BSA and re‐filtered through a 35 µm cell strainer. PBMC isolated from four mice in each group were mixed for single‐cell sequencing. Single PBMCs were stained for viability assessment using Calcein‐AM (Thermo Fisher Scientific) and Draq7 (BD Biosciences).

There were total seven groups: 1) healthy mice, 2) PBS group, 1.5 × 10^5^ B16F10 subcutaneously injected on day 0, treated with 200 L PBS on days 3, 6, 9, 14 and 19, with mice sacrificed on day 21; 3) uncured group, 1.5 × 10^5^ B16F10 subcutaneously injected on day 0, treated with 200 L PBS on days 3, 6, 9, 14, 19 and 25, with mice sacrificed on day 30; 4) freshly cured group, 1.5 × 10^5^ B16F10 subcutaneously injected on day 0, treated with 2 mg NP 31 (1 mg NPs loading water‐soluble components+1 mg NPs loading 8 m urea solubilized water‐insoluble components) on days 3, 6, 9, 14, 19 and 25, with mice sacrificed on day 30; 5) cured after medium period group, 1.5 × 10^5^ B16F10 subcutaneously injected on day 0, treated with 2 mg NP 31 (1 mg NPs loading water‐soluble components+1 mg NPs loading 8 m urea solubilized water‐insoluble components) on days 3, 6, 9, 14, 19, 25, and 117, with mice sacrificed on day 120; 6) cured after long period group, 1.5 × 10^5^ B16F10 subcutaneously injected on day 0, treated with 2 mg NP 31 (1 mg NPs loading water‐soluble components+1 mg NPs loading 8 m urea solubilized water‐insoluble components) on days 3, 6, 9, 14, 19, 25, and 237, with mice sacrificed on day 240; 7) cured after long period and rechallenged with cancer cells group, 1.5 × 10^5^ B16F10 subcutaneously injected on day 0, treated with 2 mg NP 31 (1 mg NPs loading water‐soluble components+1 mg NPs loading 8 m urea solubilized water‐insoluble components) on days 3, 6, 9, 14, 19, and 25, with mice were rechallenged with 1.5 × 10^5^ B16F10 on day 237 and sacrificed on day 240.

### Single‐Cell RNA Sequencing

A total of 21 samples from the above‐described 7 groups were analyzed. Each sample was a mixture of samples from four mice of the same group. All single‐cell sequencing data were uploaded to the NCBI public website (GEO ticket# 24748020; GEO submission: GSE273375).

The BD Rhapsody system was used to capture transcriptomic information of single cells. Single‐cell capture was achieved by the random distribution of a single‐cell suspension across > 200000 microwells using a limited dilution approach. Beads with oligonucleotide barcodes were added to saturation such that each bead was paired with a cell in a microwell. The cells were then lysed in a microwell to hybridize the mRNA molecules to the barcoded capture oligos on the beads. The beads were collected in a single tube for cDNA synthesis and library construction. The gene expression and V (D) J libraries were prepared using whole‐transcriptome analysis and TCR/BCR amplification kits, respectively. The libraries were quantified using a high‐sensitivity DNA chip (Agilent Technologies) on a Bioanalyzer 2200 and Qubit high‐sensitivity DNA assay (Thermo Fisher Scientific). Sequencing was performed using Novaseq6000 (Illumina, San Diego, CA, USA) on a 150 bp paired‐end run.

The scRNA‐seq data analysis was performed by NovelBio Bio‐Pharm Technology Co., Ltd. using the NovelBrain Cloud Analysis Platform. BD Rhapsody sequence analysis pipeline was applied for single cell transcriptome analysis and V(D)J analysis along with mouse genome mm10 (ensembl 100). Cells containing over 200 expressed genes and mitochondrial UMI rates below 20% passed the cell quality filter, and mitochondrial genes were removed from the expression table. The Seurat package (version: 4.0.3, https://satijalab.org/seurat/) was used for cell normalization and regression based on the expression table according to the UMI counts of each sample and the percentage of mitochondria to obtain scaled data. PCA was constructed based on scaled data with the top 2000 highly variable genes, and the top 10 principal genes were used for tSNE and UMAP construction.

Utilizing the graph‐based cluster method (resolution = 0.8), the unsupervised cell cluster results were acquired based on the top 10 principal components of PCA, and the marker genes were calculated using the FindAllMarkers function with the Wilcoxon rank sum test algorithm under the following criteria:1. Log2FC > 0.25; 2. *p* value < 0.05; 3. min.pct > 0.1. Clusters of the same cell type were selected for sub‐cluster analysis to identify the cell type in detail.

Single‐cell trajectory analysis was performed using Monocle2 (http://cole‐trapnell‐lab. github. io/monocle‐release) with DDR‐Tree and default parameters. Before Monocle analysis, marker genes were selected from the Seurat clustering results and raw expression counts of the filtered cells. Based on pseudo‐time analysis, branch expression analysis modeling (BEAM Analysis) was applied for branch fate‐determined gene analysis.

To assess the strength of transcription factor regulation, the single‐cell regulatory network inference and clustering (pySCENIC, v0.9.5) (Aibar et al., 2017) was applied workflow using the 20‐thousand motifs database for RcisTarget and GRNboost.

### QuSAGE Analysis (Gene Enrichment Analysis) and Cell Communication Analysis

To characterize the relative activation of a given gene set, such as pathway activation, a QuSAGE (2.16.1) analysis was performed. To enable the systematic analysis of cell–cell communication molecules, cell communication analysis was applied based on CellPhoneDB, a public repository of ligands, receptors, and their interactions. Membrane, secreted, and peripheral proteins in the cluster were annotated at different time points. Significant means and Cell Communication significance (*p* < 0.05) were calculated based on the interaction and normalized cell matrix achieved by Seurat Normalization.

### TCR and BCR Analysis using Single Cell Sequencing

Quality control and read mapping were performed using the BD Rhapsody WTA analysis pipeline, which generated a normalized WTA matrix and adaptive immune receptor repertoire. For the TCR and BCR analyses, cells with the same CDR3 amino acid sequence were combined and identified as having the same clonotype. The size of each clonotype was calculated. All the original TCR and BCR sequencing data were uploaded to the NCBI public website GEO: ticket# 24748020 (GEO submission: GSE273375).

### Measuring Expression of Featured Markers in Peripheral Immune Cells of Treated Mice

Because PBMC isolated from mouse blood were not sufficient to perform this experiment, splenocytes were used in this study. Mice bearing subcutaneous B16F10 tumors were treated with PBS or NP31. Splenocytes isolated from healthy mice, PBS‐treated tumor‐bearing mice (day 20 post‐tumor inoculation), NP31‐treated tumor‐bearing mice (day 25 post‐tumor inoculation), and NP31‐cured tumor‐bearing mice (day 25 post‐tumor inoculation) were investigated. Splenocytes, either co‐incubated with nanoparticles loaded with whole tumor tissue lysates (0.8 mg mL^−1^, 48 h) or without co‐incubation with NPs, were collected and stained with the Zombie Aqua Fixable Viability Kit to distinguish live/dead cells. The samples were then incubated with the Fc Block, followed by staining with anti‐mouse antibodies against different surface markers (CD3, CD4, CD8, B220, KLRG1, CX3CR1, S1PR5, or IL2Ra). The splenocytes were then fixed, permeabilized using the Transcription Factor Buffer Set, and stained for intracellular S100A4 and ikzf2. antibody‐labeled cells were analyzed by flow cytometry.

### Measuring Featured Markers in PBMC of Patients with Lung Cancer in Prior‐ or Ongoing Immunotherapy

Fresh blood (5–8 mL) was collected using sodium heparin anticoagulant tubes from patients with non‐small lung cancer, prior‐ or ongoing immunotherapy (αPD‐1 antibody + Cisplatin, day 42). The sample was diluted with an equal volume of PBS, followed by the addition of Ficoll separation medium and centrifugation at room temperature. After centrifugation, the PBMC layer (white membrane) was collected. PBMC, either co‐incubated with nanoparticles loaded with whole tumor tissue lysates (0.8 mg mL^−1^, 48 h, AIM V culture medium) or without co‐incubation with NPs, were collected and stained with the Zombie Aqua Fixable Viability Kit to distinguish live/dead cells. The samples were then incubated with the Fc Block, followed by staining with anti‐human antibodies against different surface markers (CD3, CD4, CD8, KLRG1, CX3CR1, or IL2Ra). Splenocytes were then fixed, permeabilized using the Transcription Factor Buffer Set, and stained for intracellular S100A4 and S100A8. antibody‐labeled cells were then analyzed by flow cytometry (FACS AriaTM III, FlowJo 10).

### Statistical Analysis

All statistical analyses were performed using GraphPad Prism software version 8.3.0 and R software version 4.1. Data were analyzed using the *t*‐test and chi‐square test based on the data type and normality distribution. The error bars represent the standard deviation (SD) or standard error of the mean (SEM). Statistical *p*‐value < 0.05 was considered statistically significant: ^*^ *p* < 0.05, ^**^ *p* < 0.01, ^***^ *p* < 0.001. Unless otherwise stated, asterisks indicate statistical comparisons with the control group.

## Conflict of Interest

The authors declare no conflict of interest.

## Author Contributions

X.X., L.D., J.W., and A.Z. contributed equally to this study. M.L. conceived and designed the study. M.L., L.D., and A.Z. prepared the NV and MV. L.D., X.X., A.Z., J. W., X.C., and Y.Z. conducted animal studies and flow cytometry studies. M.L. and L.D. conducted the single‐cell sequencing experiments. M.L., L.D., K.H., J.Z., C.D., C.L., J.Z., and Y.P. analyzed the data and drawn the figures. M.L. wrote and revised the manuscript. All authors reviewed and approved the final version of the manuscript.

## Supporting information



Supporting Information

## Data Availability

The data that support the findings of this study are available from the corresponding author upon reasonable request.
