# Supplementary material for: Maximizing Efficacy of Cancer Nanovaccines and Immune Cells Landscape in Responders and Non‐Responders to Immunotherapy
Source: Adv Sci (Weinh). 2025 Jun 26;12(35):e16756. doi: 10.1002/advs.202416756 (PMC12463128; doi:10.1002/advs.202416756)
Supplement: Supplementary file 1 — Supporting Information [file ADVS-12-e16756-s001.pdf]

ADVANCED SCIENCE
Open Access

Supporting Information

for *Adv. Sci.*, DOI 10.1002/advs.202416756

Maximizing Efficacy of Cancer Nanovaccines and Immune Cells Landscape in Responders and Non-Responders to Immunotherapy

Xiangxiang Xu, Lu Diao, Jin Wang, Ao Zhu, Xianlan Chen, Yan Zheng, Yuhan Liu, Kang Hu, Jiashan Zhu, Cheng Ding, Chang Li, Yunzhi Pan, Jun Zhao and Mi Liu**

Supporting Information

Maximizing efficacy of cancer nanovaccines and immune cells landscape in responders and non-responders to immunotherapy

Xiangxiang Xu[1,2#], Lu Diao[1,2,3,4#], Jin Wang[1,2#], Ao Zhu[1,2#], Xianlan Chen[1,2], Yan Zheng[1,2], Yuhan Liu[1,2], Kang Hu[2,5,6], Jiashan Zhu[2,5,6], Cheng Ding[2,5,6], Chang Li[2,5,6], Yunzhi Pan[7], Jun Zhao[2,5,6]*, Mi Liu[1,2,3,4,8]*

[1] Department of Pharmaceutics, College of Pharmaceutical Sciences, Soochow University, Suzhou, 215123, People's Republic of China

[2] Institute of Minimally Invasive Thoracic Cancer Therapy and Translational Research, Soochow University, Suzhou, Jiangsu, 215123, People's Republic of China

[3] Ersheng Biopharmaceutical Co., Ltd., Suzhou, Jiangsu, 215000, People's Republic of China

[4] Wuxi Boston Biopharmaceutical Co., Ltd., Wuxi, 214125, People's Republic of China

[5] Institute of Thoracic Surgery, The First Affiliated Hospital of Soochow University, Soochow University, Suzhou, Jiangsu, 215123, People's Republic of China

[6] Department of Thoracic Surgery, The First Affiliated Hospital of Soochow University, Soochow University, Suzhou, Jiangsu, 215123, People's Republic of China

[7] Department of Pharmacy, The affiliated infectious Diseases Hospital of Soochow University Suzhou 215000, P. R. China

[8]Jiangsu Province Engineering Research Center of Precision Diagnostics and Therapeutics Development, Soochow University, Suzhou 215123, China, People's Republic of China.

# Contribute equally to this study

*Corresponding author, Mi Liu, email: mi.liu@suda.edu.cn

Jun Zhao, email: Junzhao@suda.edu.cn

Table S1

NPs	Tumor tissue	Tumor tissue freezing	Fixation	Lysis	Solubilization solution	Heating	Antigen purification	Adjuvants	Oxidation
NP1	B16F10	√	×	Freezing+thawing	Urea	×	×	Poly(I:C)	×
NP2	B16F10	√	×	Freezing+thawing	Urea	×	×	BcG	×
NP3	B16F10	√	×	Freezing+thawing	Urea	×	×	$MnCl_2$	×
NP4	B16F10	√	×	Freezing+thawing	Urea	×	×	Poly(I:C) + $MnCl_2$	×
NP5	B16F10	√	×	Freezing+thawing	Urea	×	×	Poly(I:C) + $CaCl_2$	×
NP6	B16F10	√	×	Freezing+thawing	Urea	×	×	Poly(I:C)+ Colloid manganese	×
NP7	B16F10	√	×	Freezing+thawing	Urea	×	×	Colloid manganese	×
NP8	B16F10	√	×	Freezing+thawing	Urea	×	×	CpG1018+CpG1826	×
NP12	B16F10	√	×	Freezing+thawing	Urea	×	×	Poly(I:C)+CpG1018+CpG1826	×
NP13	B16F10	√	×	Freezing+thawing	Urea	×	×	Poly(I:C)+GM-CSF	×
NP25	B16F10	√	×	Freezing+thawing	Urea	×	×	Poly(I:C)+CpG1018	×
NP31	B16F10	√	×	Freezing+thawing	Urea	×	×	Poly(I:C)+CpG1018+CpG2395	×
NP32	B16F10	√	×	Freezing+thawing	Urea	×	×	Poly(I:C)+CpG1018+CpG2006	×
NP36	B16F10	√	×	Freezing+thawing	Urea	×	×	Poly(I:C)+CpG1585+CpG2395	×
NP39	B16F10	√	×	Freezing+thawing	Urea	×	×	Poly(I:C)+CpG2216+CpG1585	×
NP77	B16F10	√	×	Freezing+thawing	Sodium deoxycholate	×	×	Poly(I:C)+CpG1018+CpG2395	×
NP78	B16F10	√	×	Freezing+thawing	GdnHCl	×	×	Poly(I:C)+CpG1018+CpG2395	×
NP83	B16F10	√	×	Freezing+thawing	Urea	×	×	Poly(I:C)+CpG1018+CpG2395	×
NP97	B16F10	√	×	Freezing+thawing	Metformin	×	×	Poly(I:C)+CpG1018+CpG2395	×
NP99	B16F10	√	×	Freezing+thawing	Urea	×	Salting out	Poly(I:C)+CpG1018+CpG2006	×
NP113	B16F10	√	×	Urea	Urea	×	Salting out	Poly(I:C)+CpG1826+CpG2395	×
NP114	B16F10	√	×	GdnHCl	GdnHCl	×	Salting out	Poly(I:C)+CpG1826+CpG2395	×
NP126	B16F10	√	×	Freezing+thawing	Urea	×	×	Poly(I:C)+CpG1018+CpG2395	×
NP127	B16F10	√	×	Freezing+thawing	Urea	×	×	Poly(I:C)+CpG1018+CpG2395	×
NP128	B16F10	√	×	Freezing+thawing	Urea	×	×	Poly(I:C)+CpG1018+CpG2395	×
NP129	B16F10	√	×	Urea	Urea	×	×	Poly(I:C)+CpG1018+CpG2395	HClO
NP140	B16F10	√	×	Freezing+thawing	Urea	×	Salting out	Poly(I:C)+CpG1018+CpG2395	×
NP144	B16F10	√	×	Urea	Urea	×	Salting out	Poly(I:C)+CpG1018+CpG2395	×
NP145	B16F10	√	×	GdnHCl	GdnHCl	×	Salting out	Poly(I:C)+CpG1018+CpG2395	×
NP152	B16F10	√	×	Urea	Urea	×	Salting out	Poly(I:C)+CpGSL03+CpG2395	×
NP153	B16F10	×	√(Paraformaldehyde)	Urea	Urea	×	×	Poly(I:C)+CpG1018+CpG2395	×
NP154	B16F10	×	√(Formalin)	Urea	Urea	×	×	Poly(I:C)+CpG1018+CpG2395	×
NP161	LLC	√	×	Urea	Urea	×	×	Poly(I:C)+CpG2006+CpG2395	×
NP164	B16F10	×	√(Ethanol)	Urea	Urea	×	×	Poly(I:C)+CpG1018+CpG2395	×
NP168	B16F10	×	√	Urea	Urea	×	Salting out	Poly(I:C)+CpG1018+CpG2395	×
NP169	B16F10	√	×	Urea	Urea	×	×	Poly(I:C)+CpG1018+CpG2395	×
NP182	LLC	√	×	Urea	Urea	×	Salting out	Poly(I:C)+CpG2006+CpG2395	×
NP187	B16F10	√	×	Urea	Urea	×	×	Poly(I:C)+CpG2006+CpG2395	H_2O_2
NP188	B16F10	×	√(Ethanol+H_2O_2)	Urea	Urea	×	×	Poly(I:C)+CpG1018+CpG2395	×
NP191	B16F10	×	√	Urea	Urea	√	Heating	Poly(I:C)+CpG1018+CpG2395	×
NP213	KPC	√	×	Urea	Urea	×	×	Poly(I:C)+CpG1018+CpG2395	×
NP218	KPC	√	×	Urea	Urea	×	Salting out	Poly(I:C)+CpG1018+CpG2395	×
NP230	KPC	√	×	Urea	Urea	×	Salting out	Poly(I:C)+CpG1018+CpG2395	×
NP231	KPC	√	×	Urea	Urea	×	Salting out	Poly(I:C)+CpG1018+CpG2395	H_2O_2
NP401	B16F10	×	√ (Glacial acetic acid)	Urea	Urea	×	×	Poly(I:C)+CpG1018+CpG2395	×
NP403	B16F10	×	√(Ethanol+HClO)	Urea	Urea	×	×	Poly(I:C)+CpG1018+CpG2395	×
NP406	B16F10	×	√(Ethanol)	Urea	Urea	×	Ethanol precipitate	Poly(I:C)+CpG1018+CpG2395	×

Table S2

NPs or MPs	Zeta Potential (mV, ±SD)	Size (nm, ±SD)	Loading capacity (μg/mg proteins)	NPs or MPs	Zeta Potential (mV, ±SD)	Size (nm, ±SD)	Loading capacity (μg/mg proteins)
Blank NP	-23.93±0.31	202.27±12.02	0	NP83(Water)	-23.23±0.86	935.16±40.76	102.44±4.56
Peptide NP	-26.20±0.30	338.50±6.78	99.76±3.71	NP83(Urea)	-29.03±1.17	805.83±92.10	103.64±3.13
NP1(Water)	-25.87±0.45	293.43±15.97	105.90±3.29	NP97(Water)	-26.13±0.57	271.36±6.84	98.06±5.79
NP1(Urea)	-22.77±0.40	280.20±3.44	102.58±3.86	NP97(Metformin)	-29.80±2.48	217.50±1.08	97.69±4.92
NP2(Water)	-21.90±0.75	300.20±2.95	101.22±4.05	NP99	-26.70±1.35	199.43±4.83	101.22±5.66
NP2(Urea)	-27.03±0.15	232.10±8.15	102.76±2.99	NP113	-22.70±0.78	280.20±3.44	103.88±4.27
NP3(Water)	-17.40±0.20	352.13±5.56	103.14±2.97	NP114	-21.07±0.47	214.23±5.30	104.55±4.83
NP3(Urea)	-21.63±0.31	259.63±12.68	102.99±3.33	NP126 (Water)	-28.23±1.01	5524.33±1016.07	101.99±3.88
NP4(Water)	-25.57±0.29	272.56±1.40	98.77±3.09	NP126 (Urea)	-22.53±1.33	5346.66±699.49	103.11±2.87
NP4(Urea)	-21.23±0.31	313.00±7.29	101.22±4.29	NP127(Water)	-27.73±0.31	2662.33±25.54	97.78±4.62
NP5(Water)	-22.63±0.75	342.43±6.50	97.66±3.26	NP127(Urea)	-27.40±0.60	2581.66±308.13	99.68±3.93
NP5(Urea)	-18.67±0.31	302.30±12.73	101.66±3.22	NP128 (Water)	-24.67±0.15	1190.00±104.31	104.25±4.08
NP6(Water)	-19.63±0.25	223.43±2.63	102.03±4.11	NP128 (Urea)	-19.30±0.60	1178.66±105.19	103.66±5.31
NP6(Urea)	-23.97±0.95	241.60±4.77	104.95±3.52	NP129	-18.23±0.75	176.56±3.52	102.98±3.77
NP7(Water)	-21.23±0.31	221.83±5.74	96.90±4.74	NP140 (Water)	-27.50±0.20	243.16±7.71	96.15±4.80
NP7(Urea)	-20.87±0.71	222.23±4.17	102.58±3.86	NP140 (Urea)	-25.97±0.32	214.90±5.68	105.38±3.68
NP8(Water)	-15.83±0.98	203.00±2.34	104.80±4.39	NP144	-22.43±0.21	214.90±5.68	102.55±4.06
NP8(Urea)	-18.03±1.07	228.93±3.87	104.12±2.08	NP145	-23.93±0.64	284.70±9.88	103.98±3.96
NP12(Water)	-24.50±4.51	244.30±20.01	99.07±4.81	NP152	-30.60±0.44	242.80±3.50	98.75±4.18
NP12(Urea)	-26.73±0.32	258.00±5.75	101.93±3.77	NP153	-29.93±0.40	287.46±14.39	102.98±3.79
NP13(Urea)	-25.37±0.49	230.86±4.11	102.66±5.34	NP154	-30.63±0.15	268.73±12.41	101.77±3.55
NP13(Water)	-17.77±0.64	225.43±1.36	104.99±3.55	NP161	-20.27±0.57	243.16±7.71	98.36±4.82
NP25(Water)	-20.53±0.90	332.23±8.86	101.67±4.52	NP164	-22.30±0.82	231.36±12.94	99.64±4.26
NP25(Urea)	-22.70±0.50	208.93±1.12	103.42±5.63	NP168	-27.07±0.93	248.06±23.38	101.71±5.03
NP31(Water)	-22.13±0.64	192.03±4.19	96.52±4.36	NP169	-20.09±0.10	198.10±11.87	102.58±3.86
NP31(Urea)	-25.80±1.41	210.46±2.59	102.88±3.69	NP182	-21.77±2.39	230.66±8.90	105.03±5.16
NP32(Water)	-25.63±0.45	232.03±6.53	104.27±3.84	NP187	-20.40±0.89	210.90±2.56	101.62±4.81
NP32(Urea)	-20.17±0.03	267.90±3.90	101.73±5.05	NP188	-24.30±1.25	221.33±7.37	104.32±3.04
NP36(Water)	-22.00±0.10	219.56±3.61	105.16±4.09	NP191	-22.57±0.38	226.53±8.29	102.99±5.11
NP36(Urea)	-18.90±0.30	219.73±1.00	102.67±3.07	NP213	-20.10±0.03	170.53±4.86	104.23±3.88
NP39(Water)	-21.77±0.64	232.73±10.93	103.89±4.96	NP218	-21.73±0.51	258.46±1.90	96.86±5.28
NP39(Urea)	-16.13±0.59	197.63±1.84	101.63±3.29	NP230	-18.43±1.45	212.93±6.47	101.55±3.60
NP77(Water)	-22.70±0.26	268.30±2.88	102.08±3.43	NP231	-18.73±1.50	265.23±30.84	105.13±3.05
NP77(Sodium deoxycholate)	-20.22±0.18	198.40±3.08	97.33±4.56	NP401	-20.23±0.50	261.30±12.98	101.48±5.15
NP78(Water)	-19.76±0.44	242.30±10.26	103.68±5.21	NP403	-26.80±0.53	289.13±60.36	103.22±4.08
NP78(Guanidine)	-22.40±0.36	250.23±4.68	105.24±4.37	NP406	-16.63±1.01	217.50±1.08	96.33±3.49

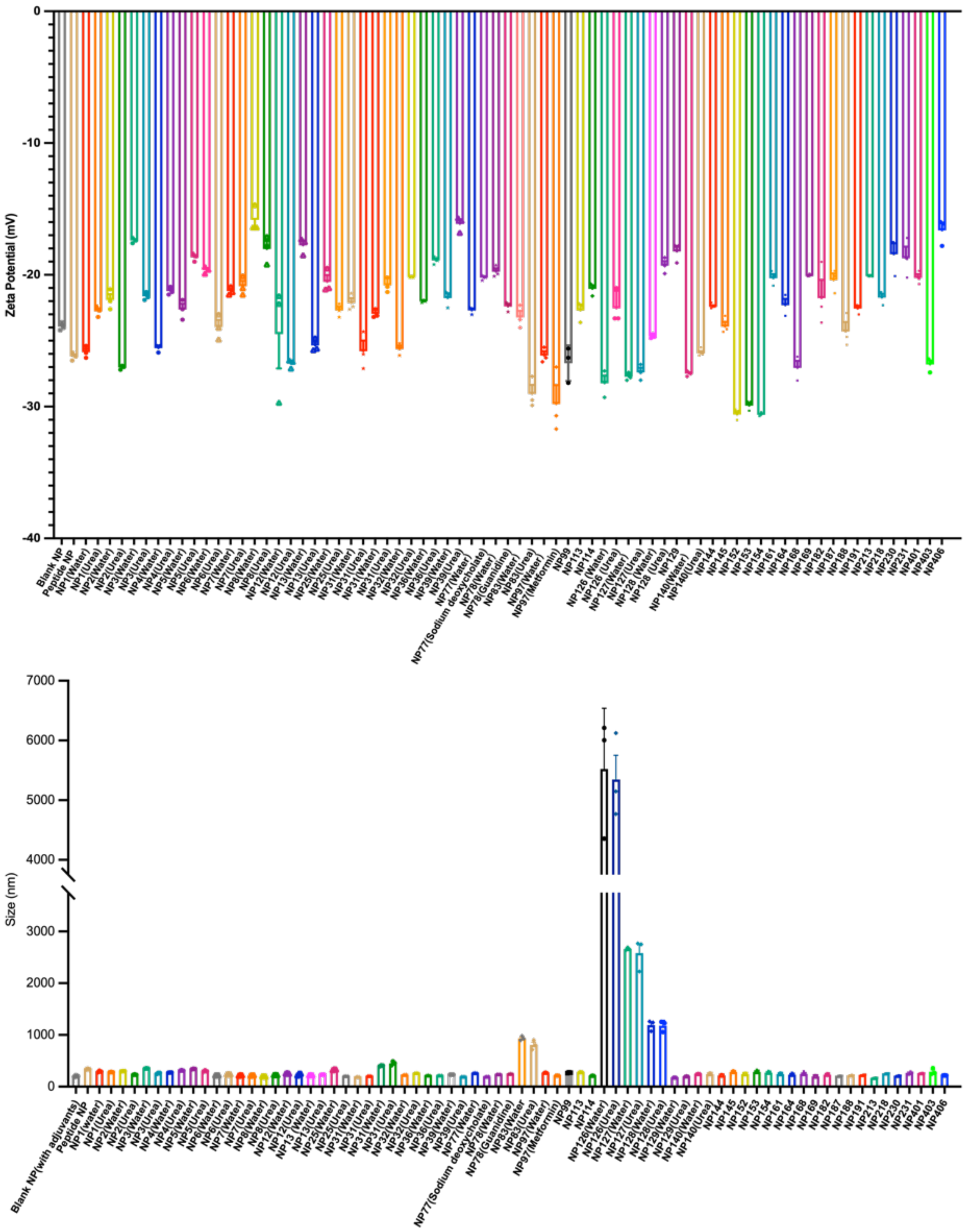

Figure S1. Sizes and zeta potentials of different nanoaprticles (NPs) or micronparticles. (MPs)

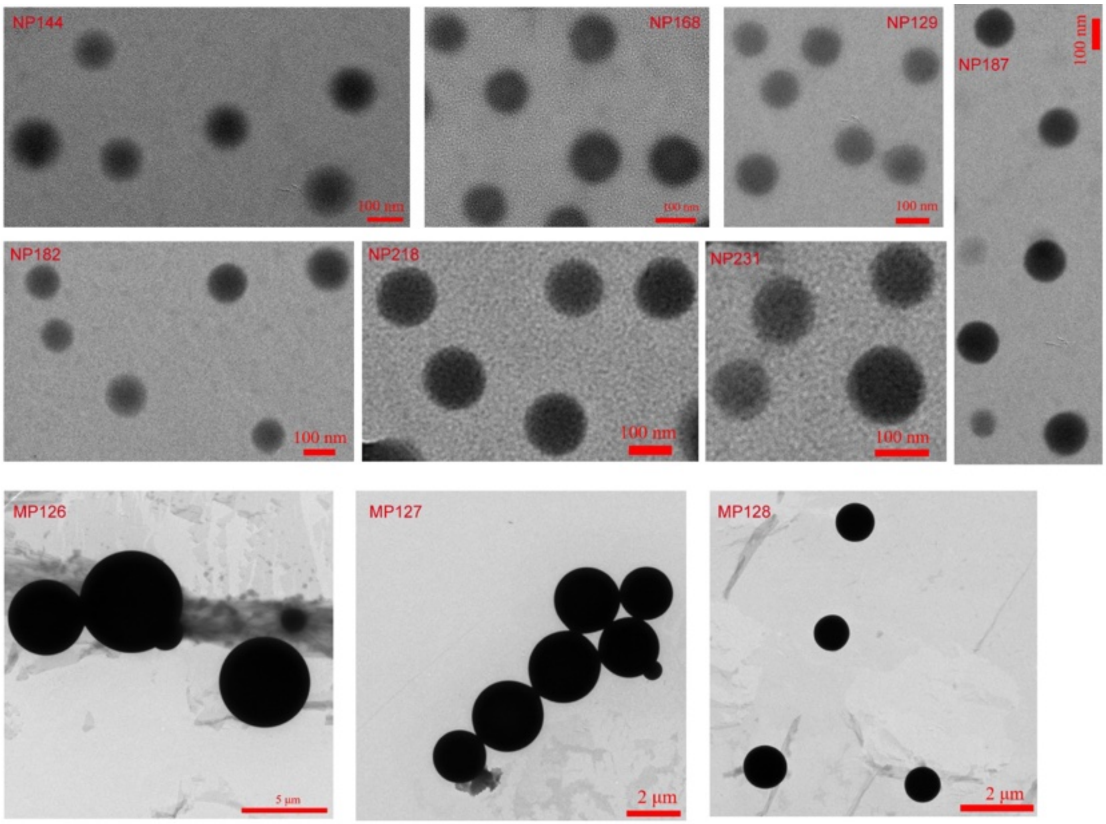

Figure S2. Morphological analysis of representative NPs or MPs by using transmission electron microscope (TEM).

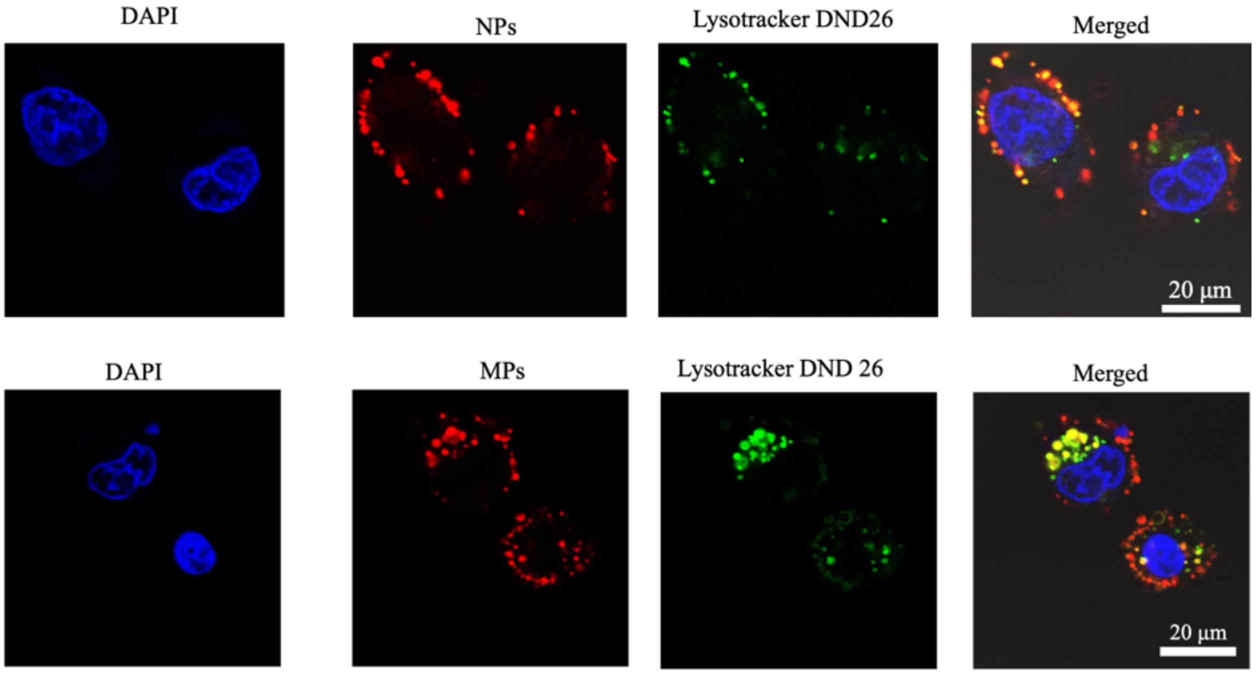

Figure S3. Investigation of NP (200nm) or MP (2.5μm) uptake by dendritic cells (DC) and antigen escape from endosome-lysosome system by using CONFOCAL.

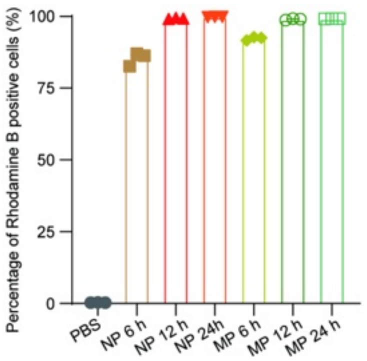

Figure S4. Rhodamine B-labeled NPs/MPs (0.5mg/ml) were incubated with DC2.4 cells for 0, 6, 12, 24 h, and the percentage of positive cells was determined by flow cytometry.

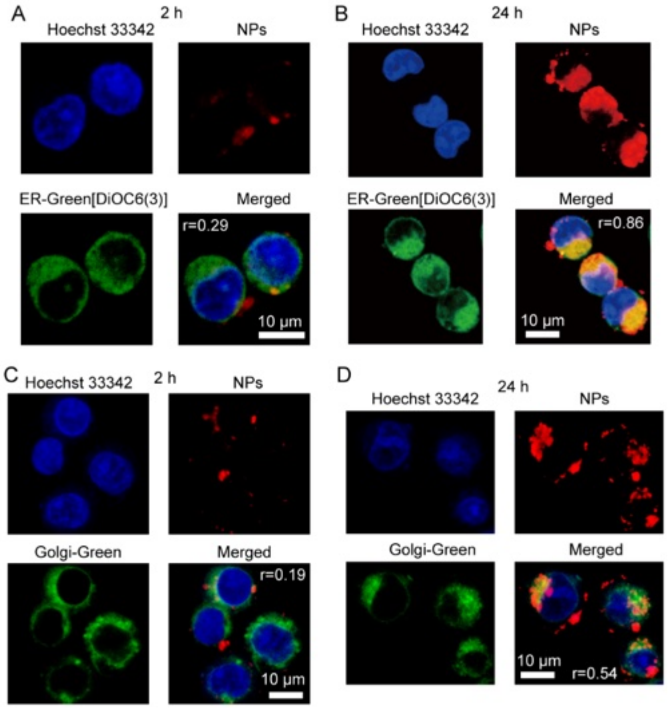

Figure S5. Confocal laser scanning microscopy revealed that the nanoparticles could escape to the endoplasmic reticulum and Golgi apparatus after incubation with DC2.4 cells for different timepoints (2h and 24h).

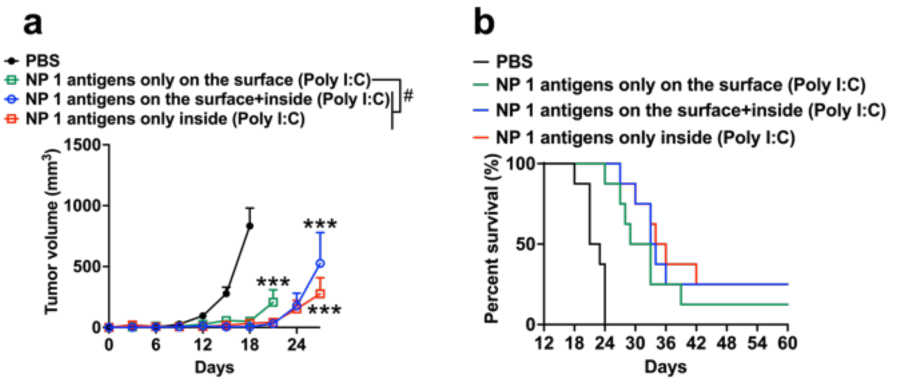

Figure S6. Investigation of the impact of antigen loading site on therapeutic efficacy of nanovaccines. a and **b**, Tumor growth curves and survival curves in melanoma-bearing mice treated with nanovaccines loading antigens at different sites. Data are presented as Mean ± SEM, P-values <0.05 were considered significant: **P<0.01, ***P<0.001.

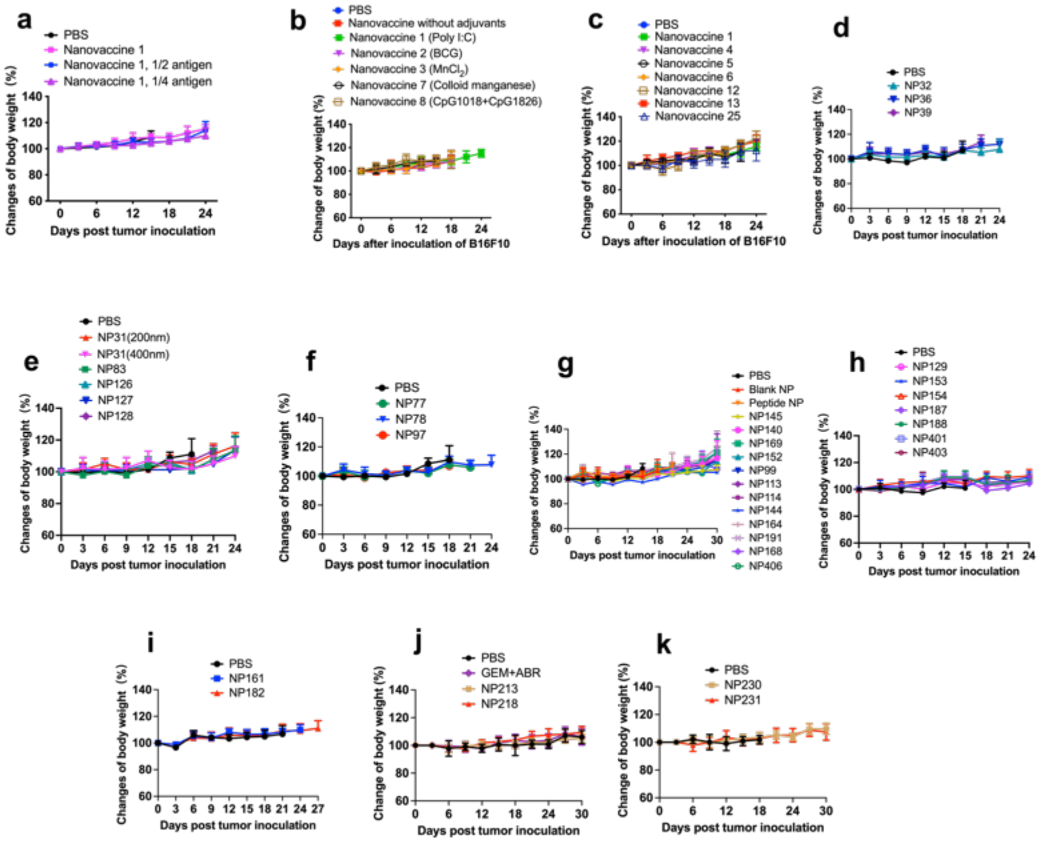

Figure S7. Analysis of potential toxicity of nanovaccines or micronvaccines by monitoring body weight changes of tumor-bearing and vaccine-treated mice. a-k, Boday weight changes of tumor-bearing mice treated with different nanovaccines or micronvaccines. Data are presented as Mean ± SD, no significant difference was observed among different groups.

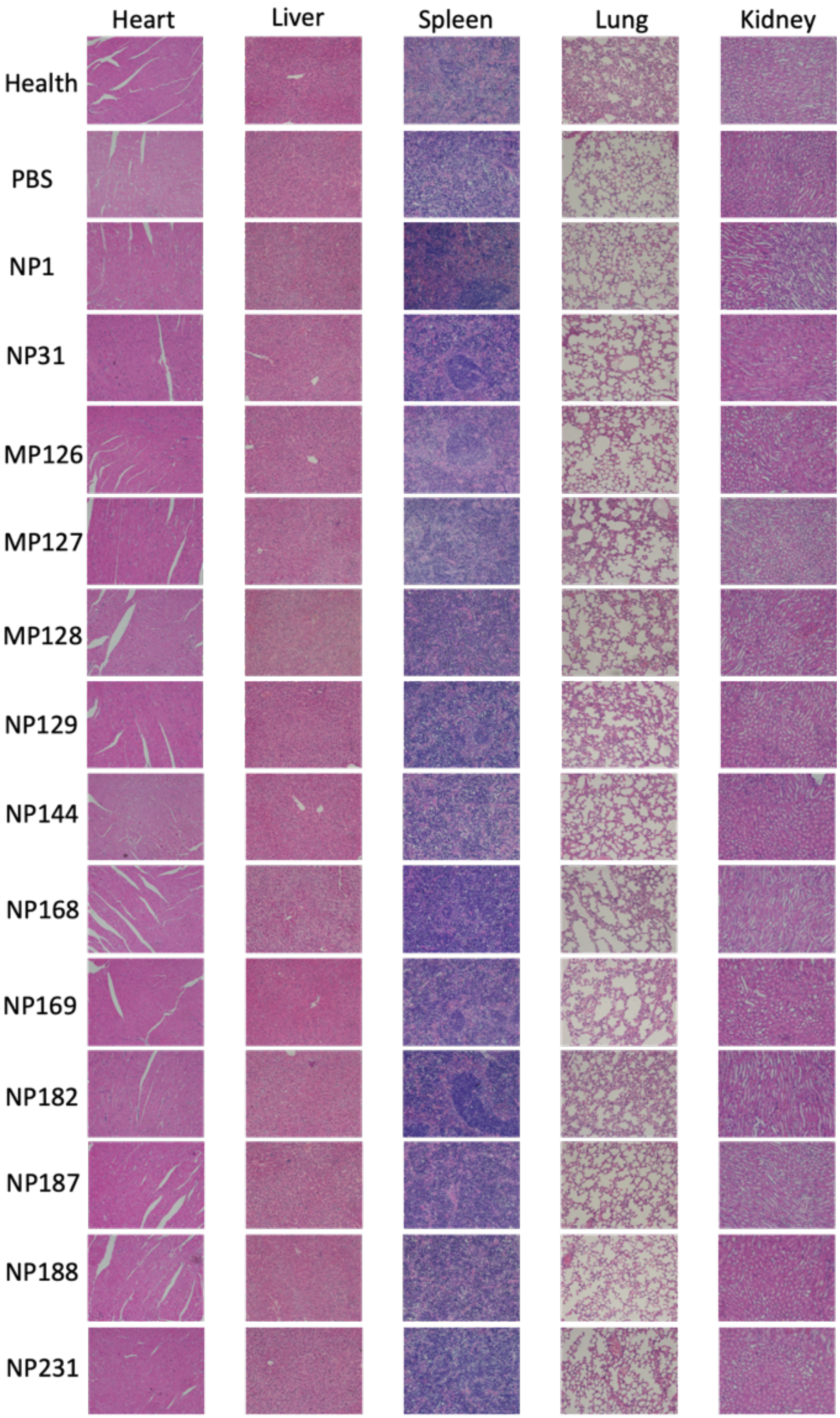

Figure S8. The results of H&E study conducted on sample of heart, live, spleen, lung and kidney from mice treated with MVs or NVs.

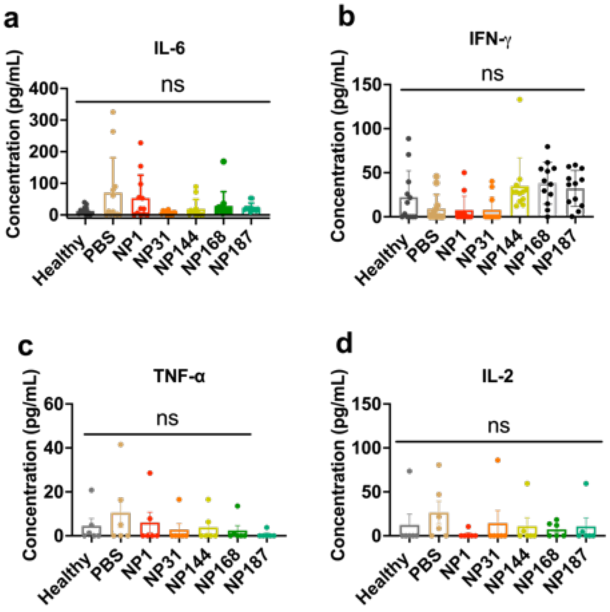

Figure S9. Analyisi of potential cytokine storms after vaccine treatment by measuring level of pro-inflammtory cytokines in blood of tumor-bearing and vaccine-treated mice. a-d, Changes of IL-6, INF-γ, TNF-α, and IL-2 in blood of tumor-bearing mice treated with different nanovaccines. Data are presented as Mean ± SD, no significant difference was observed among different groups.

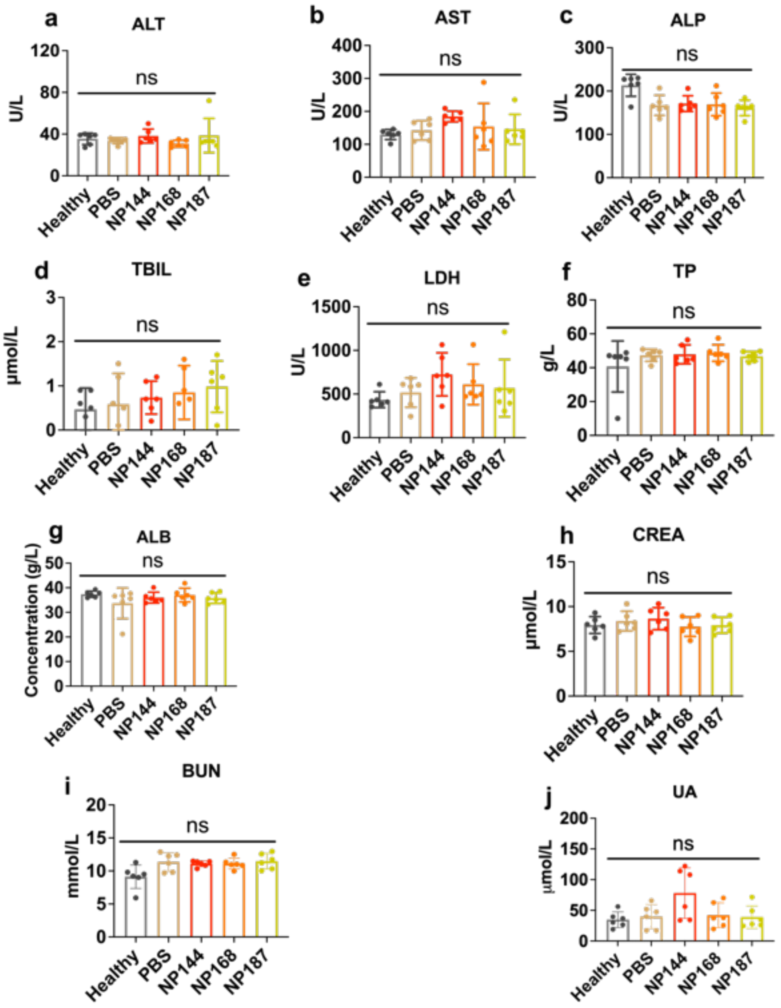

Figure S10. Analyisi of potential toxicities of nanovaccines by measuring various biochemical indicators in blood of tumor-bearing and vaccine-treated mice. a-j, Changes of biochemical indicators in blood of tumor-bearing mice treated with different nanovaccines or micronvaccines. Data are presented as Mean ± SD, no significant difference was observed among different groups.

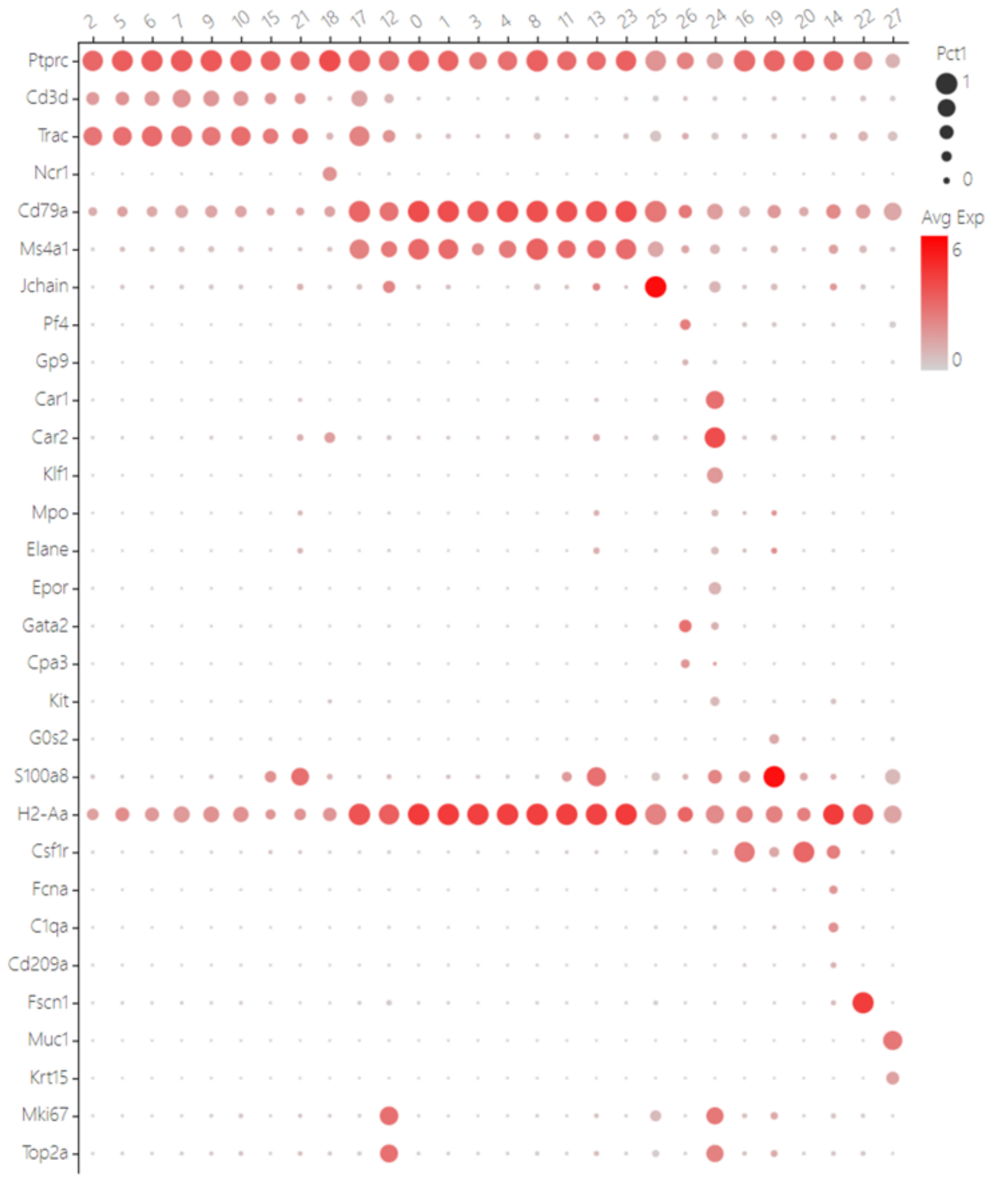

Figure S11. Some featured markers to identify clusters 0 to 27 in primary cell clustering analysis of single cell sequencing study.

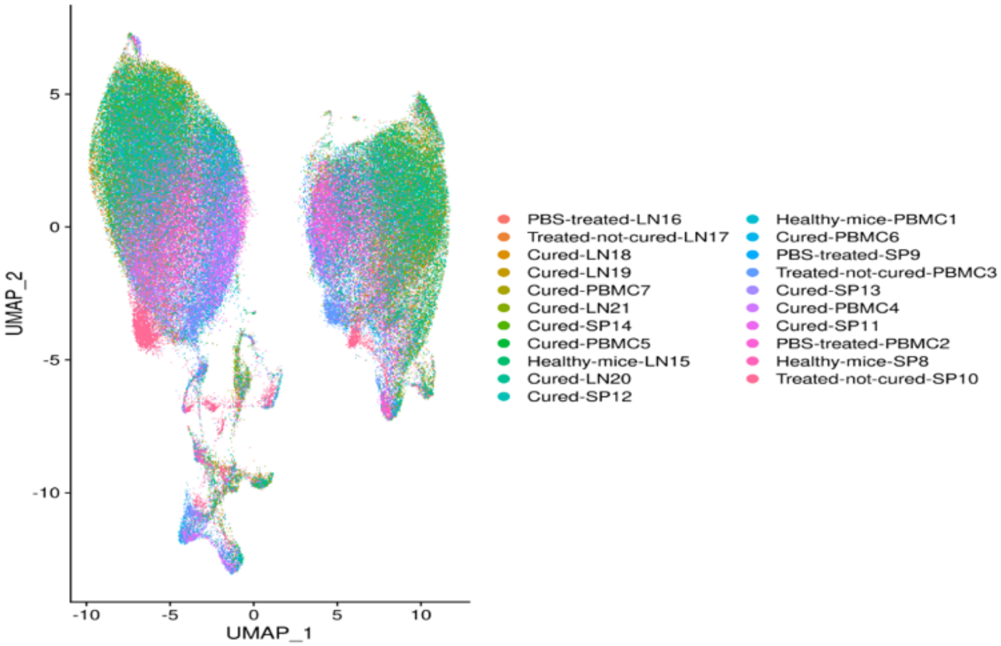

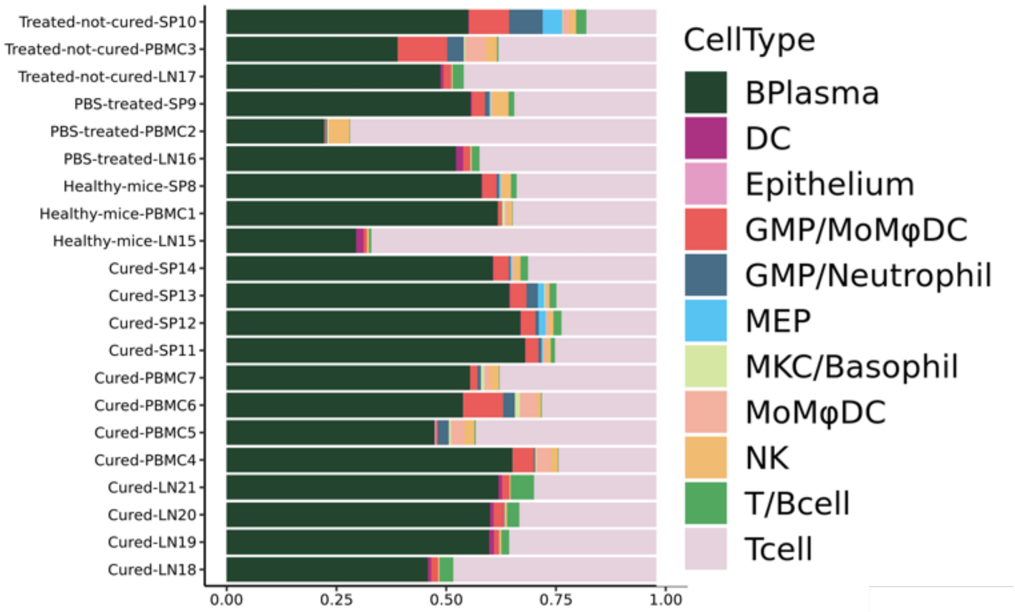

Figure S12. Distributions of different samples in 28 different clusters and distribution of different cell types in 21 samples in primary cell clustering analysis of single cell sequencing study.

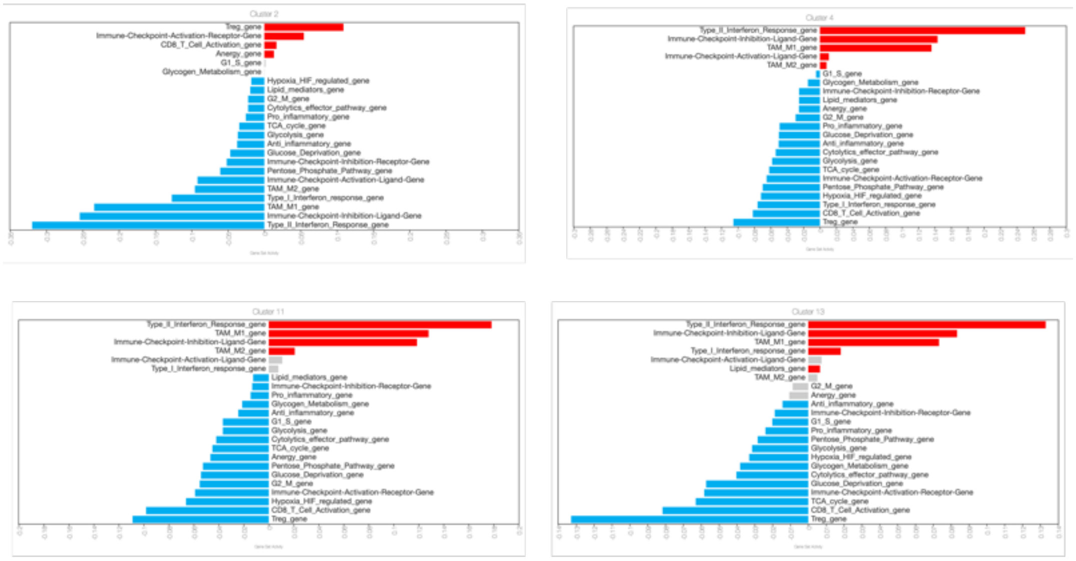

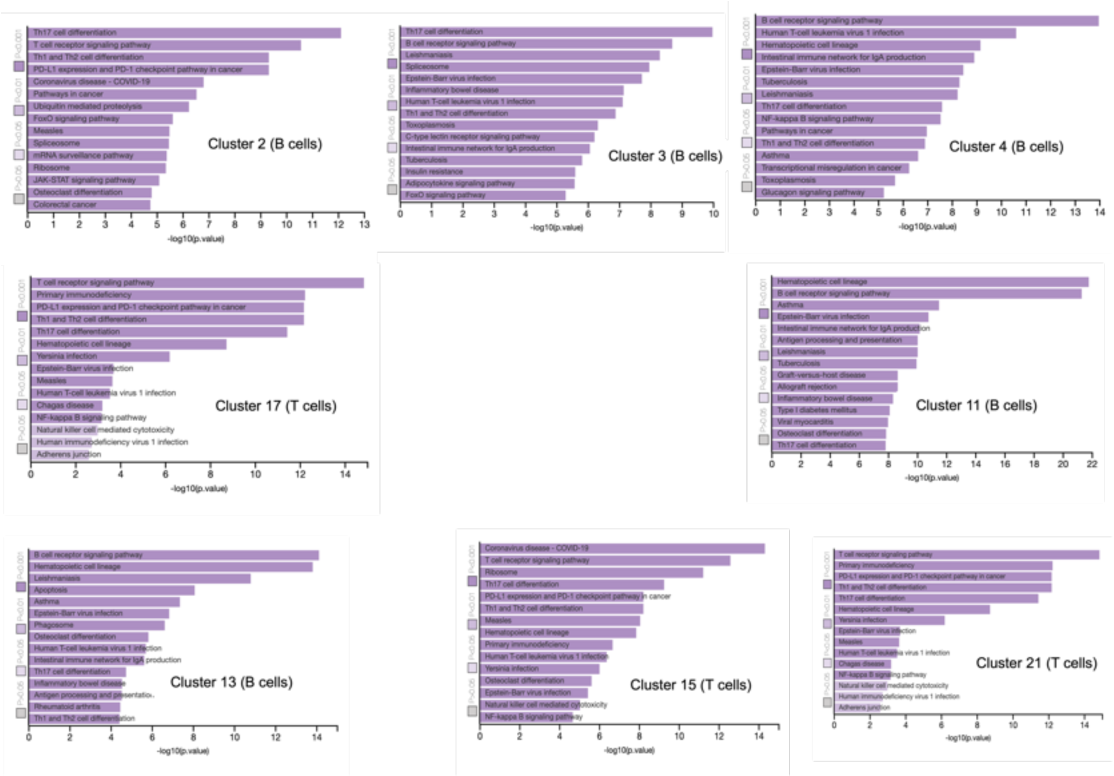

Figure S13. The gene set activity of clusters (cluster 2, cluster 4, cluster 11 and cluster 13) solely or highly exist in non-cured mice, and pathway analysis of different featured clusters in cured mice (clusters 2, 3, 4, 17) or non-cured (clusters 11, 13, 15, 21).

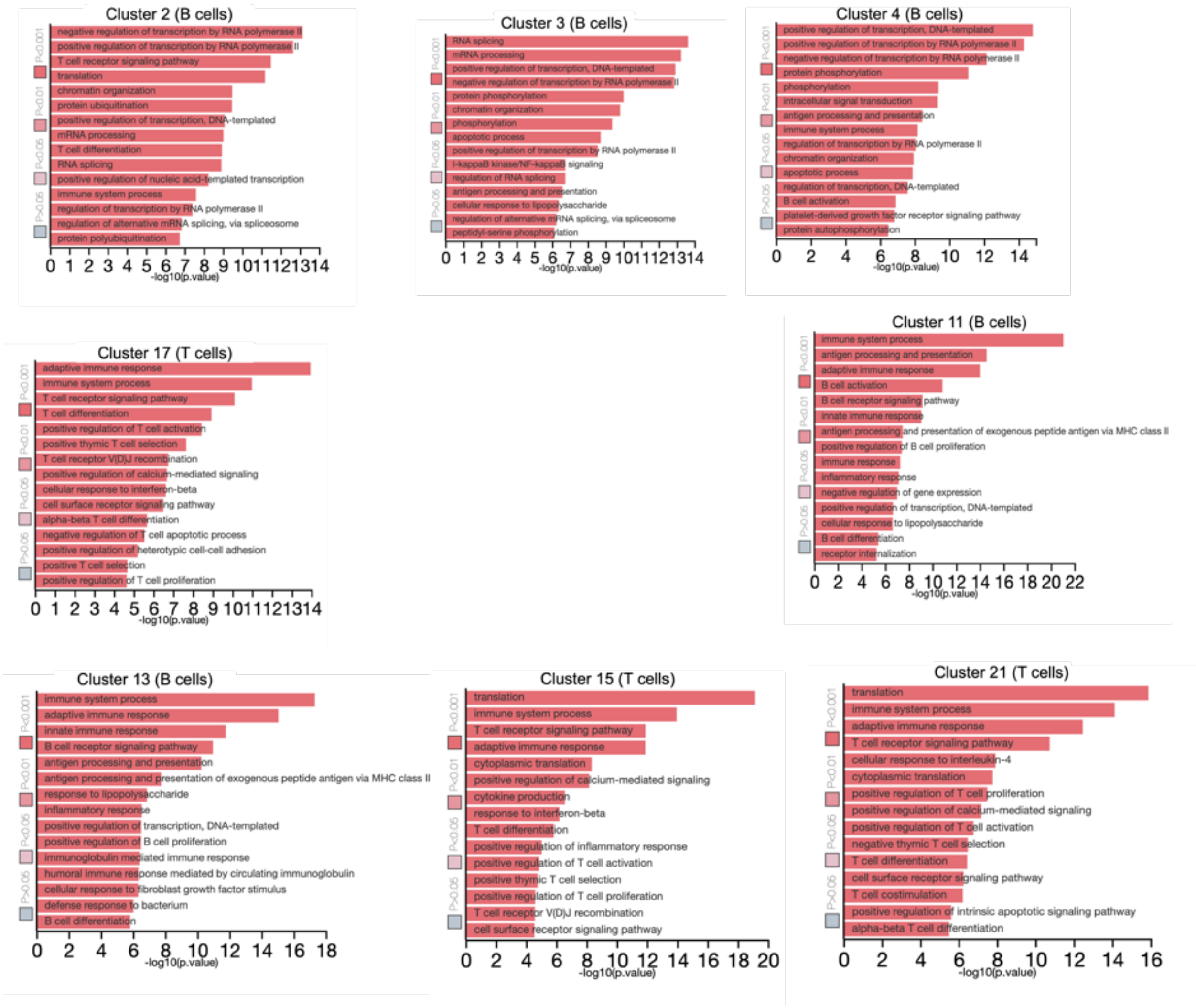

Figure S14. The function analysis of different featured clusters in cured mice (clusters 2, 3, 4, 17) or non-cured (clusters 11, 13, 15, 21).

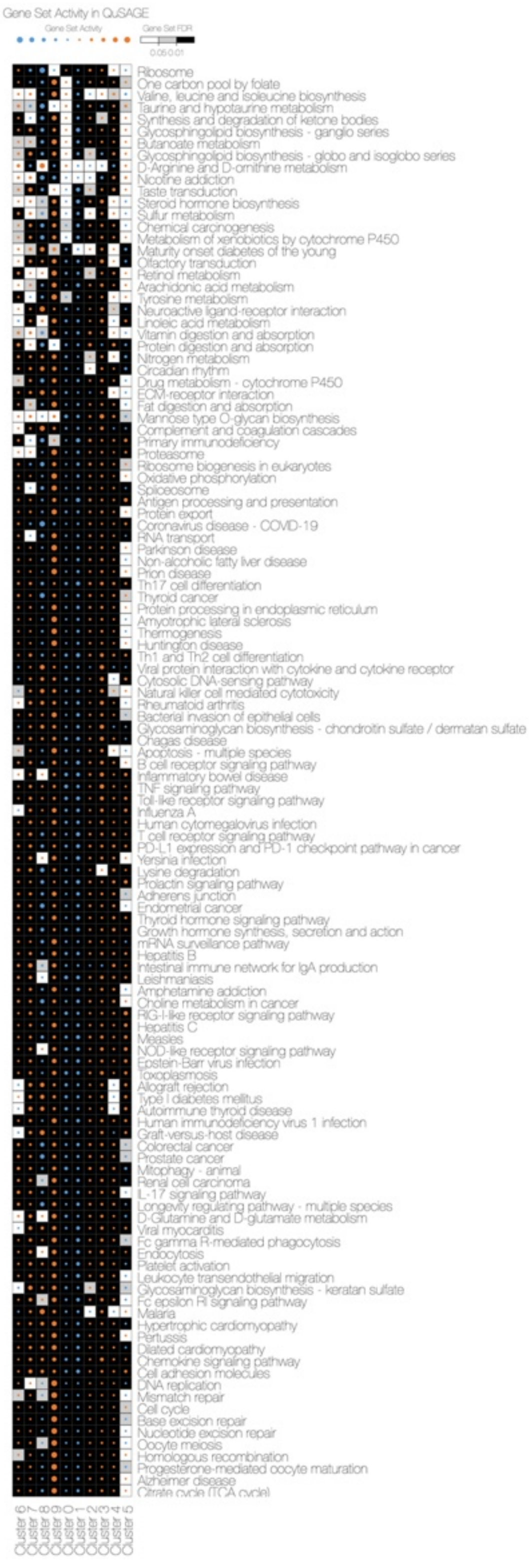

Figure S15. Gene Set Activity in QuSAGE in CD8+ T cell subclusters

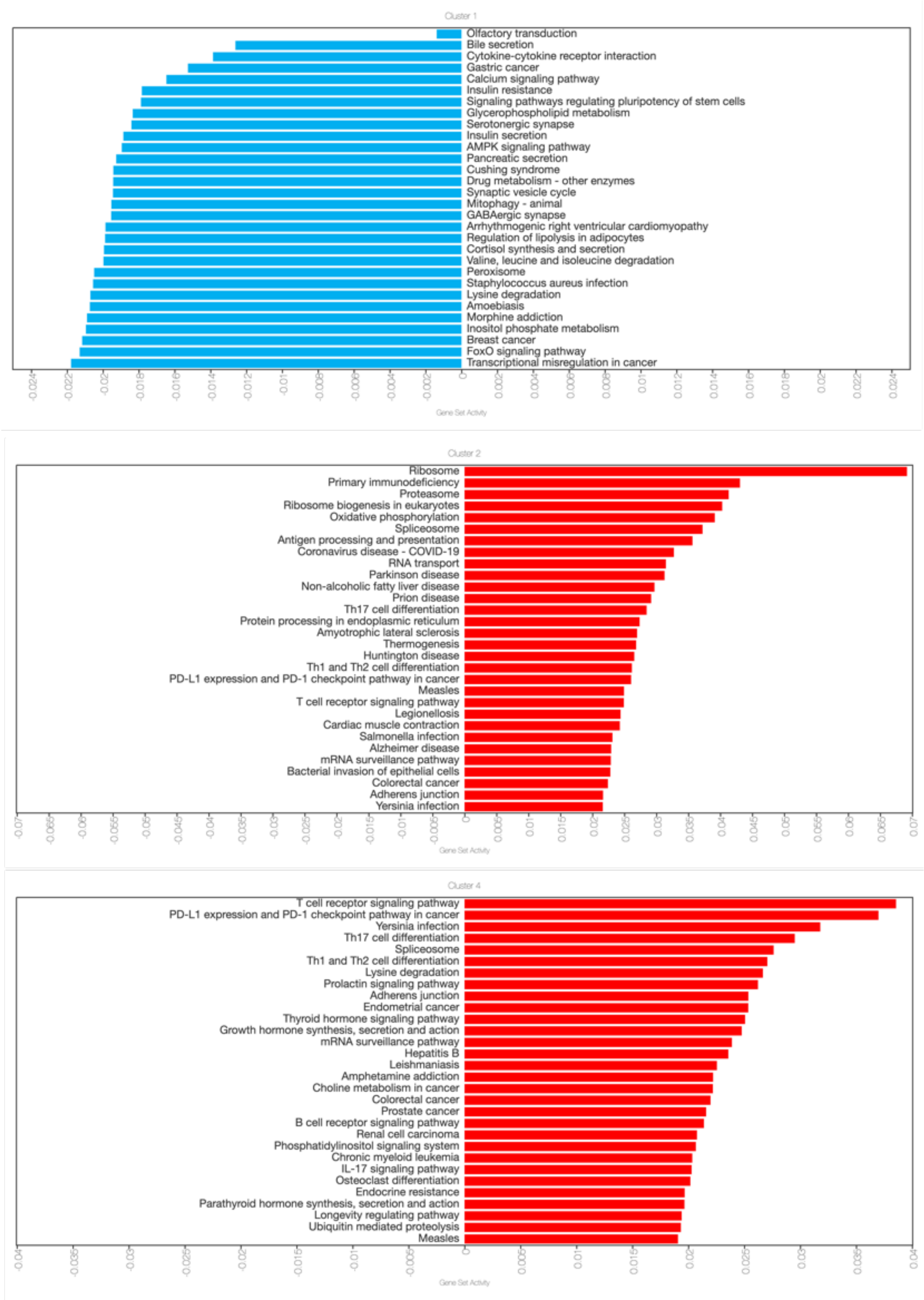

Figure S16. The gene set activity of clusters (cluster 1, cluster 2 and cluster 4), that have higher amounts in cured mice and healthy mice in $CD8^+$ T cell subclusters.

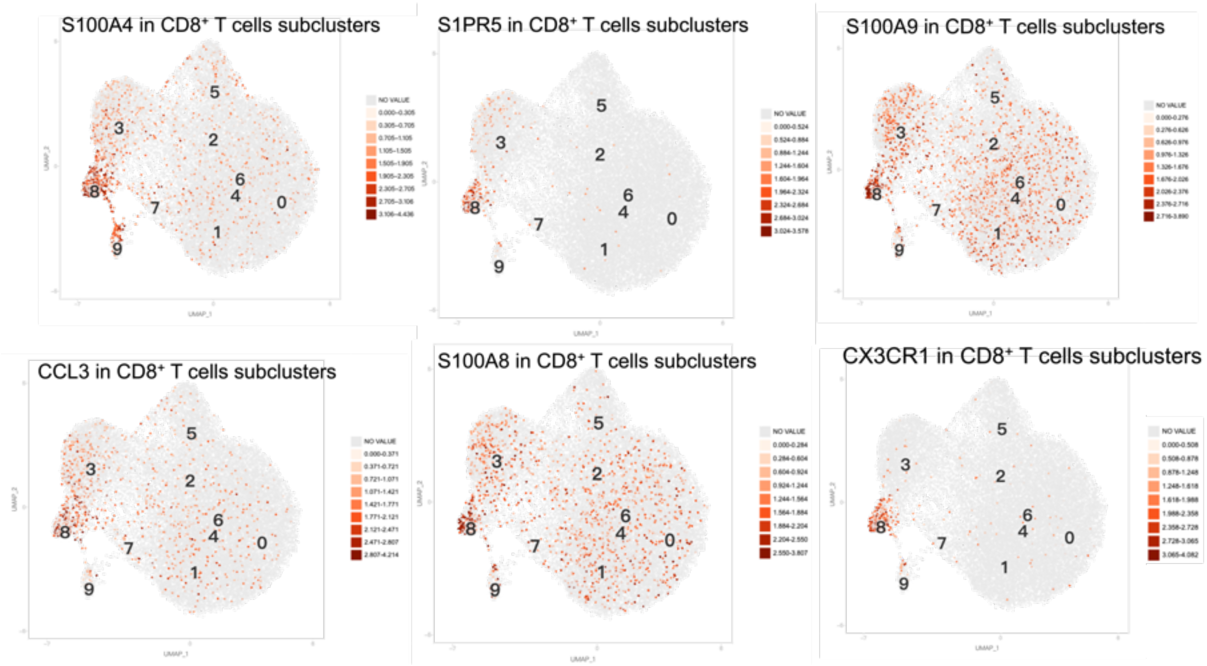

Figure S17. The expression of featured markers in different sub-clusters of $CD8^+$ T cells.

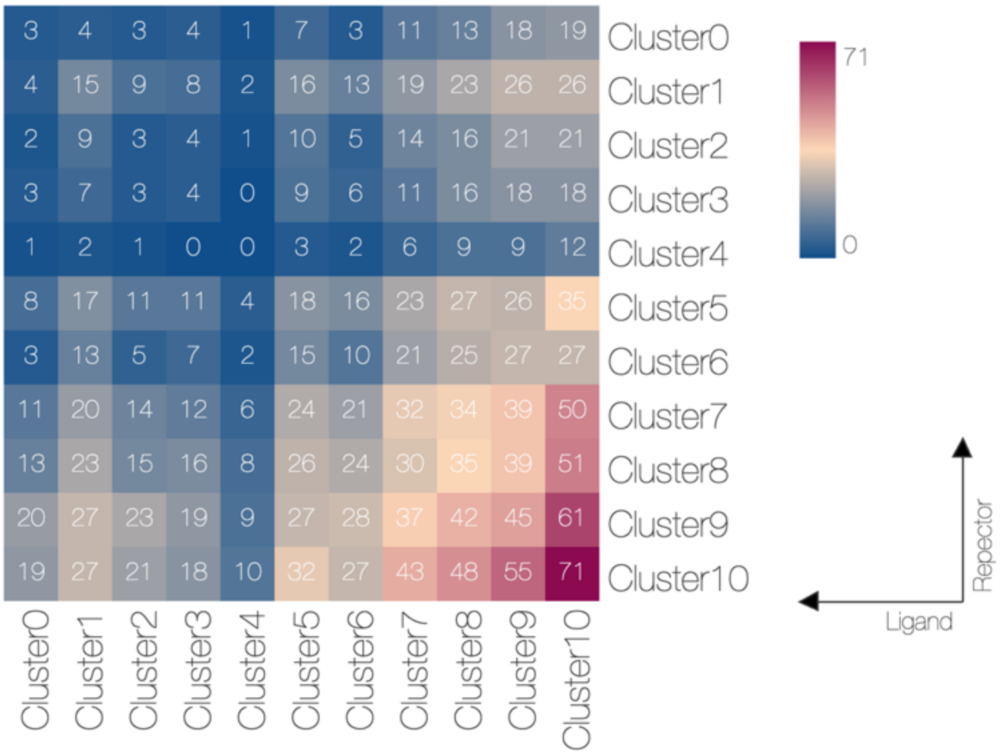

Figure S18. The cell communications among different CD4^{+} T cell subclusters.

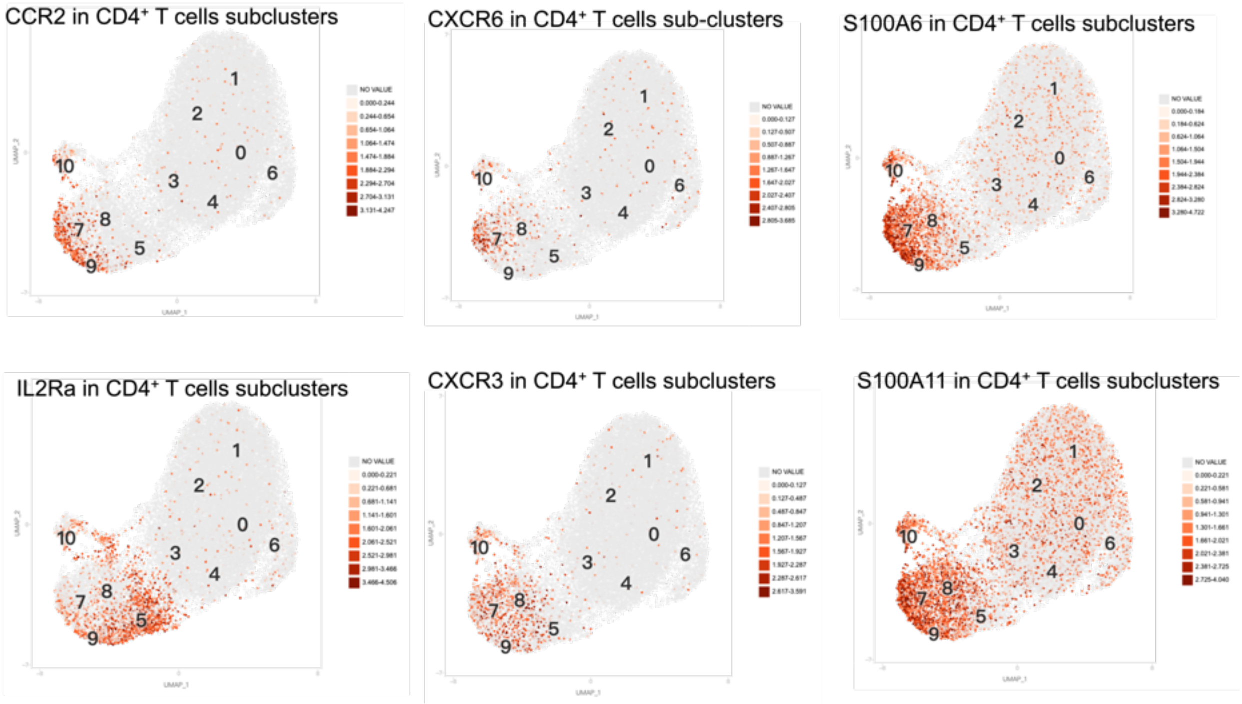

Figure S19. The expression of featured markers in different sub-clusters of CD4$^+$ T cells.

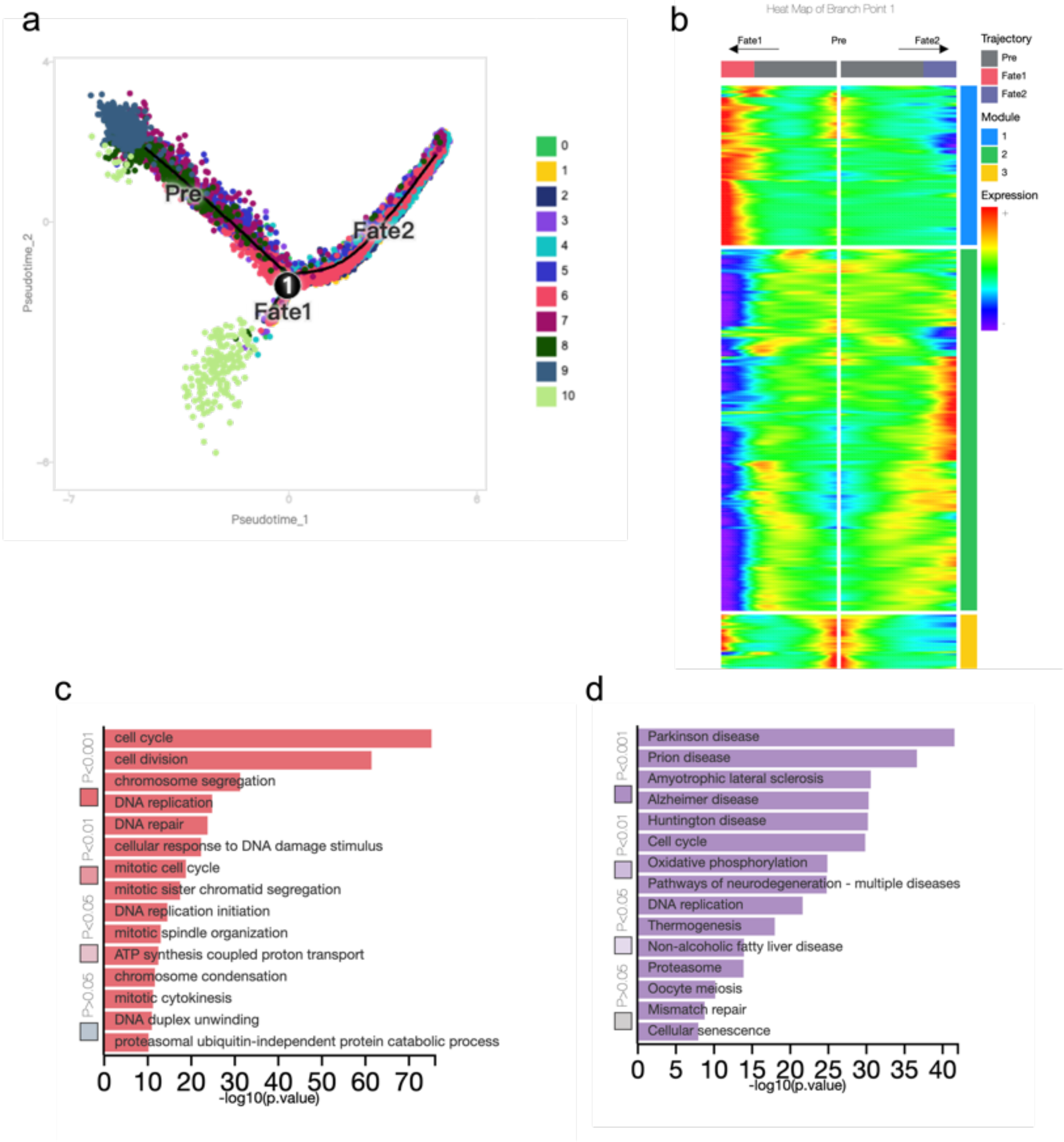

Figure S20. Pseudotime analysis of subclusters in CD4$^+$ T cells.

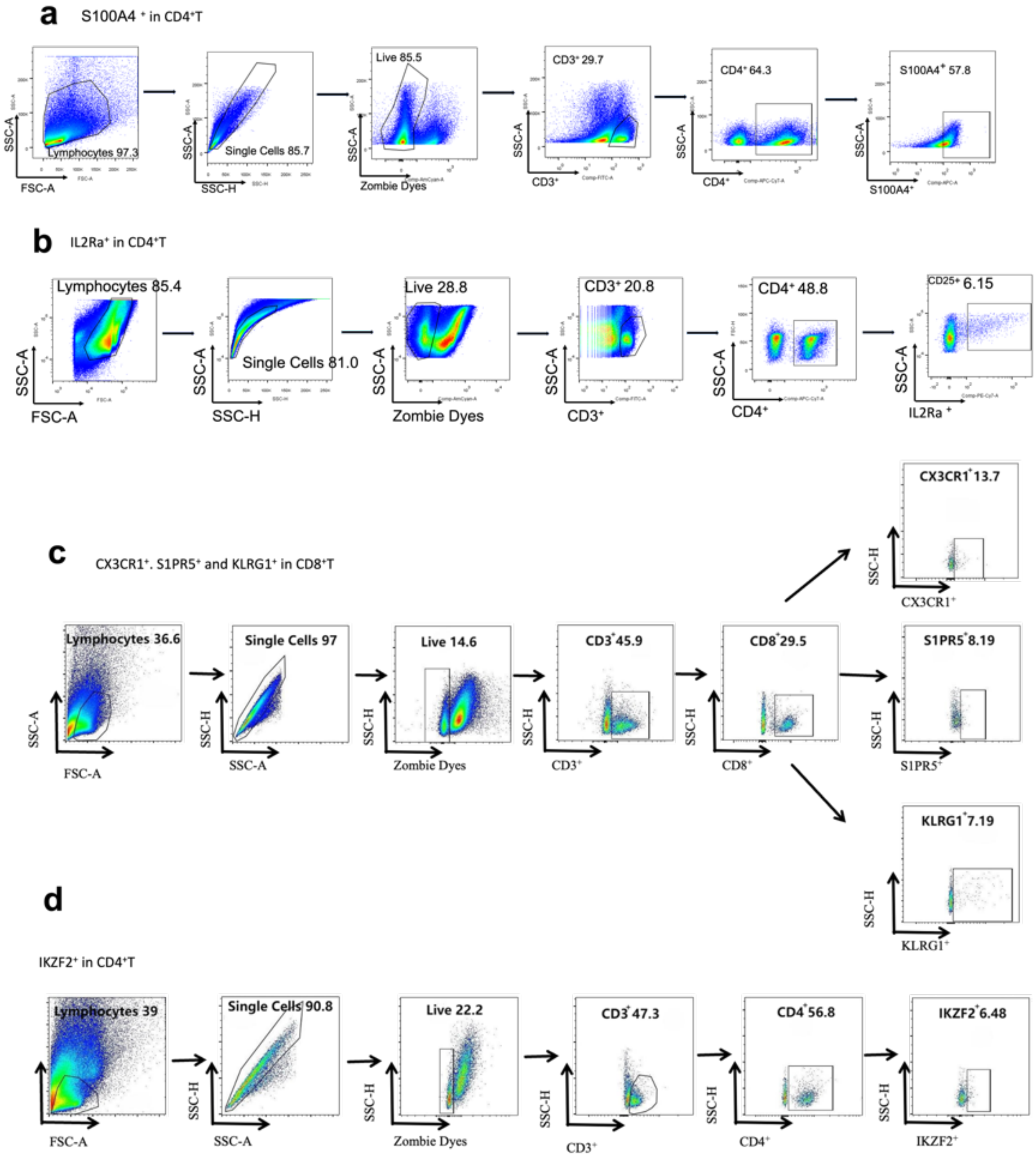

Figure S21. Flow cytometry gating strategy of featured markers in T cell populations in mice studies. a, Gating strategy of S100A4^{+} cells in CD4^{+} T cells. **b,** Gating strategy of IL2Ra^{+} cells in CD4^{+} T cells. **c,** Gating strategy of KLRG1^{+} cells, S1PR5^{+} cells and CX3CR1^{+} cells in CD8^{+} T cells. **d,** Gating strategy of IKZF2^{+} cells in CD4^{+} T cells.

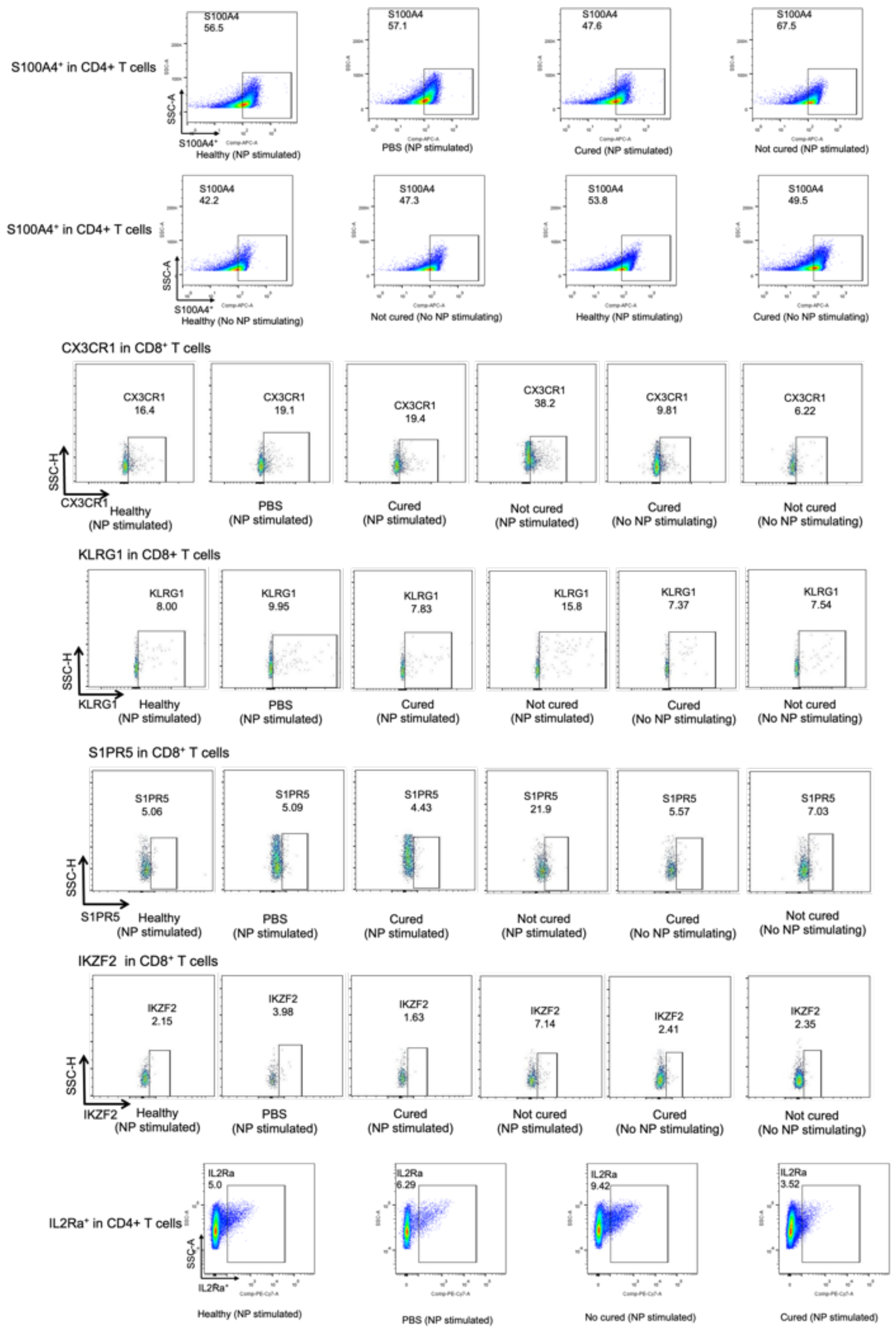

Figure S22. Representative flow cytometry results of featured markers in T cell populations in mice studies.

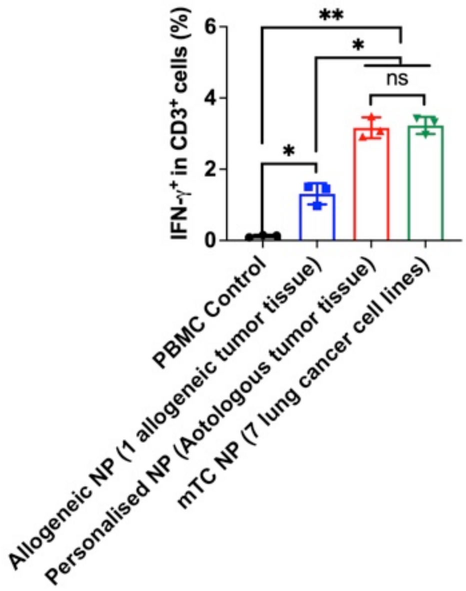

Figure S23. Comparison of nanoparticles loading autologous tumor tissues lysates and mixed cancer cells lysates in activating tumor-antigen specific T cells in human peripheral blood mononuclear cells (PBMC) from non-small lung cancer patients.

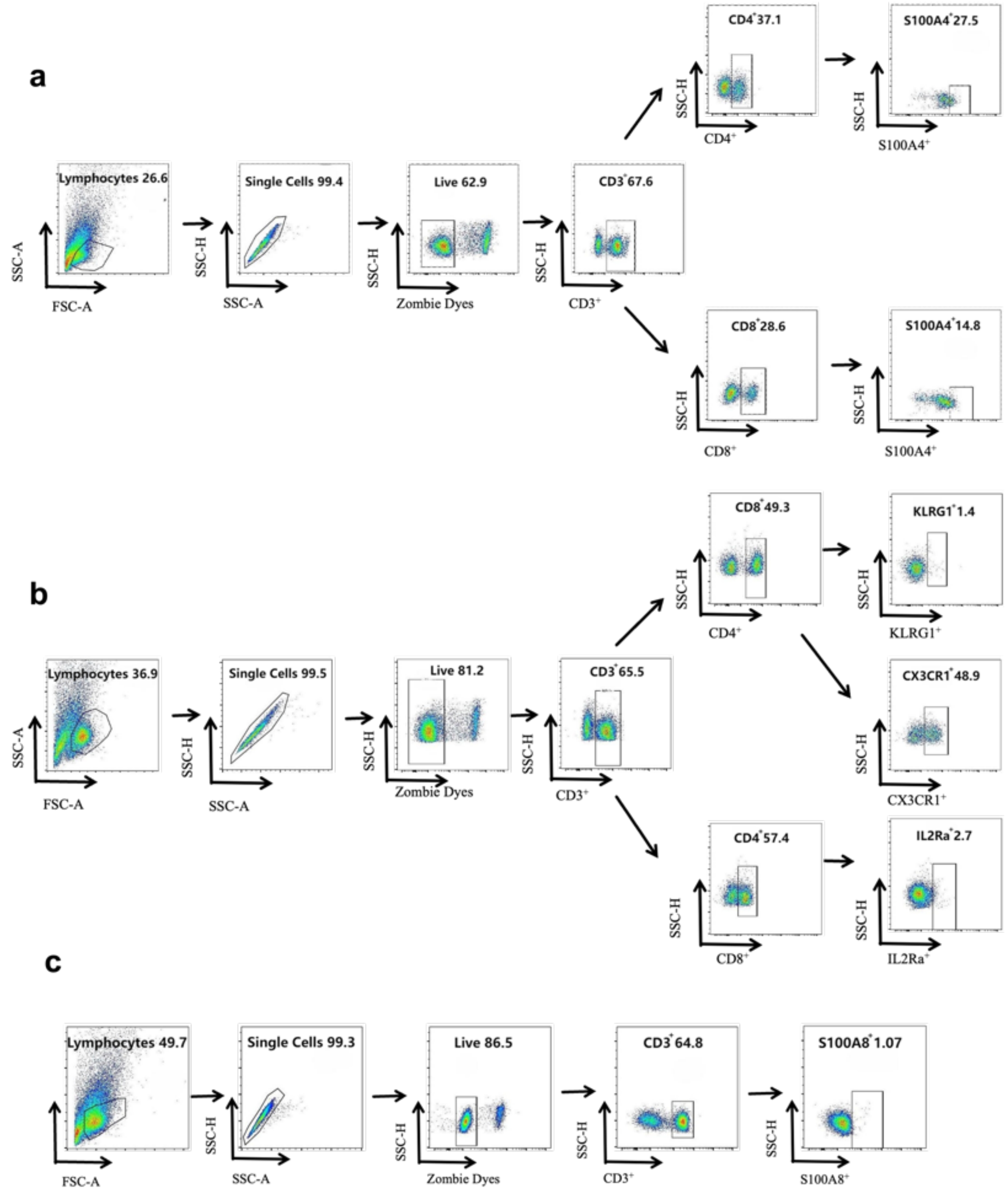

Figure S24. Flow cytometry gating strategy of featured markers in T cell populations in analysis of human peripheral blood mononuclear cells (PBMC). a, Gating strategy of S100A4$^+$ cells in CD4$^+$ T cells and CD8$^+$ T cells. **b,** Gating strategy of KLRG1$^+$ cells and CX3CR1$^+$ cells in CD8$^+$ T cells, and IL2Ra$^+$ cells in CD4$^+$ T cells. **c,** Gating strategy of S100A8$^+$ cells in CD3$^+$ T cells.

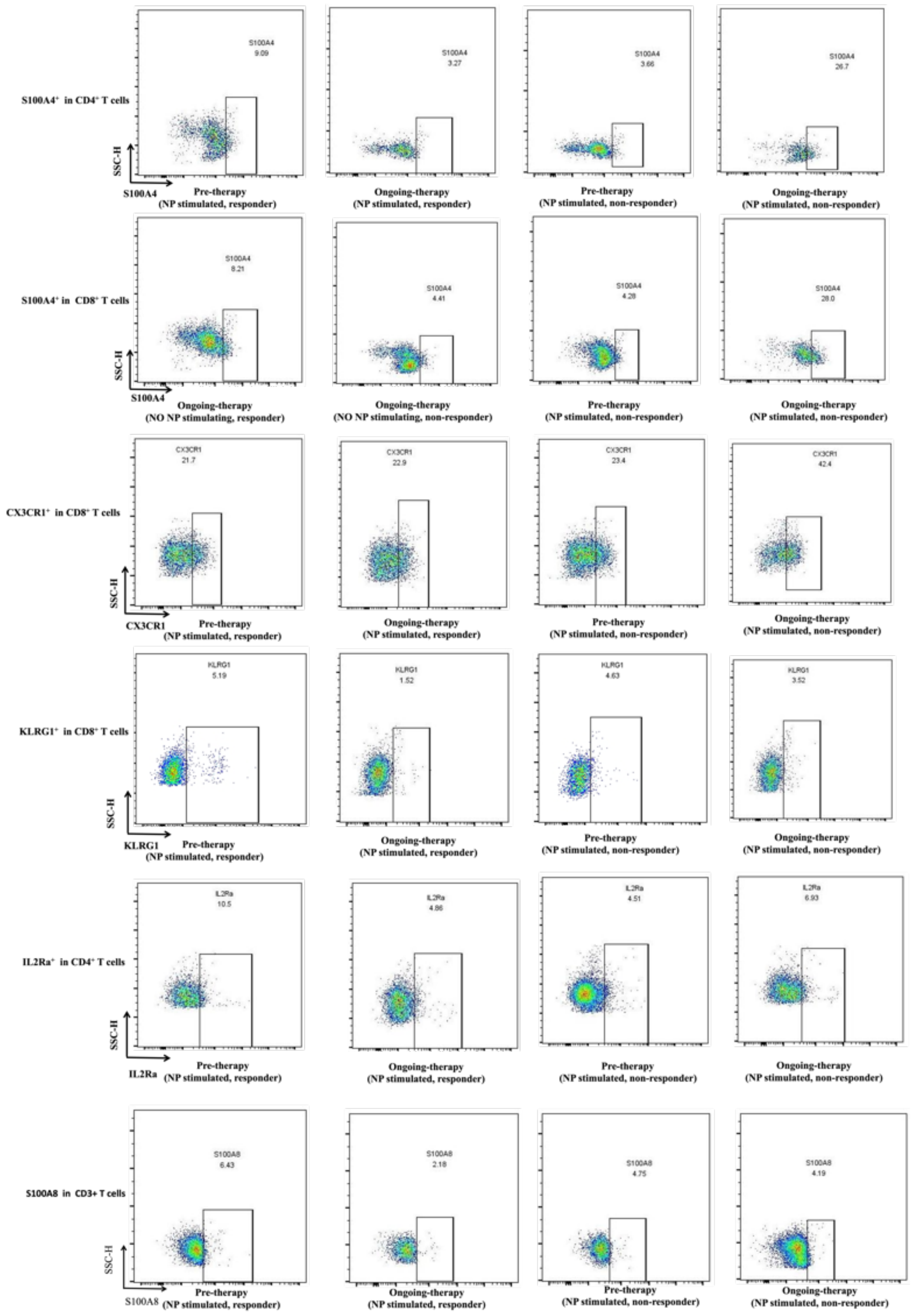

Figure S25. Representative flow cytometry results of featured markers in T cell populations in analysis of human peripheral blood mononuclear cells (PBMC).
